# Dietary Emulsifiers Alter Composition and Activity of the Human Gut Microbiota *in vitro*, Irrespective of Chemical or Natural Emulsifier Origin

**DOI:** 10.3389/fmicb.2020.577474

**Published:** 2020-11-05

**Authors:** Lisa Miclotte, Kim De Paepe, Leen Rymenans, Chris Callewaert, Jeroen Raes, Andreja Rajkovic, John Van Camp, Tom Van de Wiele

**Affiliations:** ^1^Center for Microbial Ecology and Technology (CMET), Ghent University, Ghent, Belgium; ^2^Vlaams Instituut voor Biotechnologie (VIB) Nucleomics Core, Lab of Molecular Bacteriology – Rega, KULeuven, Ghent, Belgium; ^3^Department of Food Technology, Food Safety and Health, Ghent University, Ghent, Belgium

**Keywords:** dietary emulsifiers, human gut microbiota, interindividual variablility, microbiome composition, emulsifier origin, microbiome functionality

## Abstract

The use of additives in food products has become an important public health concern. In recent reports, dietary emulsifiers have been shown to affect the gut microbiota, contributing to a pro-inflammatory phenotype and metabolic syndrome. So far, it is not yet known whether similar microbiome shifts are observable for a more diverse set of emulsifier types and to what extent these effects vary with the unique features of an individual’s microbiome. To bridge this gap, we investigated the effect of five dietary emulsifiers on the fecal microbiota from 10 human individuals upon a 48 h exposure. Community structure was assessed with quantitative microbial profiling, functionality was evaluated by measuring fermentation metabolites, and pro-inflammatory properties were assessed with the phylogenetic prediction algorithm PICRUSt, together with a TLR5 reporter cell assay for flagellin. A comparison was made between two mainstream chemical emulsifiers (carboxymethylcellulose and P80), a natural extract (soy lecithin), and biotechnological emulsifiers (sophorolipids and rhamnolipids). While fecal microbiota responded in a donor-dependent manner to the different emulsifiers, profound differences between emulsifiers were observed. Rhamnolipids, sophorolipids, and soy lecithin eliminated 91 ± 0, 89 ± 1, and 87 ± 1% of the viable bacterial population after 48 h, yet they all selectively increased the proportional abundance of putative pathogens. Moreover, profound shifts in butyrate (−96 ± 6, −73 ± 24, and −34 ± 25%) and propionate (+13 ± 24, +88 ± 50, and +29 ± 16%) production were observed for these emulsifiers. Phylogenetic prediction indicated higher motility, which was, however, not confirmed by increased flagellin levels using the TLR5 reporter cell assay. We conclude that dietary emulsifiers can severely impact the gut microbiota, and this seems to be proportional to their emulsifying strength, rather than emulsifier type or origin. As biotechnological emulsifiers were especially more impactful than chemical emulsifiers, caution is warranted when considering them as more natural alternatives for clean label strategies.

## Introduction

The current obesity crisis and related health conditions are increasingly associated with the overconsumption of so-called ultra-processed food products (Broussard and Devkota, 2016; Monteiro et al., 2017; Rauber et al., 2018; Waterlander et al., 2018; Branca et al., 2019). Food additives are characteristic elements of said products (Monteiro et al., 2013, 2017; Carocho et al., 2014) and are added to enhance, among others, shelf life, palatablility, texture, color, and nutritional value. However, the health impact of certain food additives has always been questioned (Payne et al., 2012; Carocho et al., 2014; Miclotte and Van de Wiele, 2019), and at this moment, the use of food additives in food products is one of the main public concerns about food in Europe (EFSA, 2019).

Diet is known to have a strong and fast impact on the gut microbiota (Musso et al., 2010; Martínez Steele et al., 2017; Ding et al., 2019), which is generally considered an important parameter of gut and overall health (Musso et al., 2010; Bischoff, 2011; Ding et al., 2019). An unbalanced gut microbiota is being related to several physical and mental illnesses and conditions (Ding et al., 2019). With respect to obesity and non-communicable diseases, a dysbiosed gut microbiota is characterized by a lower alpha diversity and is related to increased harvest from food and decreased fatty acid oxidation, glucose tolerance, production of satiety hormones, and intestinal barrier integrity (Musso et al., 2010).

Recently, research has emerged that ties the consumption of additives to health markers through the gut microbiota. Dietary emulsifiers in particular have been proposed to display a destabilizing impact on gut health. Chassaing et al. (2015, 2017) found *in vivo* that sodium carboxymethylcellulose (CMC) and polysorbate 80 (P80) increase gut microbial motility and lower mucus layer thickness, yielding an increased production of pro-inflammatory compounds, low-grade gut inflammation, and weight gain. Another study has linked glycerol monolaurate (GML) with signatures of metabolic syndrome together with the alterations of gut microbiota composition, among which decreased abundance of *Akkermansia muciniphila* and increased abundance of *Escherichia coli* (Jiang et al., 2018). The latter study is particularly relevant since GML is one of the World’s most widely used dietary emulsifiers (E471).

Knowledge that is currently still lacking from literature regarding the impact of dietary emulsifiers is what characteristics of an emulsifier determine its destabilizing effects, whether alternative, more natural emulsifiers could be safer and to what extent the unique features of an individual’s microbiome play a role in the purported effects on the microbiome.

The present study describes the effects of five dietary emulsifiers: CMC, P80, soy lecithin, sophorolipids, and rhamnolipids (RLs). The first two, CMC and P80 are the synthetic emulsifiers that have been used for years and both are considered safe for human oral consumption. CMC is a water soluble anionic polymer with water-binding properties, due to which it is used as a thickener, emulsifier, or water-retainer in applications like pharmaceuticals, food products, paper, cosmetics, detergents, etc. (Hercules Inc., and Aqualon, 1999; Biswal and Singh, 2004). In Europe, CMC can be used in many food products at *quantum satis* levels (European Commision, 2014), and in the United States, CMC carries the GRAS-status (generally recognized as safe) for the applications in food (FDA, 2019b, 2020). P80 is a member of the polysorbates, a group of non-ionic surfactants with applications mainly in the food, cosmetics, and pharmaceutical industries (Nielsen et al., 2016; FMI, 2020). With an acceptable daily intake of 25 mg/kg BW/day (Aguilar et al., 2015), EFSA allows its use in products like sauces, soups, chewing gum, coconut milk, dairy products, and usually at maximal concentrations of 10–10,000 mg/kg, depending on the product (European Commision, 2011). Also the United States Food and Drug Administration (FDA) limits the use of P80 to 4–10,000 mg/kg, depending on the product (FDA, 2019a).

Soy lecithin is a mixture of phospholipids (at least 60%), triglycerides, sterols, and carbohydrates obtained by extraction from soybeans. It is more widely used than CMC and P80, primarily in bakery products, ice creams, chocolate etc. (European Commision, 2018). Lecithins are allowed by EFSA in most food applications in *quantum satis* levels, and also the FDA considers soy lecithin a GRAS compound (Carocho et al., 2014; FDA, 2019c). Even though soy lecithin is considered safe or even beneficial for health (Ehehalt et al., 2010; Mourad et al., 2010; Stremmel et al., 2010), the impact of soy lecithin on the gut microbiota has never been studied. Since this compound is one of the most extensively used food emulsifiers, it was incorporated in this research.

Finally, rhamnolipids and sophorolipids are two biotechnological emulsifiers of microbial origin. Due to their advantageous properties with respect to (eco) toxicity and waste stream reuse (Haba et al., 2003; Van Bogaert et al., 2011; Costa et al., 2017), they are currently under consideration as novel food additives (Cameotra and Makkar, 2004; Costa et al., 2017; Nitschke and Silva, 2018). Their strong emulsifying capacities (Van Bogaert et al., 2011; Costa et al., 2017) and more natural origin (biotechnological production from renewable resources) could qualify them as adequate alternatives for emulsifiers of chemical origin, which the food industry is currently seeking to replace under the umbrella of the “clean label” trend (Asioli et al., 2017; Costa et al., 2017; Nitschke and Silva, 2018). However, given their strong antimicrobial properties, an evaluation at the level of the gut microbiota is highly warranted before such applications can be legalized.

Here, we investigated the effects of the five above-mentioned dietary emulsifiers on human fecal microbiota through 48 h *in vitro* batch incubations. This set of emulsifiers enables the comparison of the previously-studied chemical emulsifiers with the natural extract lecithin and with biosurfactants. In order to take into account interindividual variability in microbiome composition as a possible determinant of the putative impact from emulsifiers, we separately assessed microbial incubations from 10 different individuals.

## Materials and Methods

### Experimental Design

Fecal material from 10 human individuals was collected and separately incubated for 48 h with the five emulsifiers at three concentrations [0.005, 0.05, and 0.5% (m/v)]. Emulsifier concentrations were chosen based on the maximal legal concentration in food products (EFSA and FDA), which comply with commonly applied concentrations in food products (Mallet, 1992; Adams et al., 2004; Msagati, 2012). Each donor incubation series also included a control condition, in which a sham treatment with an equivalent volume of distilled water was performed.

The emulsifiers used during this study were sodium CMC, P80, soy lecithin, sophorolipids, and rhamnolipids. CMC (catalog number 419303: average molecular weight of 250,000 g/mol and degree of substitution of 0.9), P80 (P4780 – suitable for cell culture) and rhamnolipids (RLs: R90–90% pure) were obtained from Sigma Aldrich, St. Louis, MO, United States. Soy lecithin was obtained from Barentz Unilecithin (UNILEC–ISL non GMO IP), and sophorolipids were obtained from the UGent Inbio group from the Centre for Synthetic Biology. The latter were described as 75% (w/v) solutions, and their composition was determined to be mainly lactonic, diacetylated C18:1 SL.

Donors 2 and 6 reported to follow a vegetarian and vegan diet. All other donors consumed an omnivorous diet. The age of the four female and six male donors varied from 23 to 53 years old. None of the donors received any antibiotic treatment in the 3 months prior to their donation. Experimental work with fecal microbiota from human origin was approved by the Ethical Committee of the Ghent University hospital under the registration number BE670201836318.

### Batch Incubation

Before incubation, the five emulsifiers were supplemented to amber penicillin bottles containing 40 ml of autoclaved low-sugar nutritional medium (per L: 0.25 g gum arabic, 0.5 g pectin, 0.25 g xylan, 1 g starch, 3 g yeast extract, 1 g proteose peptone, and 2 g pig gastric mucin; all from Sigma Aldrich, St. Louis, MO). The amounts of emulsifiers to add were calculated to obtain concentrations of 0.005, 0.05, and 0.5% (m/v) in a final volume of 50 ml (the volume obtained after addition of the fecal slurry). The bottles were stored in a 4°C fridge until use (for maximum 3 days).

At the start of the batch experiments, the penicillin flasks containing nutritional medium and emulsifiers were brought to room temperature to provide an ideal growth environment for the fecal bacteria. Fresh fecal samples were then collected in airtight plastic lidded containers. AnaeroGen™ sachets (Oxoid Ltd., Basingstoke, Hampshire, UK) were used to sequester O_2_. The samples were stored at 4°C until the use for a maximum of 3 h. A fecal inoculum was then prepared as described in De Boever et al. (2000), by mixing 20% (w/v) fecal material into a 0.1 M anaerobic phosphate buffer (pH 6.8) supplemented with 1 g/L sodium thioglycanate as a reducing agent. Into each penicillin bottle, 10 ml of fecal inoculum was added, after which the bottles were closed with butyl rubber stoppers and aluminum caps. The headspaces were flushed with a N_2_/CO_2_ (80/20)-gas mixture using a gas exchange equipment to obtain anaerobic conditions and incubated in an IKA® KS 4000 I Control shaker at 200 rpm at 37°C. During the course of the experiments, the pH was followed up every day with a Prosense QP108X pH-electrode connected to a Consort C3020 multi parameter analyzer to ensure stable and viable growth conditions (pH remained within 5.5–6.8).

Aliquots were taken on three timepoints: immediately after the start of the incubation (T0; 2–3 h after combining the fecal inoculum with the medium containing emulsifier), after 24 h of incubation (T1) and after 48 h of incubation (T2). Samples were taken for short chain fatty acid (SCFA) analysis (1 ml), 16S rRNA gene amplicon sequencing (1 ml), for flagellin detection (500 μl), and for immediate fluorescent cell staining and flow cytometry (100 μl). SCFA-, flagellin-, and sequencing-samples were stored at −20°C until analysis.

### Intact/Damaged Cell Counts

To assess the impact of the emulsifiers on total and intact cell concentrations, cell staining with SYBR® green and propidium iodide was performed after which the cells were counted on an Accuri C6+ Flow cytometer from BD Biosciences Europe. The combination of these two cell stains is frequently used to distinguish intact bacterial cells from cells damaged at the level of the cell membrane, since SYBR® green enters any cell rapidly, while propidium iodide, being a larger molecule, enters intact cells much slower and mainly stains damaged cells within commonly applied incubation times (Van Nevel et al., 2013). Samples were analyzed immediately after sampling to preserve the intact cell community. Dilutions up to 10^−4^ and 10^−5^ were prepared in 96-well plates using 0.22 μm filtered 0.01 M phosphate buffered saline (PBS; HPO_4_^2−^/H_2_PO_4_^−^, 0.0027 M KCl, and 0.137 M NaCl, pH 7.4, at 25°C), and these were subsequently stained with SYBR® green combined with propidium iodide (SGPI, 100× concentrate SYBR® Green I, Invitrogen, and 50× 20 mM propidium iodide, InvitroGen, in 0.22 μm-filtered dimethyl sulfoxide; Van Nevel et al., 2013; Props et al., 2016). After 25 min of incubation, the intact and damaged cell populations were measured immediately with the flow cytometer, which was equipped with four fluorescence detectors (530/30, 585/40, >670, and 675/25 nm), two scatter detectors and a 20 mW 488 nm laser. The flow cytometer was operated with Milli-Q (Merck Millipore, Belgium) as sheath fluid. The blue laser (488 nm) was used for the excitation of the stains, and a minimum of 10,000 cells per sample were measured for accurate quantification. Settings used were an FLH-1 limit of 1,000, a measurement volume of 25 μl, and the measurement speed was set to “fast.” Cell counts were obtained by gating the intact and damaged cell populations in R (version 3.6.2) according to the Phenoflow-package (v1.1.6; Props et al., 2016). Gates were verified using data from negative control samples (only 0.22 μm filtered 0.01 M PBS; Figure S1 in Supplementary Data Sheet 1).

### SCFA-Analysis

The SCFA-concentrations were determined by means of diethyl ether extraction and capillary gas chromatography coupled to a flame ionization detector as described by De Paepe et al. (2017) and Anderson et al. (2017). Briefly, 1 ml aliquots were diluted 2× with 1 ml milli-Q water, and SCFA were extracted by adding approximately 400 mg NaCl, 0.5 ml concentrated H_2_SO_4_, 400 μl of 2-methyl hexanoic acid internal standard and 2 ml of diethyl ether before mixing for 2 min in a rotator and centrifuging at 3,000 *g* for 3 min. Upper layers were collected and measured using a GC-2014 capillary gas chromatograph (Shimadzu, Hertogenbosch, Netherlands), equipped with a capillary fatty acid-free EC-1000 Econo-Cap column (Alltech, Lexington, KY, United States), 25 m × 0.53 mm; film thickness 1.2 μm, and coupled to a flame ionization detector and split injector. One sample [donor 9, timepoint 2–0.05% (m/v) CMC] returned only zero values, presumably due to a technical error. This sample was therefore omitted prior to computational analyses.

### Amplicon Sequencing

Samples from T0 and T2 were selected for Illumina 16S rRNA gene amplicon sequencing. The samples (1 ml) were first centrifuged for 5 min at 30,130 *g* in an Eppendorf 5430 R centrifuge to obtain a cell pellet. After removing the supernatant, the pellets were subjected to DNA-extraction (Vilchez-Vargas et al., 2013; De Paepe et al., 2017). The pellets were dissolved in 1 ml Tris/HCl (100 mM, pH = 8.0) supplemented with 100 mM EDTA, 100 mM NaCl, 1% (w/v) polyvinylpyrrolidone, and 2% (w/v) sodium dodecyl sulfate after which 200 mg glass beads (0.11 mm Sartorius, Gottingen, Germany) were added, and the cells were lysed for 5 min at 2,000 rpm in a FastPrep VR-96 instrument (MP Biomedicals, Santa Ana, CA). The beads were then precipitated by centrifugation for 5 min at 30,130 *g*, and the supernatant was collected. Purification of DNA took place by the extraction of cellular proteins with 500 μl phenol-chloroform-isoamilyc alcohol 25-24-1 at pH7 and 700 μl 100% chloroform. The DNA was precipitated by adding 1 volume of ice-cold isopropyl alcohol and 45 μl sodium acetate and cooling for at least 1 h at −20°C. Isopropyl alcohol was then separated from DNA by centrifugation for 30 min at 4°C and at 30,130 *g*, and the pellet was dried by pouring off the supernatant. It was resuspended in 100 ml 1× TE buffer (10 mM Tris, 1 mM EDTA) for storage at −20°C.

DNA-quality was verified by electrophoresis in a 1.5% (w/v) agarose gel (Life technologies, Madrid, Spain), and DNA-concentration was determined using a QuantiFluor® dsDNA kit (detection limit: 50 pg/ml; sensitivity: 0.01–200 ng/μl) and GloMax®-Multi+ system (Promega, Madison, WI) with the blue fluorescence optical kit installed (Ex: 490 nm and Em: 510–570 nm).

Library preparation and next generation 16S rRNA gene amplicon sequencing were performed at the VIB nucleomics core (VIB, Gasthuisberg Campus, Leuven, Belgium) as described in Tito et al. (2017). The V4 region of the 16S rRNA gene was amplified using the bacterial 515F (GTGYCAGCMGCCGCGGTAA) and the 806R (GGACTACNVGGGTWTCTAAT) primers, which were modified with both Illumina adapters as well as adapters for directional sequencing. Sequencing was then performed on an Illumina MiSeq platform (Illumina, Hayward, CA, United States) according to manufacturer’s guidelines.

One sample [donor 3, timepoint 2, 0.05% (m/v) sophorolipids] failed to sequence. The sequencing data have been submitted to the National Center for Biotechnology Information (NCBI) database under the accession number PRJNA630547.

Processing of amplicon data was carried out using mothur software version 1.40.5 and guidelines (Kozich et al., 2013). First, contigs were assembled, resulting in 14,977,727 sequences, and ambiguous base calls were removed. Sequences with a length of 291 or 292 nucleotides were then aligned to the silva_seed nr.123 database, trimmed between positions 11,895 and 25,318 (Quast et al., 2013). After removing the sequences containing homopolymers longer than nine base pairs, 92% of the sequences were retained resulting in 2,957,626 unique sequences. A pre-clustering step was then performed, allowing only three differences between sequences clustered together and chimera. vsearch was used to remove chimeras, retaining 79% of the sequences. The sequences were then classified using a naïve Bayesian classifier against the Ribosomal Database Project (RDP) 16S rRNA gene training set version 16, with a cut-off of 85% for the pseudobootstrap confidence score. Sequences that were classified as *Archaea*, *Eukaryota*, *Chloroplasts*, unknown, or *Mitochondria* at the kingdom level were removed. Finally, sequences were split at the order level into taxonomic groups using the OptiClust method with a cut-off of 0.03. The data were classified at a 3% dissimilarity level into OTUs resulting in a .shared (count table) and a .tax file (taxonomic classification).

For the entire dataset of 319 samples, 95,511 OTUs were detected in 175 genera. An OTU was in this manuscript defined as a collection of sequences with a length between 291 and 292 nucleotides and with 97% or more similarity to each other in the V4 region of their 16S rRNA gene after applying hierarchical clustering.

### Cell Culture for Flagellin Detection

Murine TLR5-expressing HEK 293 cells (InvivoGen), which are designed to respond to bacterial flagellin in cell culture medium, were cultured according to the manufacturer’s guidelines. Cells were grown from an in house created frozen stock in Dulbecco’s modified Eagle’s growth medium (DMEM; 4.5 g/L glucose, 10% fetal bovine serum, 50 U/ml penicillin, 50 μg/ml streptomycin, and 2 mM L-glutamine) supplemented with 100 μg/ml Normocin™ and maintained in culture in DMEM growth medium supplemented with 100 μg/ml Normocin™, 10 μg/ml of blasticidin, and 100 μg/ml of Zeocin™. Medium was refreshed every 2 days, and the cells were passaged when reaching 70–80% confluency.

Assays for flagellin detection were performed as instructed by InvivoGen, using cells from the passages 4–9. Samples from donors 3, 5, and 7 were selected for this assay based on a high, intermediate, and low metabolic responses to the emulsifiers, as measured by the SCFA-levels. Before combining with the HEK-blue cells, the samples were purified to obtain only the bacterial cells by first diluting them one-fourth in UltraPure™ DNase/RNase-Free distilled water (InvitroGen), then centrifuging twice at 4,226 *g* for 10 min, with a washing step using 0.22 μm filtered PBS in between. The resulting cell pellet was dissolved into 0.22 μm filtered PBS. A standard curve (1.25–1.95 ng/ml), prepared from recombinant flagellin from *Salmonella typhymurium* (RecFLA-ST, InvivoGen) in sterile water was also added to the plate in triplicate. After an incubation for 23 h, absorbances were obtained using a Tecan Infinite F50 plate reader at 620 nm.

As a check for the viability of the cell culture after combination with the samples, a resazurin assay was performed. To this end, the supernatant from the cell culture plate used for the flagellin assay was discarded after the first incubation phase. The cells were then washed using 0.22 μm filtered PBS. For the detection part of the assay, three wells were spiked with 20 µl of a 5% Trition solution, as a positive control, while the rest of the wells received 20 µl PBS. Then, 180 µl of a 0.01 µg/ml resazurin solution was added to all wells. After 3 h of incubation at 37°C and 10% CO_2_, cell activity was measured using a Glomax®-Multi1 system (Promega, Madison, WI) with filter the green fluorescence optical kit (Ex: 525 nm and Em: 580–640 nm).

### Data Analysis and Statistics

Data visualization and processing was performed in R version 3.4.2 (2017-09-28; R Core Team, 2016) and Excel 2016. All hypothesis testing was done based on a significance level of 5% (*α* = 0.05).

#### Cell Counts and SCFA

After loading the cell count table in R, total cell counts were calculated as the sum of the intact and damaged cell counts. The data were explored by calculating intact/damaged cell count ratio’s and percentages of intact cells at different timepoints. For plotting, both the 10,000× and 100,000× dilutions were taken into account. Boxplots of total and intact cell counts, as well as intact/damaged ratio’s were created using ggplot2 (v3.2.1) in which the stat_compare_means function was used to check the significance of the emulsifier effect, by means of a Kruskal–Wallis test.

Statistical analysis of SCFA-levels was similar to that of the cell counts. Production levels of acetate, propionate, and butyrate over 48 h (C_T2_–C_T0_) were first calculated, and then the boxplots were created using ggplot2. Significance of the effect of the emulsifier treatment was tested with stat_compare_means using Wilcoxon Rank Sum tests with Holm’s correction and Kruskal–Wallis test for overall group comparison.

#### Amplicon Sequencing Data

The shared and taxonomy files resulting from the mothur pipeline were loaded into R for further processing. Singletons (OTUs occurring only once over all samples) were removed, resulting in 36,496 OTUs being retained (McMurdie and Holmes, 2014). Rarefaction curves were created to evaluate the sequencing depth (Figure S2 in Supplementary Data Sheet 1; Oksanen et al., 2019). As the number of 16S rRNA gene copies present within bacteria differs between species, a copy number correction of the reads was carried out by first classifying the representative sequences of the OTUs (also obtained *via* the mothur pipeline) using the online RDP classifier tool, then obtaining both a copy number corrected read classification and a non-copy number corrected one, calculating the copy number by dividing both and finally dividing the acquired read counts in the shared file by the calculated copy numbers.

Both relative and absolute abundances of the OTUs and genera were calculated from the copy number corrected read counts and were explored *via* bar plots using ggplot2 (v3.2.1). Relative abundances were calculated as percentages of the total read counts per sample. Absolute abundances were calculated (quantitative microbial profiling) by multiplying the total cell counts obtained *via* flow cytometry with the relative abundances of the OTUs (similar to Vandeputte et al., 2017).

Overall community composition was visualized using principle coordinate analysis (PcoA) on the abundance based Jaccard distance matrix using the cmdscale-function in the stats (v3.6.2) package. To investigate the effects of the individual constraints on the microbial community, a series of distance based redundancy analyses (dbRDAs) was then performed on the scores obtained in the PCoA on the Jaccard distance matrix using the capscale function in the vegan (v2.5.6) package. Permutation tests were used to evaluate the significance of the models and of the explanatory variables (De Paepe et al., 2018). The global model included the factors emulsifier, emulsifier concentration, timepoint, and donor as explanatory variables and the absolute abundances of the genera as explanatory variables. In a first dbRDA, this full model was included, to investigate the share of variance explained by each constraint variable. The timepoint factor was distinguished as the factor causing the largest share of variance, and since its effect was of little interest to us it was partialled out in further dbRDAs. To check for the effect of the donor variable on the microbial community, second and third dbRDAs were performed, with and without conditioning of the donor variable. The final model then visualized the effects of the treatments (defined by factors emulsifier and emulsifier concentration). The results of the dbRDAs were plotted as Type II scaling correlation triplots showing the two first constrained canonical axes (labeled as dbRDA Dim 1/2) and the proportional constrained eigenvalues representing their contribution to the total (both constrained and unconstrained) variance.

The Chao1, ACE-1, Shannon, Simpson, InvSimpson, and Pielou diversity indices were calculated for the microbial community after 48 h incubation based on the copy number corrected OTU-table using the SPECIES (v1.0) package and the diversity function in the vegan (v2.5.6) package. Indices were plotted using ggplot2, and significances were tested using pairwise Wilcoxon Rank Sum tests with Holm’s correction (ggpubr package v.0.2.4).

To evaluate differential abundance of genera between emulsifier treatments and control, the DESeq2 package (v 1.24.0) was applied on the copy number corrected count-table at genus level. In order to streamline the DESeq-analysis, pre-filtering according to McMurdie and Holmes (2014) was first applied on the copy number corrected count-table, after which a genus-level table was created using the aggregate function (stats package v3.6.3). In the generalized linear model, the factor timepoint, donor, and treatment – a concatenation of the emulsifiers and their concentrations – were included. A likelihood ratio test was employed within the DESeq function on the reduced model, containing only the factors donor and timepoint, to test for the significance of the model. Low count genera were subjected to an empirical Bayesian correction (Love et al., 2014). For pairwise comparison of treatments versus controls, Wald tests were used after shrinkage of the Log2FoldChange (L2FC) values by means of the lfcShrink function. Values of *p* were adjusted by means of a Benjamini-Hochberg procedure (Love et al., 2014). Results were visualized in volcanoplots, displaying the −log(adjusted *p*-value) versus the Log2FoldChange of each genus. Additionally, box plots were created showing the log-transformed pseudocounts extracted by the plotCounts function for each genus that showed significant differential abundance. Since for CMC and P80, no significantly altered genera were found, and these emulsifiers were omitted from the boxplots.

Finally, to summarize relations between the emulsifier treatments and the intact cell counts, the SCFA-data and the 16S rRNA sequencing data, a partial redundancy analysis was carried out performed using the rda function in the vegan package (v2.5.6). The acetate, propionate, and butyrate concentrations, the intact cell counts and the relative abundance of the genera were set as response variables and the factors emulsifier, emulsifier concentration, donor, and timepoint as explanatory variables. Since the response variables carried different units, they were first centered on their mean using the scale function (base R v3.6.2). The factors donor and timepoint were partialled out to visualize solely the effect of the emulsifier treatments. The statistical significance of the effects was tested *via* a permutation tests, and the results were plotted in a Type II correlation triplot showing the first two constrained canonical axes (RDA1/2) annotated with their proportional eigenvalues representing their contribution to the constrained variance. The sites were calculated as weighed sums of the scores of the response variables.

#### Metagenome Prediction

Indications of functionality from phylogenetic information were obtained using PICRUSt (Phylogenetic Investigation of Communities by Reconstruction of Unobserved States; Langille et al., 2013). An OTU-table was first generated against the Greengenes reference database (v13.8) using a closed ref OTU-picking strategy. The obtained OTU-table was then run through PICRUSt’s normalize_by_copy_number.py script (Langille et al., 2013), which divides the abundance of each OTU by its inferred 16S copy number (the copy number is inferred from the closest genome representative for a 16S Greengenes reference sequence). The metagenome was then predicted using Kyoto Encyclopedia of Genes and Genomes (KEGG) database (Kanehisa et al., 2012). The prediction provided an annotated table of predicted gene family counts for each sample, where gene families were grouped by KEGG orthology identifiers. Significantly different L2-level pathways across emulsifier concentration were visualized in boxplots using the ggplot2 package (v3.2.1). Kruskal–Wallis tests were performed for the overall comparison of emulsifier concentrations within L2-pathways for each emulsifier and Wilcoxon rank sum tests were used for pairwise comparison of emulsifier concentrations vs. control. Also relying on PICRUSt (Langille et al., 2013), the BugBase tool (Ward et al., 2017) was used to determine the relative degree of biofilm formation, oxygen utilization, pathogenic potential, oxidative stress tolerance, and Gram-stain of the bacteria in the samples.

#### Flagellin Concentration

Initial data processing was executed in Excel 2016. First, a four-parametric logistic model was fitted to the standard curve using the 4PL-Curve Calculator from aatbio.com (AAT Bioquest., 2019). Given our observation that the emulsifiers decreased HEK cell activity, flagellin concentrations were normalized by use of absorbance values obtained from the resazurin assays; flagellin concentrations were divided by the ratio of the absorbance values from the samples over the average absorbance values for the standard curve of the same plate. Graphs were created using ggplot2. Since donors were observed separately and no replicate experiments per donor were performed, no statistical tests were performed for the flagellin data.

### Donor Diversity Analysis

Next to the clustering of the donors in the dbRDA described above, we sought to assess the degree of susceptibility of the 10 donors to the effects of the emulsifiers, in an attempt to identify overall more or less susceptible individuals. Since literature describes no workflow for this purpose, we elaborated our own. Donors were ranked in terms of their susceptibility to the emulsifiers using several parameters: the 48 h production of the three most abundant SCFAs (acetate, propionate, and butyrate), the intact cell counts at T2, and the relative and absolute abundance of the most abundant OTU in the OTU-table, *Escherichia*/*Shigella*, at T2. These calculations were performed in Excel 2016.

First, to correct for the batch-effect, the control-values were subtracted from the treatment-values for each donor. Next, the corrected treatment values were summated for each donor, to obtain a single value that expressed the donor’s susceptibility, and these values were then used to rank the donors from least to most susceptible for every parameter, visualized in bar graphs. This workflow was followed for each parameter.

### Comparison of Equivalent Emulsifier Concentrations

Due to their stronger emulsifying properties, rhamnolipids and sophorolipids could reportedly be used in lower concentrations in food products than conventional chemical emulsifiers (Nitschke and Silva, 2018). Therefore, we sought to compare the effects of the chemical emulsifiers, CMC and P80, versus those of the biosurfactants, rhamnolipids, and sophorolipids, with regard to their impacts on the gut microbiota at equivalent emulsifying concentrations. Wilcoxon Rank Sum test was executed in R using the compare_means function on the same parameters we used to evaluate donor diversity (see Donor Diversity Analysis section). As equivalent concentrations we considered a 10× lower concentration of biosurfactants compared to the chemical emulsifiers, given that this is what industry reports (Van Haesendonck and Vanzeveren, 2006). Hence, we compared the condition of 0.5% (m/v) of chemical emulsifiers with that of 0.05% (m/v) of biosurfactancs and 0.05% (m/v) of chemical emulsifiers with 0.005% (m/v) of biosurfactants.

## Results

### Community Structure

#### Intact/Damaged Cell Counts

**Figure 1:**
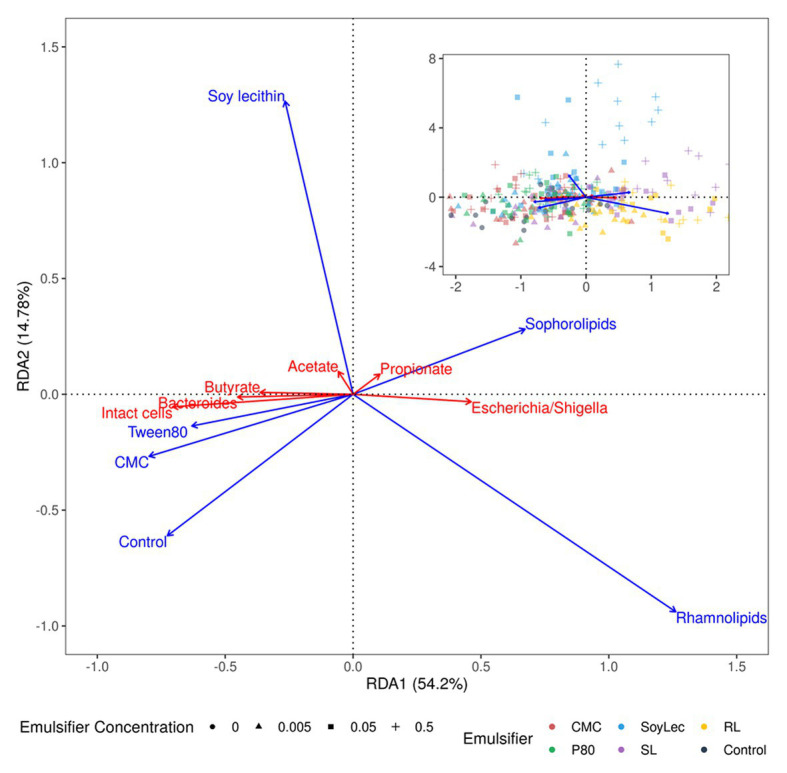
Type II scaling triplot obtained using partial redundancy analysis of the microbial community detected after 48 h of *in vitro* batch incubations of fecal material from 10 human donors in sugar depleted medium supplemented with five emulsifiers at four concentrations. In the main figure, the intact cell count, the short chain fatty acid (SCFA)-levels, and the relative abundance of the top two genera are shown as response variables (red arrows), and emulsifier concentration are given as explanatory variables (blue arrows). The top right figure also displays the samples as sites. Axes are annotated with their contribution to the total variance.

**Figure 2:**
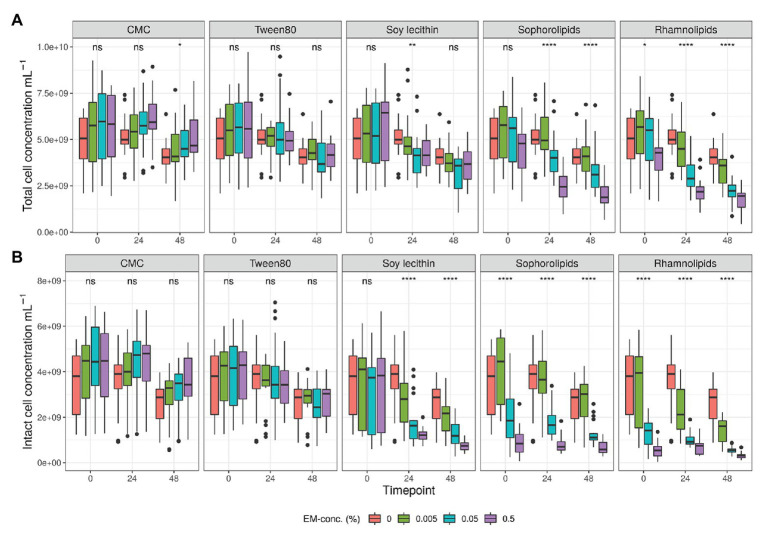
Average total **(A)** and intact **(B)** bacterial cell counts (cells/ml) detected during *in vitro* batch incubations of fecal material from 10 donors with sugar depleted medium supplemented with five emulsifiers at four concentrations. Samples were taken upon incubation (T0; 2-3 h after inoculation) as well as after 24 h (T1) and 48 h (T2) of incubation. Asterisks indicate significant differences detected with a Kruskal–Wallis test (*α* = 0.05).

Analysis of intact and damaged cell populations with flow cytometry (SGPI-staining defines damage at the level of the cell membrane; Falcioni et al., 2008; Wlodkovic et al., 2009; Buysschaert et al., 2018), was used as a proxy for emulsifier toxicity. First, total and intact cell counts in the controls dropped by 14 ± 2 and 21 ± 3%, respectively, after 48 h *in vitro* incubation due to exhaustion of nutritional medium (Tables S1, S2 in Supplementary Data Sheet 1). When considering the impact of the emulsifiers, we observed that higher concentrations of rhamnolipids, sophorolipids, and soy lecithin resulted in significantly lower total and intact cell counts (Figures 1, 2 and Table S3 in Supplementary Data Sheet 1). At 0.5% (m/v) rhamnolipids, intact cells decreased by 91 ± 0% after 48 h compared to the control sample of T0. At 0.5% (m/v) sophorolipids, about 89 ± 1%, was lost and at 0.5% (m/v) soy lecithin, 87 ± 1% was lost. The toxic effects were immediate for the sophorolipids and rhamnolipids, while for soy lecithin this decreasing effect only became significantly apparent after 24 h (T1; Figure 2). The impact of CMC and P80 toward the cell population was less pronounced. CMC even increased the total cell counts (not significantly) at higher concentrations, although the fraction of living cells remained unaffected for all CMC-conditions.

#### Microbial Community

The impact from the emulsifiers toward microbial community structure was assessed with 16S rRNA gene amplicon sequencing. First, the *in vitro* conditions had an impact on microbiota composition. While each donor showed a unique profile of microbial genera at the start of the experiment, more similar microbial community profiles were obtained upon incubation, primarily due to an increase in *Escherichia*/*Shigella* abundance from 0.02 ± 0.02 to 16 ± 25%, an increase in *Bacteroides* from 21 ± 17 to 42 ± 20%, a decrease in *Faecalibacterium* from 21 ± 16 to 1 ± 1% and a decrease in *Prevotella* from 13 ± 23 to 2 ± 4% (Figures S3–S5 in Supplementary Data Sheet 1). These alterations can be attributed to the feed composition, in which the relative enrichment in protein as well as the low carbohydrate concentration may have favored the growth of protein loving *Escherichia*/*Shigella* and versatile *Bacteroides* species over the more specialized and carbohydrate-loving *Prevotella* and *Faecalibaterium* species (Mu et al., 2016; Yao et al., 2016; Diether and Willing, 2019; Verhoog et al., 2019).

**Figure 3:**
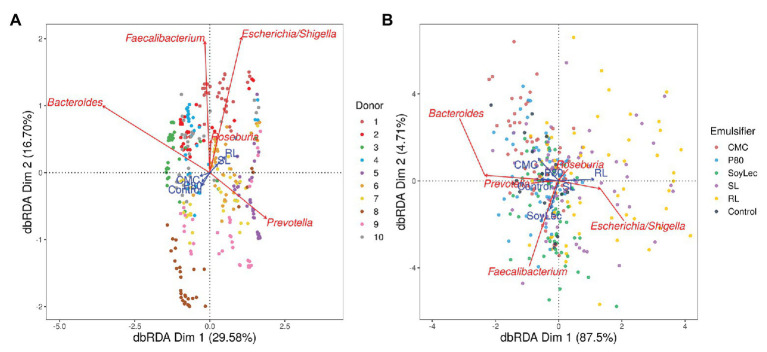
Type II scaling triplots obtained using partial distance based redundancy analysis of the microbial community composition detected using 16S rRNA gene amplicon sequencing after 48 h of *in vitro* batch incubations of fecal material from 10 donors with sugar depleted medium supplemented with five emulsifiers at four concentrations. Samples were taken upon incubation (T0; 2–3 h after inoculation) as well as after 24 h (T1) and 48 h (T2) of incubation. Factors donor, emulsifier, and emulsifier concentration were set as explanatory variables (blue arrows) and absolute abundances of genera as response variables (red arrows). Only the top five genera were displayed for adequate visibility. Axes are annotated with their contribution to the total variance. **(A)** The factor timepoint was partialled out. **(B)** The factors donor and timepoint were partialled out.

**Figure 4:**
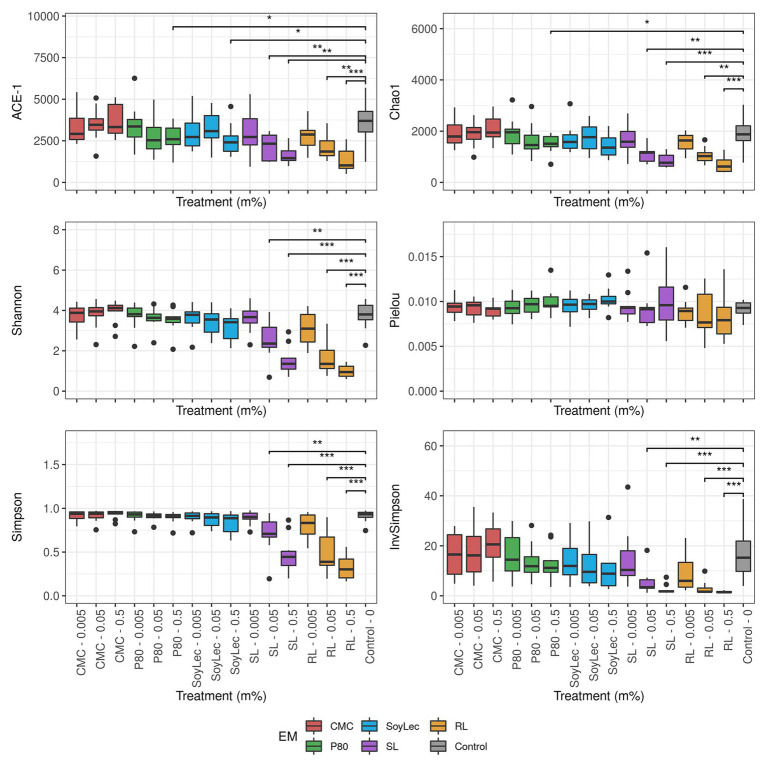
Diversity parameters of gut microbial community obtained after 48 h of *in vitro* batch incubations of fecal material from 10 donors with sugar depleted medium supplemented with five emulsifiers at four concentrations. Samples were taken upon incubation (T0; 2–3 h after inoculation) as well as after 24 h (T1) and 48 h (T2) of incubation. Asterisks represent significant differences with control based on Wilcoxon Rank Sum tests with Holm’s correction (*α* = 0.05).

**Figure 5:**
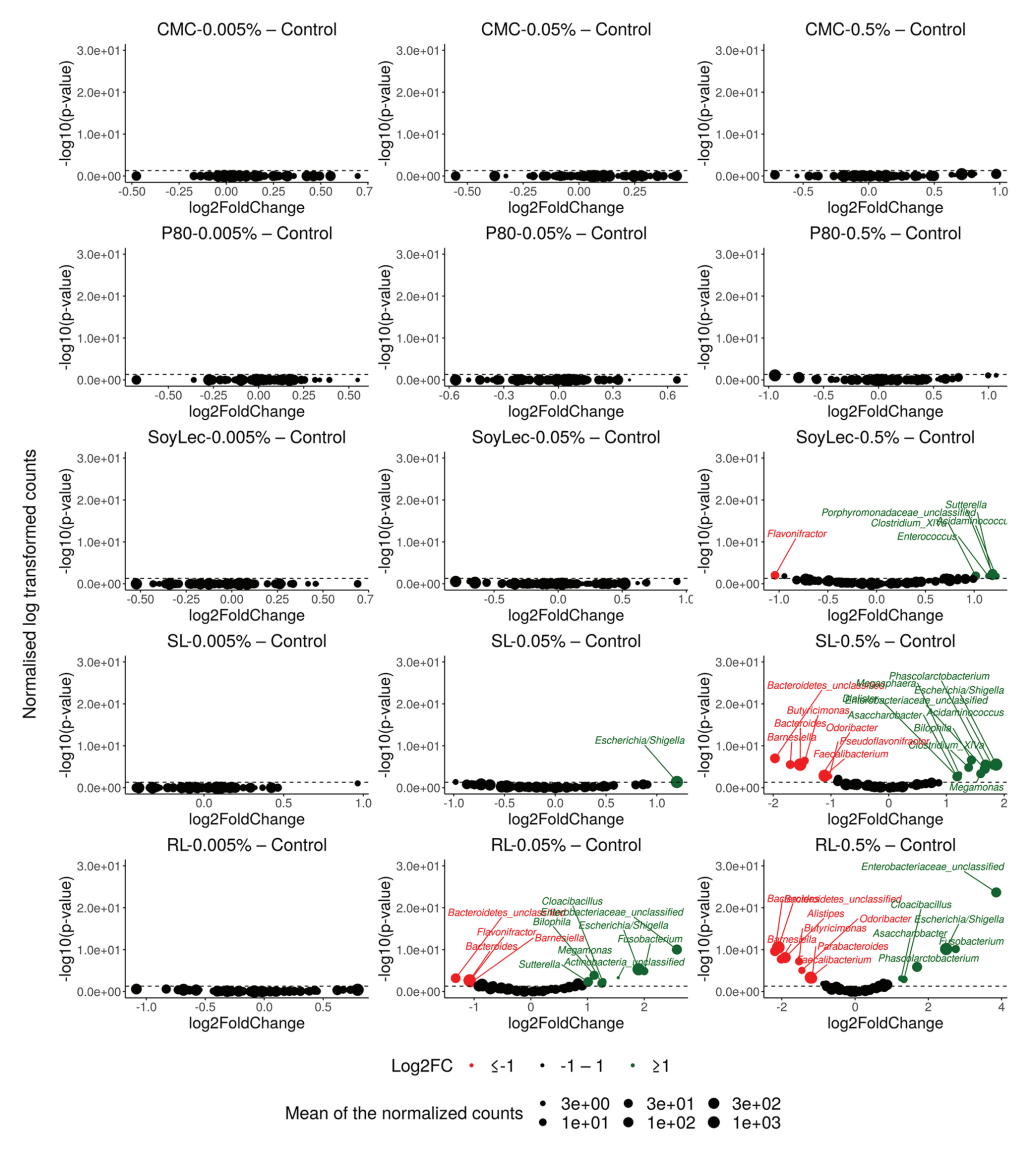
Volcano plots indicating gut microbial community alterations after 48 h of *in vitro* batch incubations of fecal material from 10 donors with sugar depleted medium supplemented with five emulsifiers at four concentrations. Samples were taken upon incubation (T0; 2–3 h after inoculation) as well as after 24 h (T1) and 48 h (T2) of incubation. Log2FoldChange (L2FC) of genus abundances for all emulsifier treatments vs. the control are presented on the *x*-axis and the log transformed adjusted *p*-value is presented on the *y*-axis. Significantly increased or decreased genera are indicated in respectively green and red. The dashed line represents the significance threshold of *α* = 0.05.

**Figure 6:**
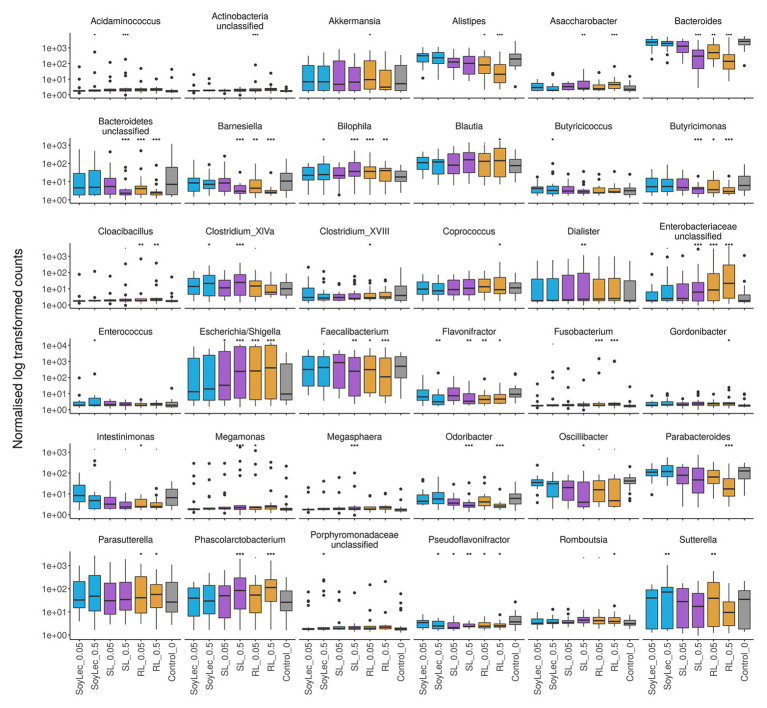
Copy number corrected counts of significantly increased or decreased genera, obtained from DESeq analysis in R (version 3.4.2), after 48 h of exposure of gut microbial communities from 10 donors to soy lecithin, sophorolipids, and rhamnolipids during *in vitro* batch incubations. Asterisks represent significant differences with the control based on Wald tests (*α* = 0.05).

Independent from the *in vitro* effects, clear differences were noted between emulsifier treatments and controls, which were both emulsifier‐ and donor-dependent (Figure 3 and Supplementary Figures S4, S5 in Supplementary Data Sheet 1). Where the effects of rhamnolipids and sophorolipids were most outspoken, the impact of soy lecithin was intermediary, and CMC and P80 had the smallest impacts (Figure 1). This was evidenced by significant drops in diversity indices upon incubation with rhamnolipids, sophorolipids and to a lesser extent soy lecithin (Figure 4). DESeq-analysis further revealed significant differential relative abundance of 36 genera, from which 23 were increased and 13 were suppressed, compared to the control condition (Figures 5, 6). Rhamnolipids triggered the strongest changes, with the three most increased genera being unclassified *Enterobacteriaceae* (L2FC = 3.85; *p*adj < 0.0001), *Fusobacterium* (L2FC = 2.75; *p*adj < 0.0001), and *Escherichia*/*Shigella* (L2FC = 2.49; *p*adj < 0.0001) and the three most suppressed ones being unclassified *Bacteroidetes* (L2FC = −2.19; *p*adj = <6,323E-4), *Barnesiella* (L2FC = −2.09; *p*adj < 0.009), and *Bacteroides* (L2FC = −2.02; *p*adj < 0.002). The top three most increased genera for sophorolipids were *Escherichia*/*Shigella* (L2FC = 1.86; *p*adj < 0.043), *Acidaminococcus* (L2FC = 1.80; *p*adj < 0.0001), and *Phascolarctobacterium* (L2FC = 1.68; *p*adj = <0.0001) and the three most decreased were unclassified *Bacteroidetes* (L2FC = −1.97; *p*adj < 0.0001), *Barnesiella* (L2FC = −1.70; *p*adj = <0.0001), and *Bacteroides* (L2FC = −1.53; *p*adj = 3.034E-06). The top three most increased genera by soy lecithin were *Acidaminococcus* (L2FC = 1.23; *p*adj = 0.016), *Porphyromonadaceae_unclassified* (L2FC = 1.19; *p*adj = 0.017), and *Sutterella* (L2FC = 1.19; *p*adj = 0.004). Two significantly decreased genera were *Flavonifractor* (L2FC = −1.04; *p*adj = 0.009) and *Pseudoflavonifractor* (L2FC = −0.95; *p*adj = 0.015; Figures 5, 6).

### Functional Analysis

#### Short Chain Fatty Acid

**Figure 7:**
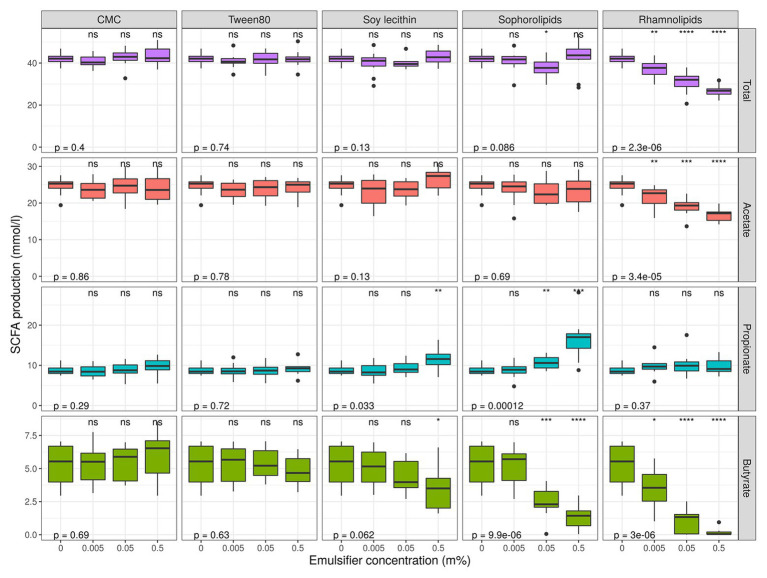
Short chain fatty acid production levels over 48 h of incubation of fecal material from 10 donors in sugar depleted medium supplemented with five emulsifiers at four concentrations. Samples were taken upon incubation (T0; 2–3 h after inoculation) as well as after 24 h (T1) and 48 h (T2) of incubation. Asterisks indicate significant differences with the control [0% (m/v)], calculated with Wilcoxon Rank Sum tests with Holm’s correction. *p*-values indicate results of general Kruskal–Wallis tests (*α* = 0.05).

Short chain fatty acids were analyzed to study how exposure to dietary emulsifiers affects the general microbial metabolic activity. We observed that SCFA-production was significantly and differently affected by rhamnolipids, sophorolipids, and soy lecithin, while no changes were observed for P80 and CMC (Figures 1, 7). The strongest impacts were noted for rhamnolipids, which, at 0.5% (m/v), significantly decreased total SCFA production by about 36 ± 5% (*p*_Wilcox_ < 0.0001) compared to the control condition. This decrease was mainly attributed to a 32 ± 7% decrease in acetate production (*p*_Wilcox_ < 0.0001) compared to the control. Rhamnolipids at 0.5% (m/v) also reduced butyrate production by 96 ± 6% compared to the control condition (*p*_Wilcox_ < 0.0001) while propionate production remained unaffected. Interestingly, incubation with 0.5% (m/v) sophorolipids also resulted in a decrease in butyrate production by 73 ± 24% compared to the control (*p*_Wilcox_ < 0.0001), while propionate production increased by 88 ± 50% (*p*_Wilcox_ = 2.1e-04). Soy lecithin at 0.5% (m/v) significantly increased propionate production by 29 ± 18% on average (*p*_Wilcox_ = 0.0089) and decreased butyrate production non-significantly by 34 ± 25% on average (*p*_Wilcox_ = 0.035). No profound shifts in microbial fermentation activity were observed for incubations with CMC and P80.

#### Metagenomic Prediction

Other emulsifier related functional shifts were explored *via* metagenomic prediction using PICRUSt. These analyses predicted suppressing effects of rhamnolipids, sophorolipids on the pathways “Biosynthesis of other secondary metabolites,” “Cell growth and death,” and “Signalling molecules and interaction” (Figure S6 in Supplementary Data Sheet 1). Possible significantly upregulated level 2 pathways were “Cell motility,” “Cellular Processes and signaling,” “Genetic information processing,” “Lipid metabolism,” “Membrane transport,” “Metabolism,” “Signal transduction,” “Transcription,” and “Xenobiotics degradation.”

Plugging the data into the Bugbase-webtool revealed a significantly stimulating effect of sophorolipids and rhamnolipids on the formation of biofilms and mobile elements, stress tolerance and increased abundance of potential pathogens, Gram negative and facultative anaerobic bacteria all properties related to the Proteobacteria phylum. This coincides with our observation of an increased abundance of *Escherichia*/*Shigella* (Supplementary Data Sheet 2).

### Flagellin Levels

**Figure 8:**
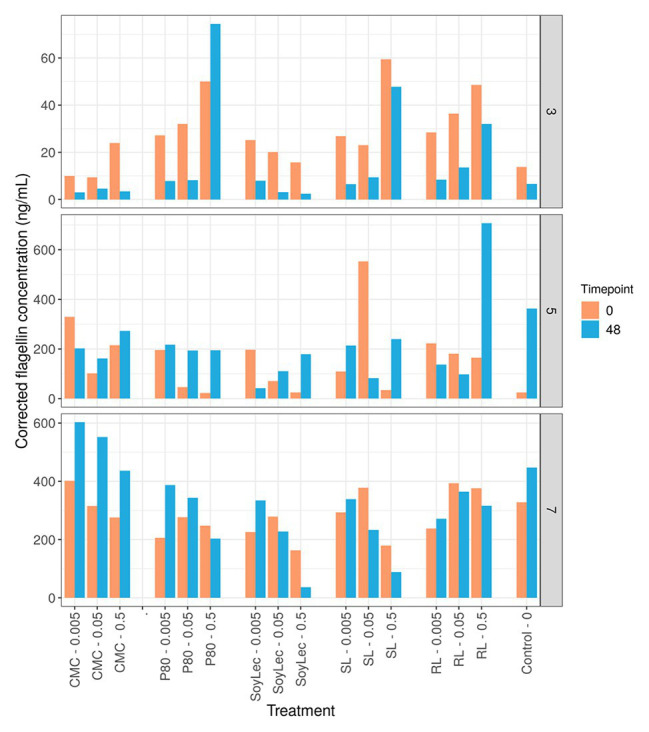
Flagellin concentrations obtained using mTLR5 HEK blue reporter cells for three donors at the start (T0) and the end (T2) of *in vitro* 48 h batch experiments of fecal material from 10 donors in sugar depleted medium supplemented with five emulsifiers at four concentrations. Donors for the flagellin assay were selected based on their low (D3), high (D7), and intermediate (D5) metabolic responses to the emulsifiers.

In order to validate the PICRUSt prediction of a higher motility potential, a HEK-blue mTLR5 reporter cell assay was used for the detection of bacterial flagellin. The response of the flagellin concentrations to the emulsifiers was found to be largely donor-dependent, and inconsistent shifts were observed in function of incubation time (Figure 8). Shifts in flagellin levels upon emulsifier dosage were variable, meaning that the prediction of higher motility by PICRUSt could not be substantiated.

#### Donor Diversity

For all endpoints, inter-individual variability was observed in response to the *in vitro* incubations and emulsifier treatments (Figures S4, S5, S7 in Supplementary Data Sheet 1). In terms of community structure, Figure 3A shows that each donor clusters separately. This clustering was found to be significant (*p*_dbRDA_ < 0.05).

To assess in more detail whether there was coherence in the read-outs with respect to donor susceptibility, we ranked the donors according to their response on the most relevant parameters, i.e., intact cell counts, production of the most important SCFA (acetate, propionate, and butyrate), and the absolute and relative abundance of *Escherichia*/*Shigella*.

**Figure 9:**
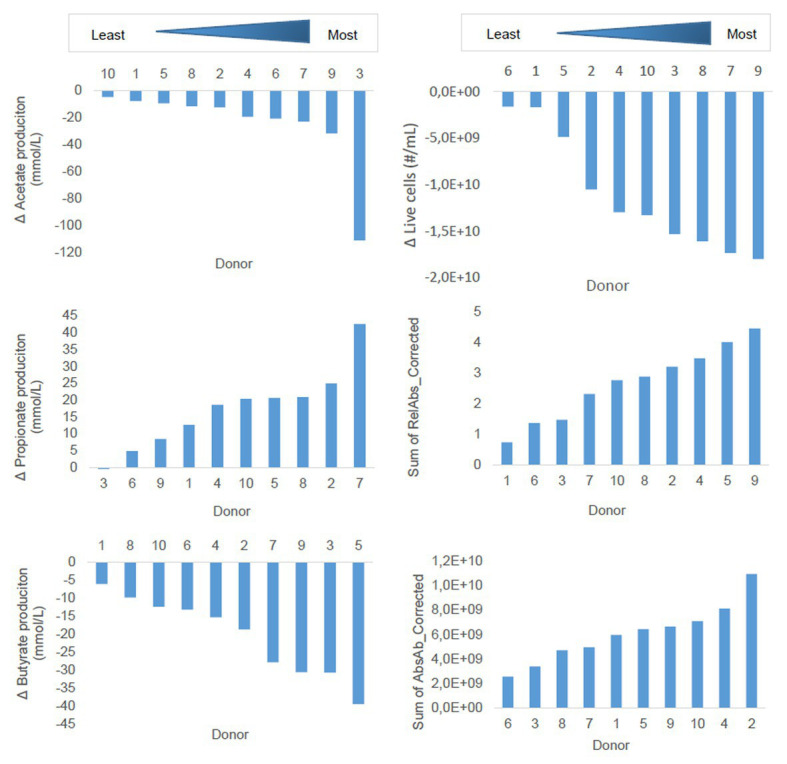
Ranking of donors of fecal material for 48 h batch incubations in sugar depleted medium supplemented with five emulsifiers at four concentrations. Donors were ranked along the principal parameters impacted by the emulsifiers. Measures were calculated based on cumulative (sum of all treatments) change in 48 h production of SCFA or cumulative 48 h change in living cell counts, relative or absolute abundances of *Escherichia*/*Shigella*.

We observed that the susceptibility of the donors to the effects of the emulsifiers depended on the targeted parameter (Figure 9). Some donors consistently ranked as highly susceptible (D9) or less susceptible (D6 and D1), while for other donors, ranking was more variable over the different parameters.

#### Equivalent Emulsifier Concentrations

We sought to compare the effects of biosurfactants versus those of conventional chemical emulsifiers. When comparing 0.5% (m/v) of chemical emulsifier with 0.05% (m/v) of biosurfactant – the concentrations most representative of the currently applied levels of dietary emulsifiers – the previously described effects of the biosurfactants (decreased levels of acetate, butyrate, intact and total cell concentration, and increased abundance of propionate and increased abundance of *Escherichia*/*Shigella*) were significant compared to the effects of the chemical emulsifiers (Table S4 in Supplementary Data Sheet 1). When comparing 0.05% (m/v) of chemical emulsifier with 0.005% (m/v) of biosurfactant, the effects were not significantly different, except for the effects of rhamnolipids on the cell count data.

## Discussion

We found dietary emulsifiers to significantly alter human gut microbiota toward a composition and functionality with potentially higher pro-inflammatory properties. While donor-dependent differences in microbiota response were observed, our *in vitro* experimental setup showed these effects to be primarily emulsifier-dependent. Rhamnolipids and sophorolipids had the strongest impact with a sharp decrease in intact cell counts, an increased abundance in potentially pathogenic genera-like *Escherichia*/*Shigella* and *Fusobacterium*, a decreased abundance of beneficial Bacteroidetes and *Barnesiella*, and a predicted increase in flagellar assembly and general motility. The latter was not substantiated through direct measurements, though. The effects were less pronounced for soy lecithin, while chemical emulsifiers P80 and CMC showed the smallest effects. Short chain fatty acid production, with butyrate production, in particular, was also affected by the respective emulsifiers, again in an emulsifier‐ and donor-dependent manner.

One of the most profound impacts of emulsifier treatment toward gut microbiota was the decline in intact microbial cell counts. The degree of microbiome elimination in this study seems comparable to what has been observed for antibiotic treatments (Francino, 2016; Guirro et al., 2019). Since antibiotics are considered detrimental for gut ecology, this may serve as a warning sign with respect to emulsifier usage. Emulsifiers also act as surfactants, which are known for their membrane solubilizing properties (Jones, 1999). The fact that the observed decline in microbial viability was dependent on emulsifier dose and on the emulsifying potential of the supplemented compound, as measured by the aqueous surface tension reduction (Table 1), leads us to conclude that the dietary emulsifiers attack the bacterial cells principally at the level of the cell membrane.

**Table 1:** Emulsifier characteristics of sodium carboxymethylcellulose, Span60, P80, phosphatidylcholine (major component of soy lecithin), sophorolipids, and rhamnolipids.

	MW (g/mol)	CMC^*^ (mg/L)	Source	Min. S.T.^**^ (mN/m)	Source
Carboxymethylcellulose	250,000^1^^,^^2^	-		-	
Sorbitan tristearate (Span60)	430.62^3^	21.53	Michor and Berg, 2015	62	Parreidt et al., 2018
P80	1,310^4^	0.012 mM =15.72 mg/L 0.033 mM =43.23 mg/L	Pogorzelski et al., 2012 Bąk and Podgórska, 2016	+/−43 36–38	Bąk and Podgórska, 2016 Szymczyk et al., 2018
Phophatidylcholine	758.1^5^ 643.9 g/mol^6^ 786.1^7^	0.998 PC (16:0/16:0) 0.001 PC (18:0/18:0) 13,600 (lecithin)	Zhang et al., 2017 Zhang et al., 2017 Wu and Wang, 2003	38 mN/m (PC) (21 mN/m for PI)	Wu and Wang, 2003 Wu and Wang, 2003
Sophorolipids	706^8^	150 27.17 10 20–130	Kim et al., 2005 Daverey and Pakshirajan, 2010 Otto et al., 1999 Ma et al., 2012	48 34.18 36 31–34.2 32.23–42.13	Kim et al., 2005 Daverey and Pakshirajan, 2010 Otto et al., 1999 Develter and Lauryssen, 2010 Ma et al., 2012
Rhamnolipids	650.79^9^	50–500 18.75 15–20 30	Li et al., 2019 Moussa et al., 2014 Hörmann et al., 2010 Vu et al., 2015	28 34 25–30 29.4 30–35	Li et al., 2019 Moussa et al., 2014 Van Bogaert, 2008 Hörmann et al., 2010 Vu et al., 2015

* Critical micelle concentration.

** Minimal surface tension in aqueous solution.

1 https://www.sigmaaldrich.com/catalog/product/aldrich/419311?lang=en&region=CA (4-06-2020).

2 https://pubchem.ncbi.nlm.nih.gov/compound/Carboxymethylcellulose-sodium (4-06-2020).

3 https://www.sigmaaldrich.com/catalog/product/sial/85546?lang=en&region=CA (4-06-2020).

4 https://www.sigmaaldrich.com/content/dam/sigma-aldrich/docs/Sigma-Aldrich/Product_Information_Sheet/p8074pis.pdf (4-06-2020).

5 https://pubchem.ncbi.nlm.nih.gov/compound/Soybean-lecithin (23-01-2020).

6 https://pubchem.ncbi.nlm.nih.gov/compound/57369748 (23-01-2020).

7 https://pubchem.ncbi.nlm.nih.gov/compound/6441487 (23-01-2020).

8 https://pubchem.ncbi.nlm.nih.gov/compound/Sophorolipid (23-01-2020).

9 https://pubchem.ncbi.nlm.nih.gov/compound/5458394 (23-01-2020).

We also found this antimicrobial effect from the tested emulsifiers to be highly selective, confirming previous observations by Moore (1997) who showed that the effects of surfactants are dependent on the bacterial species. We found the *Escherichia*/*Shigella* genus to be particularly resistant against the surfactants antimicrobial effects. This agrees with Kramer et al. (1984) and Nickerson and Aspedon (1992) who demonstrated the surfactant resistance of enteric bacteria *Enterobacter cloacae* and *E. coli* against sodium dodecyl sulfate. They showed that this resistance is widespread among *Enterobacteriaceae*, that it is energy-dependent, and that exposure to sodium dodecyl sulfate altered the expressions of 19 proteins, of which three were later tentatively identified as clpP, clpB, and clpX intracellular proteases (Rajagopal et al., 2002). Also membrane-derived oligosaccharides, present in the periplasm of Gram negative bacteria, were found to be essential to the detergent-resistant properties of *E. coli* (Rajagopal et al., 2003). Even though this could explain the increased abundance in this study for *Enterobacteriaceae* and *Escherichia*/*Shigella*, the mechanism by which other species are (un)affected by the emulsifiers is not known.

Interestingly, our prediction of altered functionality using PICRUSt analysis indicated a potential increase in levels of motility-genes, even though we could not confirm increased flagellin levels by direct measurement. Increased motility has been observed upon addition of emulsifiers before (Lock et al., 2018). This finding may again be related to the increased relative abundance of *Escherichia*/*Shigella*, since these are known flagella (gene)-bearers (Girón, 1995; Tominaga et al., 2001; Mittal et al., 2003). Increased flagellin levels constitute a potential health risk as flagellin is considered an important virulence factor; this particular microbe-associated molecular pattern may trigger inflammation upon binding to TLR5. A higher degree of flagellation would thus represent more motile bacteria and result in a gut microbiome that is able to more aggressively penetrate the mucus layer and subsequently reach gut epithelial cells (Ramos et al., 2004; Chassaing et al., 2015). Chassaing et al. (2015, 2017) demonstrated possible consequences for the host: an increased inflammatory response in both gut and body, contributing to increased adiposity and weight gain.

Effects toward microbial metabolic functionality, as measured by SCFA-levels, were emulsifier-dependent. Nevertheless, consistent shifts in SCFA-profiles were observed for all three emulsifiers for which significant alterations were visible, i.e., a decrease in butyrate and an increase in propionate production. For sophorolipids, the strong increase in propionate production can be related to the increased abundance of *Phascolarctobacterium*. This genus is known to produce propionate from succinate produced by *E. coli* cells (Del Dot et al., 1993). For rhamnolipids, we assume that similar cross-feeding interactions occurred, even though propionate levels remained stable. We hypothesized that an increased propionate production by the *Escherichia*/*Phascolarctobacterium* consortium is counteracted by the higher antimicrobial activities from the rhamnolipids at increasing concentrations. The fact that L-rhamnose is a well-known propionate precursor (Reichardt et al., 2014), and that rhamnolytic pathways have been observed in *E. coli* and other Gammaproteobacteria (Rodionova et al., 2013) further support this idea. The increase in acetate and propionate production observed with soy lecithin can be linked to the metabolism of glycerol (De Weirdt et al., 2010). This is in agreement with the upregulated abundances of the *Enterococcus* and *Clostridium* genera at higher levels of soy lecithin, since these genera are known to metabolize glycerol (Bradbeer, 1965; Bizzini et al., 2010) as well as choline (Martínez-del Campo et al., 2015). Also the increased abundance of the *Acidaminoccus* genus would correspond with the increased acetate production (Chang et al., 2010). These findings indicate that observed shifts in SCFA-production can be attributed to shifts in microbial composition.

Whether the altered SCFA-production and -levels are positive or negative for host health is ambiguous. On the one hand, decreased butyrate levels can be considered negative, since butyrate is known to protect the gut epithelium from inflammation and cancerous growth (Canani et al., 2011; Cani, 2017; Liu et al., 2018). On the other hand, propionate is also considered a health-promoting SCFA (Hosseini et al., 2011; Weitkunat et al., 2016; Louis and Flint, 2017). Propionate production is related to decreased lipogenesis in the liver and is supposed to enhance satiety mechanisms, which would lower the chance of developing obesity (Hosseini et al., 2011; Weitkunat et al., 2016). It is, however, questionable whether such benefit may predominate the purported negative effects from the observed antimicrobial effects and the increase in pro-inflammatory markers, such as flagellated microbiota and a drop in butyrate production.

Another point relating to health effects entails that rhamnolipids, sophorolipids, and – to a lesser extent – soy lecithin significantly decreased diversity parameters. Decreased microbiome diversity frequently occurs with compromised health conditions such as obesity, insulin resistance, dyslipidemia (Turnbaugh et al., 2009; Le Chatelier et al., 2013), Type 1 diabetes (Patterson et al., 2016), heart failure (Luedde et al., 2017), and inflammatory bowel disease (Arumugam et al., 2011; Chassaing et al., 2015). The increased prevalence of the *Escherichia*/*Shigella* genus has also been observed with multiple metabolic conditions (Lupp et al., 2007; Lecomte et al., 2015; Shin et al., 2015). Increased *Enterobacteriaceae* abundance was previously linked with an increased gut permeability (Pedersen et al., 2018), the consumption of high fat diets (Fei and Zhao, 2013; Lecomte et al., 2015; He et al., 2018), colitis (Lupp et al., 2007), cardiovascular disease (Jie et al., 2017), diabetes (Allin et al., 2015; Deschasaux et al., 2019), and even undernourishment and iron deficiency anemia (Shin et al., 2015; Muleviciene et al., 2018). With respect to soy lecithin, metabolism of phosphatidylcholine by the gut microbiota has been linked to cardiovascular disease (Wang et al., 2011; Tang and Hazen, 2014). We also found an increased abundance of the genus *Sutterella*, a bacterium that has been linked to autism spectrum disorders (Wang et al., 2013) and gut inflammation, notably by IgA-degradation (Moon et al., 2015; Kaakoush, 2020). Overall, we can, thus, conclude that exposure of the gut microbiota to dietary emulsifiers may result in profound compositional shifts, previously related to adverse health outcomes.

An important consideration is whether the observed *in vitro* effects would also take place in an *in vivo* setting. This will depend on a number of diet‐ and host-related factors. First, we found that the observed effects were concentration‐ and emulsifier-dependent. Choosing an emulsifier-concentration combination that minimizes adverse microbial impacts, without compromising food technical properties, could thus be a strategy to mitigate the harmful effects of food emulsifiers. Second, emulsifier concentration will continuously alter during gastrointestinal digestion, but the impact of the dilution with accompanying food products, excretion of digestive fluids, or absorption of water from the gut lumen on the final concentration reaching the gut microbiota has so far not yet been studied. Third, digestion by human enzymes will alter chemical structure of the emulsifiers. While CMC is resistant to breakdown by human digestive enzymes (Joint FAO/WHO Expert Committee on Food Additives, 1973), P80 and soy lecithin can be hydrolyzed by pancreatic lipases. For P80, only the polyethylene-sorbitan unit may reach the colon (Aguilar et al., 2015). Soy lecithin is mostly absorbed as lysolecithin and free fatty acids, but its detection in faces indicates that some fraction reaches the colon (Mortensen et al., 2017), where the choline and glycerol moieties are metabolized by the gut microbiota (Tang and Hazen, 2014). For both of these compounds, it will thus be necessary to verify if and to what extent the observed effects occur *in vivo* as well. With respect to rhamnolipids and sophorolipids, no literature is currently available on their digestion by human enzymes. Preliminary data from our side, however, have indicated only one alteration, a deacetylation of the sophorose units of the sophorolipids (Supplementary Data Sheet 3). These compounds may thus readily reach the colon and interact with the endogenous microbiota. Fourth, other dietary constituents in the gut, primarily lipids, and bile salts, will interact with emulsifiers (Naso et al., 2019). The mucus layer overlying the gut epithelium and the pH-fluctuations throughout the gut are other elements that may affect emulsifier-microbiota interactions. Finally, the total levels of emulsifiers consumed by an individual will determine both acute and chronic effects.

A last important element in the putative health impact from dietary emulsifiers concern’s interindividual variability. An individual’s unique microbiota and metabolism are important determinants of the potential health effects dietary emulsifiers could cause. While the overall effects from the different emulsifiers toward microbiota composition and functionality were quite consistent in our study, important interindividual differences in susceptibility of the microbiota were noted. Understanding what underlying factors and determinants drive this interindividual variability will be crucial to future health risk assessment of novel and existing dietary emulsifiers.

Food additives have come under scrutiny with respect to their impact on human health. Additives like colorants, artificial sweeteners, nitrites (NaNO_2_), and high fructose corn syrup have been associated with hyperactivity, cancer development, gastric cancer, and obesity, respectively (Arnold et al., 2012; Bryan et al., 2012; Payne et al., 2012; Carocho et al., 2014). In answer to these public concerns, a clean label movement has developed in the food industry that aims to provide food products with a more natural image. In this light, we investigated whether rhamnolipids and sophorolipids, two biotechnological emulsifiers, would yield less of an impact on the gut microbiota than the mainstream chemical emulsifiers, CMC and P80. Our results showed however, that rhamnolipids and sophorolipids, of all emulsifiers in this study, had the strongest impact on microbiota composition and functionality, even when equivalent concentrations were taken into account. Further analysis of our data showed that the observed effects toward the microbiota can potentially be linked to their higher emulsifying potential. All of this indicates that rhamnolipids and sophorolipids are probably no appropriate alternatives to conventional emulsifiers unless they are used at substantially lower concentrations. We note, however, that the model used in this study has impacted the microbiota as well. More research must thus point out whether the effects prevail in different models, as wel as *in vivo* and if so, whether concentrations can be kept low enough to avoid alleged adverse health effects.

## Data Availability Statement

The datasets presented in this study can be found in online repositories. The names of the repository/repositories and accession number(s) can be found in the article/Supplementary Material.

## Ethics Statement

The studies involving human participants were reviewed and approved by Ghent University Hospital (registration number BE670201836318). Written informed consent for participation was not required for this study in accordance with the national legislation and the institutional requirements.

## Author Contributions

This research was performed under promotorship of TW, JC, and AR. LM was responsible for the execution of all experiments, data visualization, and statistical analysis, as well as the assembly and submission of the manuscript. CC assisted in the execution of the PICRUSt algorithm. DESeq analysis was performed in collaboration with KP. Library preparation and 16S rRNA gene amplicon sequencing was performed by LR, our collaborator within JR group. All authors contributed to the article and approved the submitted version.

### Conflict of Interest

The authors declare that the research was conducted in the absence of any commercial or financial relationships that could be construed as a potential conflict of interest.

## References

[ref1] AAT Bioquest (2019). Quest Graph™ Four Parameter Logistic (4PL) Curve Calculator. Available at: https://www.aatbio.com/tools/four-parameter-logistic-4pl-curve-regression-online-calculator (Accessed March 13, 2020).

[ref2] Adams W. Bas H. Boutte T. Bueschelberger H. -G. Cooper J. M. Cotrell T. (2004). “Emulsifiers in food technology” in Emulsifiers in food technology. ed. Whitehurst R. J. (John Wiley and Sons Ltd.), 104.

[ref3] Aguilar F. Crebelli R. di Domenico A. Dusemund B. Frutos M. J. Galtier P. (2015). Scientific Opinion on the re-evaluation of polyoxyethylene sorbitan monolaurate (E 432), polyoxyethylene sorbitan monooleate (E 433), polyoxyethylene sorbitan monopalmitate (E 434), polyoxyethylene sorbitan monostearate (E 435) and polyoxyethylene sorbita. EFSA J. 13:4152. 10.2903/j.efsa.2015.4152 PMC13151592 42109790

[ref4] Allin K. H. Nielsen T. Pedersen O. (2015). Mechanisms in endocrinology: gut microbiota in patients with type 2 diabetes mellitus. Eur. J. Endocrinol. 172, R167–R177. 10.1530/EJE-14-0874, PMID: 25416725

[ref5] Anderson J. R. Carroll I. Azcarate-Peril M. A. Rochette A. D. Heinberg L. J. Peat C.. (2017). A preliminary examination of gut microbiota, sleep, and cognitive flexibility in healthy older adults. Sleep Med. 38, 104–107. 10.1016/j.sleep.2017.07.018, PMID: 29031742 PMC7433257

[ref6] Arnold L. E. Lofthouse N. Hurt E. (2012). Artificial food colors and attention-deficit/hyperactivity symptoms: conclusions to dye for. Neurotherapeutics 9, 599–609. 10.1007/s13311-012-0133-x, PMID: 22864801 PMC3441937

[ref7] Arumugam M. Raes J. Pelletier E. Le Paslier D. Yamada T. Mende D.. (2011). Enterotypes of the human gut microbiome. Nature 473, 174–180. 10.1038/nature09944, PMID: 21508958 PMC3728647

[ref8] Asioli D. Aschemann-Witzel J. Caputo V. Vecchio R. Annunziata A. Næs T.. (2017). Making sense of the “clean label” trends: a review of consumer food choice behavior and discussion of industry implications. Food Res. Int. 99, 58–71. 10.1016/j.foodres.2017.07.022, PMID: 28784520

[ref9] Bąk A. Podgórska W. (2016). Interfacial and surface tensions of toluene/water and air/water systems with nonionic surfactants Tween 20 and Tween 80. Colloids Surf. A Physicochem. Eng. Asp. 504, 414–425. 10.1016/j.colsurfa.2016.05.091

[ref10] Bischoff S. C. (2011). “Gut health”: a new objective in medicine? BMC Med. 9:24. 10.1186/1741-7015-9-24, PMID: 21401922 PMC3065426

[ref11] Biswal D. R. Singh R. P. (2004). Characterisation of carboxymethyl cellulose and polyacrylamide graft copolymer. Carbohydr. Polym. 57, 379–387. 10.1016/j.carbpol.2004.04.020

[ref12] Bizzini A. Zhao C. Budin-Verneuil A. Sauvageot N. Giard J. C. Auffray Y.. (2010). Glycerol is metabolized in a complex and strain-dependent manner in *Enterococcus faecalis*. J. Bacteriol. 192, 779–785. 10.1128/JB.00959-09, PMID: 19966010 PMC2812454

[ref13] Bradbeer C. (1965). The Clostridial fermentations of choline ethanolamine. J. Bacteriol. Chem. 240, 4669–4674. 5846987

[ref14] Branca F. Lartey A. Oenema S. Aguayo V. Stordalen G. A. Richardson R.. (2019). Transforming the food system to fight non-communicable diseases. BMJ 365:l296. 10.1136/bmj.l296, PMID: 30692128 PMC6349221

[ref15] Broussard J. L. Devkota S. (2016). The changing microbial landscape of Western society: diet, dwellings and discordance. Mol. Metab. 5, 737–742. 10.1016/j.molmet.2016.07.007, PMID: 27617196 PMC5004226

[ref16] Bryan N. S. Alexander D. D. Coughlin J. R. Milkowski A. L. Boffetta P. (2012). Ingested nitrate and nitrite and stomach cancer risk: an updated review. Food Chem. Toxicol. 50, 3646–3665. 10.1016/j.fct.2012.07.062, PMID: 22889895

[ref17] Buysschaert B. Kerckhof F. M. Vandamme P. De Baets B. Boon N. (2018). Flow cytometric fingerprinting for microbial strain discrimination and physiological characterization. Cytometry A 93, 201–212. 10.1002/cyto.a.23302, PMID: 29266796

[ref18] Cameotra S. S. Makkar R. S. (2004). Recent applications of biosurfactants as biological and immunological molecules. Curr. Opin. Microbiol. 7, 262–266. 10.1016/j.mib.2004.04.006, PMID: 15196493

[ref19] Canani R. B. di Costanzo M. Leone L. Pedata M. Meli R. Calignano A. (2011). Potential beneficial effects of butyrate in intestinal and extraintestinal diseases. World J. Gastroenterol. 17, 1519–1528. 10.3748/wjg.v17.i12.1519, PMID: 21472114 PMC3070119

[ref20] Cani P. D. (2017). Gut cell metabolism shapes the microbiome. Microbiome 357, 548–549. 10.1126/science.aao2202, PMID: 28798116

[ref21] Carocho M. Barreiro M. F. Morales P. Ferreira I. C. F. R. (2014). Adding molecules to food, pros and cons: a review on synthetic and natural food additives. Compr. Rev. Food Sci. Food Saf. 13, 377–399. 10.1111/1541-4337.12065 33412697

[ref22] Chang Y. J. Pukall R. Saunders E. Lapidus A. Copeland A. Nolan M.. (2010). Complete genome sequence of acidaminococcus fermentans type strain (VR_4_). Stand. Genomic Sci. 3, 1–14. 10.4056/sigs.1002553, PMID: 21304687 PMC3035267

[ref23] Chassaing B. Koren O. Goodrich J. K. Poole A. C. Srinivasan S. Ley R. E.. (2015). Dietary emulsifiers impact the mouse gut microbiota promoting colitis and metabolic syndrome. Nature 519, 92–96. 10.1038/nature14232, PMID: 25731162 PMC4910713

[ref600] Chassaing B. Van De Wiele T. De Bodt J. Marzorati M. Gewirtz A. T. (2017). Dietary emulsifiers directly alter human microbiota composition and gene expression ex vivo potentiating intestinal inflammation. Gut 66, 1414–1427. 10.1136/gutjnl-2016-313099 28325746 PMC5940336

[ref24] Costa C. S. Del-ponte B. Cecília M. Assunção F. Santos I. S. (2017). Consumption of ultra-processed foods and body fat during childhood and adolescence: a systematic review. 21, 148–159. 10.1017/S1368980017001331, PMC10260745 28676132

[ref25] Daverey A. Pakshirajan K. (2010). Sophorolipids from Candida bombicola using mixed hydrophilic substrates: production, purification and characterization. Colloids Surf. B: Biointerfaces 79, 246–253. 10.1016/j.colsurfb.2010.04.002, PMID: 20427162

[ref26] De Boever P. Deplancke B. Verstraete W. (2000). Fermentation by gut microbiota cultured in a simulator of the human intestinal microbial ecosystem is improved by supplementing a soygerm powder. J. Nutr. 130, 2599–2606. 10.1093/jn/130.10.2599, PMID: 11015496

[ref30] Del Dot T. Osawa R. Stackebrandt E. (1993). Phascolarctobacterium faecium gen. nov, spec. nov., a novel taxon of the sporomusa group of bacteria. Syst. Appl. Microbiol. 16, 380–384. 10.1016/S0723-2020(11)80269-9

[ref27] De Paepe K. Kerckhof F. M. Verspreet J. Courtin C. M. Van de Wiele T. (2017). Inter-individual differences determine the outcome of wheat bran colonization by the human gut microbiome. Environ. Microbiol. 19, 3251–3267. 10.1111/1462-2920.13819, PMID: 28618173

[ref28] De Paepe K. Verspreet J. Verbeke K. Raes J. Courtin C. M. Van de Wiele T. (2018). Introducing insoluble wheat bran as a gut microbiota niche in an in vitro dynamic gut model stimulates propionate and butyrate production and induces colon region specific shifts in the luminal and mucosal microbial community. Environ. Microbiol. 20, 3406–3426. 10.1111/1462-2920.14381, PMID: 30126070

[ref31] Deschasaux M. Bouter K. Prodan A. Levin E. Groen A. Herrema H. (2019). Differences in gut microbiota composition in metabolic syndrome and type 2 diabetes subjects in a multi-ethnic population: the HELIUS study. Available at: https://app.oxfordabstracts.com/events/696/program-app/submission/126560 (Accessed July 1, 2020).

[ref32] Develter D. W. G. Lauryssen L. M. L. (2010). Properties and industrial applications of sophorolipids. Eur. J. Lipid Sci. Technol. 112, 628–638. 10.1002/ejlt.200900153

[ref29] De Weirdt R. Possemiers S. Vermeulen G. Moerdijk-Poortvliet T. C. W. Boschker H. T. S. Verstraete W.. (2010). Human faecal microbiota display variable patterns of glycerol metabolism. FEMS Microbiol. Ecol. 74, 601–611. 10.1111/j.1574-6941.2010.00974.x, PMID: 20946352

[ref33] Diether N. E. Willing B. P. (2019). Microbial fermentation of dietary protein: an important factor in diet-microbe-host interaction. Microorganisms 7:19. 10.3390/microorganisms7010019, PMID: 30642098 PMC6352118

[ref34] Ding R. X. Goh W. R. Wu R. N. Yue X. Q. Luo X. Khine W. W. T.. (2019). Revisit gut microbiota and its impact on human health and disease. J. Food Drug Anal. 27, 623–631. 10.1016/j.jfda.2018.12.012, PMID: 31324279 PMC9307029

[ref35] EFSA (2019). Europeans on today’s food issues: new EU-wide survey comes out on first World Food Safety Day. Available at: https://www.efsa.europa.eu/en/press/news/190607 (Accessed February 23, 2020).

[ref36] Ehehalt R. Braun A. Karner M. Füllekrug J. Stremmel W. (2010). Phosphatidylcholine as a constituent in the colonic mucosal barrier--physiological and clinical relevance. Biochim. Biophys. Acta 1801, 983–993. 10.1016/j.bbalip.2010.05.014, PMID: 20595010

[ref39] European Commision (2011). 00172-Polysorbates-E433. Available at: https://webgate.ec.europa.eu/foods_system/main/index.cfm?event=substance.view&identifier=172 (Accessed August 6, 2019).

[ref37] European Commision (2014). 00191-Sodium carboxymethyl cellulose-E466. Available at: https://webgate.ec.europa.eu/foods_system/main/index.cfm?event=substance.view&identifier=191 (Accessed July 8, 2020).

[ref38] European Commision (2018). 00115-Soy lecithin-E322. Available at: https://webgate.ec.europa.eu/foods_system/main/index.cfm?event=substance.view&identifier=115 (Accessed July 5, 2020).

[ref40] Falcioni T. Papa S. Gasol J. M. (2008). Evaluating the flow-cytometric nucleic acid double-staining protocol in realistic situations of planktonic bacterial death. Appl. Environ. Microbiol. 74, 1767–1779. 10.1128/AEM.01668-07, PMID: 18223113 PMC2268295

[ref41] FDA (2019a). CFR-Code of Federal Regulations Title 21-polysorbate 80. Available at: https://www.accessdata.fda.gov/scripts/cdrh/cfdocs/cfCFR/CFRSearch.cfm?fr=172.840 (Accessed July 5, 2020).

[ref42] FDA (2019b). CFR-Code of Federal Regulations Title 21-sodium carboxymethyl cellulose. Available at: https://www.accessdata.fda.gov/scripts/cdrh/cfdocs/cfCFR/CFRSearch.cfm?fr=182.1745 (Accessed July 8, 2020).

[ref43] FDA (2019c). CFR-Code of Federal Regulations Title 21-Soy lecithin. Available at: https://www.accessdata.fda.gov/scripts/cdrh/cfdocs/cfCFR/CFRSearch.cfm?fr=184.1400 (Accessed July 6, 2020).

[ref44] FDA (2020). CARBOXYMETHYL CELLULOSE, SODIUM SALT. Available at: https://www.accessdata.fda.gov/scripts/fdcc/?set=FoodSubstances&id=CARBOXYMETHYLCELLULOSESODIUMSALT&sort=Sortterm&order=ASC&startrow=1&type=basic&search=carboxymethyl (Accessed July 5, 2020).

[ref45] Fei N. Zhao L. (2013). An opportunistic pathogen isolated from the gut of an obese human causes obesity in germfree mice. ISME J. 7, 880–884. 10.1038/ismej.2012, PMID: 23235292 PMC3603399

[ref46] FMI (2020). Polysorbate-80 Market: Global Industry Analysis and Opportunity Assessment 2016–2026. Available at: https://www.futuremarketinsights.com/reports/polysorbate-80-market

[ref47] Francino M. P. (2016). Antibiotics and the human gut microbiome: dysbioses and accumulation of resistances. Front. Microbiol. 6:1543. 10.3389/fmicb.2015.01543, PMID: 26793178 PMC4709861

[ref48] Girón J. A. (1995). Expression of flagella and motility by Shigella. Mol. Biol. 18, 63–75. 10.1111/j.1365-2958.1995.mmi_18010063.x, PMID: 8596461

[ref49] Guirro M. Costa A. Gual-Grau A. Herrero P. Torrell H. Canela N.. (2019). Effects from diet-induced gut microbiota dysbiosis and obesity can be ameliorated by fecal microbiota transplantation: a multiomics approach. PLoS One 14:e0218143. 10.1371/journal.pone.0218143, PMID: 31545802 PMC6756520

[ref50] Haba E. Pinazo A. Jauregui O. Espuny M. J. Infante M. R. Manresa A. (2003). Physicochemical characterization and antimicrobial properties of rhamnolipids produced by *Pseudomonas aeruginosa* 47T2 NCBIM 40044. Biotechnol. Bioeng. 81, 316–322. 10.1002/bit.10474, PMID: 12474254

[ref51] He C. Cheng D. Peng C. Li Y. Zhu Y. Lu N. (2018). High-fat diet induces dysbiosis of gastric microbiota prior to gut microbiota in association with metabolic disorders in mice. Front. Microbiol. 9:639. 10.3389/fmicb.2018.00639, PMID: 29686654 PMC5900050

[ref52] Hercules Inc., and Aqualon (1999). Aqualon-sodium carboxymethyl cellulose-physical and chemical propertie. Available at: https://www.academia.edu/8443565/Physical_and_Chemical_Properties_AQUALON_Sodium_Carboxymethylcellulose (Accessed May 27, 2020).

[ref53] Hörmann B. Müller M. M. Syldatk C. Hausmann R. (2010). Rhamnolipid production by Burkholderia plantarii DSM 9509T. Eur. J. Lipid Sci. Technol. 112, 674–680. 10.1002/ejlt.201000030

[ref54] Hosseini E. Grootaert C. Verstraete W. Van de Wiele T. (2011). Propionate as a health-promoting microbial metabolite in the human gut. Nutr. Rev. 69, 245–258. 10.1111/j.1753-4887.2011.00388.x, PMID: 21521227

[ref55] Jiang Z. Zhao M. Zhang H. Li Y. Liu M. Feng F. (2018). Antimicrobial emulsifier-glycerol monolaurate induces metabolic syndrome, gut microbiota dysbiosis, and systemic low-grade inflammation in low-fat diet fed mice. Mol. Nutr. Food Res. 62, 1–11. 10.1002/mnfr.201700547, PMID: 29131494

[ref56] Jie Z. Xia H. Zhong S. L. Feng Q. Li S. Liang S.. (2017). The gut microbiome in atherosclerotic cardiovascular disease. Nat. Commun. 8:845. 10.1038/s41467-017-00900-1, PMID: 29018189 PMC5635030

[ref57] Joint FAO/WHO Expert Committee on Food Additives (1973). Toxicological evaluation of some food additives including anticaking agents, antimicrobials, antioxidants, emulsifiers and thickening agents. In IPCS Inchem. Available at: http://www.inchem.org/documents/jecfa/jecmono/v05je54.htm 4459150

[ref58] Jones M. N. (1999). Surfactants in membrane solubilisation. Int. J. Pharm. 177, 137–159. 10.1016/s0378-5173(98)00345-7, PMID: 10205610

[ref59] Kaakoush N. O. (2020). Sutterella species, IgA-degrading bacteria in ulcerative colitis. Trends Microbiol. 28, 519–522. 10.1016/j.tim.2020.02.018, PMID: 32544438

[ref60] Kanehisa M. Goto S. Sato Y. Furumichi M. Tanabe M. (2012). KEGG for integration and interpretation of large-scale molecular data sets. Nucleic Acids Res. 40, 109–114. 10.1093/nar/gkr988, PMID: 22080510 PMC3245020

[ref61] Kim H. S. Kim Y. B. Lee B. S. Kim E. K. (2005). Sophorolipid production by Candida bombicola ATCC 22214 from a corn-oil processing byproduct. J. Microbiol. Biotechnol. 15, 55–58.

[ref62] Kozich J. J. Westcott S. L. Baxter N. T. Highlander S. K. Schloss P. D. (2013). Development of a dual-index sequencing strategy and curation pipeline for analyzing amplicon sequence data on the miseq illumina sequencing platform. Appl. Environ. Microbiol. 79, 5112–5120. 10.1128/AEM.01043-13, PMID: 23793624 PMC3753973

[ref63] Kramer V. Nickerson K. W. Hamlett N. V. O’Hara C. (1984). Prevalence of extreme detergent resistance among the Enterobacteriaceae. Can. J. Microbiol. 30, 711–713. 10.1139/m84-106, PMID: 6744128

[ref64] Langille M. G. I. Zaneveld J. Caporaso J. G. McDonald D. Knights D. Reyes J. A.. (2013). Predictive functional profiling of microbial communities using 16S rRNA marker gene sequences. Nat. Biotechnol. 31, 814–821. 10.1038/nbt.2676, PMID: 23975157 PMC3819121

[ref65] Le Chatelier E. Nielsen T. Qin J. Prifti E. Hildebrand F. Falony G.. (2013). Richness of human gut microbiome correlates with metabolic markers. Nature 500, 541–546. 10.1038/nature12506, PMID: 23985870

[ref66] Lecomte V. Kaakoush N. O. Maloney C. A. Raipuria M. Huinao K. D. Mitchell H. M.. (2015). Changes in gut microbiota in rats fed a high fat diet correlate with obesity-associated metabolic parameters. PLoS One 10:e0126931. 10.1371/journal.pone.0126931, PMID: 25992554 PMC4436290

[ref67] Li Z. Zhang Y. Lin J. Wang W. Li S. (2019). High-yield di-rhamnolipid production by *Pseudomonas aeruginosa* YM4 and its potential application in MEOR. Molecules 24:1433. 10.3390/molecules24071433, PMID: 30979013 PMC6480329

[ref68] Liu H. Wang J. He T. Becker S. Zhang G. Li D.. (2018). Butyrate: a double-edged sword for health? Adv. Nutr. 9, 21–29. 10.1093/advances/nmx009, PMID: 29438462 PMC6333934

[ref69] Lock J. Y. Carlson T. L. Wang C. M. Chen A. Carrier R. L. (2018). Acute exposure to commonly ingested emulsifiers alters intestinal mucus structure and transport properties. Sci. Rep. 8:10008. 10.1038/s41598-018-27957-2, PMID: 29968743 PMC6030187

[ref70] Louis P. Flint H. J. (2017). Formation of propionate and butyrate by the human colonic microbiota. Environ. Microbiol. 19, 29–41. 10.1111/1462-2920.13589, PMID: 27928878

[ref71] Love M. I. Huber W. Anders S. (2014). Moderated estimation of fold change and dispersion for RNA-seq data with DESeq2. Genome Biol. 15:550. 10.1186/s13059-014-0550-8, PMID: 25516281 PMC4302049

[ref72] Luedde M. Winkler T. Heinsen F. A. Rühlemann M. C. Spehlmann M. E. Bajrovic A.. (2017). Heart failure is associated with depletion of core intestinal microbiota. ESC Heart Fail. 4, 282–290. 10.1002/ehf2.12155, PMID: 28772054 PMC5542738

[ref73] Lupp C. Robertson M. L. Wickham M. E. Sekirov I. Champion O. L. Gaynor E. C.. (2007). Host-mediated inflammation disrupts the intestinal microbiota and promotes the overgrowth of Enterobacteriaceae. Cell Host Microbe 2, 119–129. 10.1016/j.chom.2007.06.010, PMID: 18005726

[ref74] Ma X. Li H. Song X. (2012). Surface and biological activity of sophorolipid molecules produced by Wickerhamiella domercqiae var. sophorolipid CGMCC 1576. J. Colloid Interface Sci. 376, 165–172. 10.1016/j.jcis.2012.03.007, PMID: 22459028

[ref75] Mallet C. P. (ed.) (1992). Frozen food technology (Blackie Academic and Professional, an imprint of Chapman and Hall).

[ref77] Martínez-del Campo A. Bodea S. Hamer H. A. Marks J. A. Haiser H. J. Turnbaugh P. J.. (2015). Characterization and detection of a widely distributed gene cluster that predicts anaerobic choline utilization by human gut bacteria. mBio 6, e00042–e00115. 10.1128/mBio.00042-15, PMID: 25873372 PMC4453576

[ref76] Martínez Steele E. Popkin B. M. Swinburn B. Monteiro C. A. (2017). The share of ultra-processed foods and the overall nutritional quality of diets in the US: evidence from a nationally representative cross-sectional study. Popul. Health Metrics 15:6. 10.1186/s12963-017-0119-3, PMID: 28193285 PMC5307821

[ref78] McMurdie P. J. Holmes S. (2014). Waste not, want not: why rarefying microbiome data is inadmissible. PLoS Comput. Biol. 10:e1003531. 10.1371/journal.pcbi.1003531, PMID: 24699258 PMC3974642

[ref79] Michor E. L. Berg J. C. (2015). Temperature effects on micelle formation and particle charging with span surfactants in apolar media. Langmuir 31, 9602–9607. 10.1021/acs.langmuir.5b02711, PMID: 26301921

[ref80] Miclotte L. Van de Wiele T. (2019). Food processing, gut microbiota and the globesity problem. Crit. Rev. Food Sci. Nutr. 60, 1769–1782. 10.1080/10408398.2019.1596878, PMID: 30945554

[ref81] Mittal N. Budrene E. O. Brenner M. P. Van Oudenaarden A. (2003). Motility of *Escherichia coli* cells in clusters formed by chemotactic aggregation. Proc. Natl. Acad. Sci. U. S. A. 100, 13259–13263. 10.1073/pnas.2233626100, PMID: 14597724 PMC263772

[ref82] Monteiro C. A. Cannon G. Moubarac J. Levy R. B. Louzada M. L. C. Jaime P. C. (2017). Commentary the UN decade of nutrition, the NOVA food classi fi cation and the trouble with ultra-processing. Public Health Nutr. 21, 5–17. 10.1017/S1368980017000234, PMID: 28322183 PMC10261019

[ref83] Monteiro C. A. Moubarac J. -C. Cannon G. Ng S. W. Popkin B. (2013). Ultra-processed products are becoming dominant in the global food system. Obes. Rev. 14, 21–28. 10.1111/obr.12107, PMID: 24102801

[ref84] Moon C. Baldridge M. T. Wallace M. A. Burnham C. A. D. Virgin H. W. Stappenbeck T. S. (2015). Vertically transmitted faecal IgA levels determine extra-chromosomal phenotypic variation. Nature 521, 90–93. 10.1038/nature14139, PMID: 25686606 PMC4425643

[ref85] Moore S. L. (1997). The Mechanisms of Antibacterial Action of Some Nonionic Surfactants. University of Brighton in collaboration with Unilever Research.

[ref86] Mortensen A. Aguilar F. Crebelli R. Di Domenico A. Frutos M. J. Galtier P.. (2017). Re-evaluation of lecithins (E 322) as a food additive. EFSA J. 15:e04742. 10.2903/j.efsa.2017.4742, PMID: 32625454 PMC7010002

[ref87] Mourad A. M. De Carvalho Pincinato E. Mazzola P. G. Sabha M. Moriel P. (2010). Influence of soy lecithin administration on hypercholesterolemia. Cholesterol 2010:824813. 10.1155/2010/824813, PMID: 21490917 PMC3065734

[ref88] Moussa T. A. A. Mohamed M. S. Samak N. (2014). Production and characterization of di-rhamnolipid produced by *Pseudomonas aeruginosa* TMN. Braz. J. Chem. Eng. 31, 867–880. 10.1590/0104-6632.20140314s00002473

[ref89] Msagati T. A. (2012). “Chemistry of food additives and preservatives” in Chemistry of food additives and preservatives. Wiley-Blackwell, 33–62.

[ref90] Mu C. Yang Y. Luo Z. Guan L. Zhu W. (2016). The colonic microbiome and epithelial transcriptome are altered in rats fed a high-protein diet compared with a normal-protein diet. J. Nutr. 146, 474–483. 10.3945/jn.115.223990, PMID: 26843585

[ref91] Muleviciene A. D’Amico F. Turroni S. Candela M. Jankauskiene A. (2018). Iron deficiency anemia-related gut microbiota dysbiosis in infants and young children: a pilot study. Acta Microbiol. Immunol. Hung. 65, 551–564. 10.1556/030.65.2018.045, PMID: 30418043

[ref92] Musso G. Gambino R. Cassader M. (2010). Obesity, diabetes, and gut microbiota: the hygiene hypothesis expanded? Diabetes Care 33, 2277–2284. 10.2337/dc10-0556, PMID: 20876708 PMC2945175

[ref93] Naso J. N. Bellesi F. A. Ruiz-Henestrosa V. M. R. Pilosof A. M. R. (2019). Studies on the interactions between bile salts and food emulsifiers under in vitro duodenal digestion conditions to evaluate their bile salt binding potential. Colloids Surf. B: Biointerfaces 174, 493–500. 10.1016/j.colsurfb.2018.11.024, PMID: 30497011

[ref94] Nickerson K. W. Aspedon A. (1992). Detergent-shock response in enteric bacteria. Mol. Biol. 6, 957–961. 10.1111/j.1365-2958.1992.tb02161.x, PMID: 1316532

[ref95] Nielsen C. K. Kjems J. Mygind T. Snabe T. Meyer R. L. (2016). Effects of tween 80 on growth and biofilm formation in laboratory media. Front. Microbiol. 7:1878. 10.3389/fmicb.2016.01878, PMID: 27920774 PMC5118432

[ref96] Nitschke M. Silva S. S. E. (2018). Recent food applications of microbial surfactants. Crit. Rev. Food Sci. Nutr. 58, 631–638. 10.1080/10408398.2016.1208635, PMID: 27437564

[ref97] Oksanen J. F. Blanchet G. Friendly M. Kindt R. Legendre P. McGlinn D. (2019). Vegan: community ecology package. R package version 2.5-6. Available at: https://cran.r-project.org/web/packages/vegan/index.html

[ref98] Otto R. T. Daniel H. -J. Pekin G. Müller-Decker K. Fürstenberger G. Reuss M. (1999). Production of sophorolipids from whey. Appl. Microbiol. Biotechnol. 52, 495–501. 10.1007/s002530051551 10570796

[ref99] Parreidt T. S. Schott M. Schmid M. Müller K. (2018). Effect of presence and concentration of plasticizers, vegetable oils, and surfactants on the properties of sodium-alginate-based edible coatings. Int. J. Mol. Sci. 19:742. 10.3390/ijms19030742, PMID: 29509669 PMC5877603

[ref100] Patterson E. E. Ryan P. M. Cryan J. F. Dinan T. G. Paul Ross R. Fitzgerald G. F.. (2016). Gut microbiota, obesity and diabetes. Postgrad. Med. J. 92, 286–300. 10.1136/postgradmedj-2015-133285, PMID: 26912499

[ref101] Payne A. N. Chassard C. Lacroix C. (2012). Gut microbial adaptation to dietary consumption of fructose, artificial sweeteners and sugar alcohols: implications for host-microbe interactions contributing to obesity. Obes. Rev. 13, 799–809. 10.1111/j.1467-789X.2012.01009.x, PMID: 22686435

[ref102] Pedersen C. Ijaz U. Z. Gallagher E. Horton F. Ellis R. J. Jaiyeola E.. (2018). Fecal Enterobacteriales enrichment is associated with increased in vivo intestinal permeability in humans. Physiol. Rep. 6:e13649. 10.14814/phy2.13649, PMID: 29611319 PMC5880877

[ref103] Pogorzelski S. Watrobska-Swietlikowska D. Sznitowska M. (2012). Surface tensometry studies on formulations of surfactants with preservatives as a tool for antimicrobial drug protection characterization. J. Biophys. Chem. 3, 324–333. 10.4236/jbpc.2012.34040

[ref104] Props R. Monsieurs P. Mysara M. Clement L. Boon N. (2016). Measuring the biodiversity of microbial communities by flow cytometry. Methods Ecol. Evol. 7, 1376–1385. 10.1111/2041-210X.12607

[ref105] Quast C. Pruesse E. Yilmaz P. Gerken J. Schweer T. Yarza P.. (2013). The SILVA ribosomal RNA gene database project: improved data processing and web-based tools. Nucleic Acids Res. 41, D590–D596. 10.1093/nar/gks1219, PMID: 23193283 PMC3531112

[ref106] Rajagopal S. Eis N. Bhattacharya M. Nickerson K. W. (2003). Membrane-derived oligosaccharides (MDOs) are essential for sodium dodecyl sulfate resistance in *Escherichia coli*. FEMS Microbiol. Lett. 223, 25–31. 10.1016/S0378-1097(03)00323-9, PMID: 12798996

[ref107] Rajagopal S. Sudarsan N. Nickerson K. W. (2002). Sodium dodecyl sulfate hypersensitivity of clpP and clpB mutants of *Escherichia coli*. Appl. Environ. Microbiol. 68, 4117–4121. 10.1128/aem.68.8.4117-4121.2002, PMID: 12147516 PMC124035

[ref108] Ramos H. C. Rumbo M. Sirard J. (2004). Bacterial flagellins: mediators of pathogenicity and host immune responses in mucosa. Trends Microbiol. 12, 510–517. 10.1016/j.tim.2004.09.002, PMID: 15488392

[ref109] Rauber F. da Costa Louzada M. L. Steele E. M. Millett C. Monteiro C. A. Levy R. B. (2018). Ultra-processed food consumption and chronic non-communicable diseases-related dietary nutrient profile in the UK (2008–2014). Nutrients 10:587. 10.3390/nu10050587, PMID: 29747447 PMC5986467

[ref700] R Core Team (2016). R: A Language and Environment for Statistical Computing. R Foundation for Statistical Computing. Available at: https://www.R-project.org

[ref110] Reichardt N. Duncan S. H. Young P. Belenguer A. McWilliam Leitch C. Scott K. P.. (2014). Phylogenetic distribution of three pathways for propionate production within the human gut microbiota. ISME J. 8, 1323–1335. 10.1038/ismej.2014.14, PMID: 24553467 PMC4030238

[ref111] Rodionova I. A. Li X. Thiel V. Stolyar S. Stanton K. Fredrickson J. K.. (2013). Comparative genomics and functional analysis of rhamnose catabolic pathways and regulons in bacteria. Front. Microbiol. 4:407. 10.3389/fmicb.2013.00407, PMID: 24391637 PMC3870299

[ref112] Shin N. R. Whon T. W. Bae J. W. (2015). Proteobacteria: microbial signature of dysbiosis in gut microbiota. Trends Biotechnol. 33, 496–503. 10.1016/j.tibtech.2015.06.011, PMID: 26210164

[ref113] Stremmel W. Robert A. H. Karner E. M. Braun A. (2010). Phosphatidylcholine (lecithin) and the mucus layer: evidence of therapeutic efficacy in ulcerative colitis? Dig. Dis. 28, 490–496. 10.1159/000320407, PMID: 20926877

[ref114] Szymczyk K. Zdziennicka A. Jańczuk B. (2018). Adsorption and aggregation properties of some polysorbates at different temperatures. J. Solut. Chem. 47, 1824–1840. 10.1007/s10953-018-0823-z, PMID: 30524153 PMC6244871

[ref115] Tang W. H. W. Hazen S. L. (2014). The contributory role of gut microbiota in cardiovascular disease find the latest version: the contributory role of gut microbiota in cardiovascular disease. J. Clin. Investig. 124, 4204–4211. 10.1172/JCI72331 25271725 PMC4215189

[ref116] Tito R. Y. Cypers H. Joossens M. Varkas G. Van Praet L. Glorieus E.. (2017). Brief report: dialister as a microbial marker of disease activity in spondyloarthritis. Arthritis Rheum. 69, 114–121. 10.1002/art.39802, PMID: 27390077

[ref117] Tominaga A. Mahmoud M. A. H. Al Mamun A. A. M. Mukaihara T. (2001). Characterization of cryptic flagellin genes in Shigella boydii and Shigella dysenteriae. Genes Genet. Syst. 76, 111–120. 10.1266/ggs.76.111, PMID: 11434456

[ref118] Turnbaugh P. J. Hamady M. Yatsunenko T. Cantarel B. L. Duncan A. Ley R. E.. (2009). A core gut microbiome in obese and lean twins. Nature 457, 480–484. 10.1038/nature07540, PMID: 19043404 PMC2677729

[ref119] Van Bogaert I. (2008). Microbial synthesis of sophorolipids by the yeast candida bombicola. Ghent University.

[ref120] Van Bogaert I. Zhang J. Soetaert W. (2011). Microbial synthesis of sophorolipids. Process Biochem. 46, 821–833. 10.1016/j.procbio.2011.01.010

[ref123] Vandeputte D. Kathagen G. D’Hoe K. Vieira-Silva S. Valles-Colomer M. Sabino J.. (2017). Quantitative microbiome profiling links gut community variation to microbial load. Nature 551, 507–511. 10.1038/nature24460, PMID: 29143816

[ref121] Van Haesendonck I. Vanzeveren E. C. A. (2006). (12) Patent Application Publication (10) Pub. No.: US 2006/0233935 A1.1(19).

[ref122] Van Nevel S. Koetzsch S. Weilenmann H. U. Boon N. Hammes F. (2013). Routine bacterial analysis with automated flow cytometry. J. Microbiol. Methods 94, 73–76. 10.1016/j.mimet.2013.05.007, PMID: 23684992

[ref124] Verhoog S. Taneri P. E. Díaz Z. M. R. Marques-Vidal P. Troup J. P. Bally L.. (2019). Dietary factors and modulation of bacteria strains of *Akkermansia muciniphila* and faecalibacterium prausnitzii: a systematic review. Nutrients 11:1565. 10.3390/nu11071565, PMID: 31336737 PMC6683038

[ref125] Vilchez-Vargas R. Geffers R. Suárez-Diez M. Conte I. Waliczek A. Kaser V. S.. (2013). Analysis of the microbial gene landscape and transcriptome for aromatic pollutants and alkane degradation using a novel internally calibrated microarray system. Environ. Microbiol. 15, 1016–1039. 10.1111/j.1462-2920.2012.02752.x, PMID: 22515215

[ref126] Vu K. A. Tawfiq K. Chen G. (2015). Rhamnolipid transport in biochar-amended agricultural soil. Water Air Soil Pollut. 226, 1–8. 10.1007/s11270-015-2497-0

[ref127] Wang L. Christophersen C. T. Sorich M. J. Gerber J. P. Angley M. T. Conlon M. A. (2013). Increased abundance of Sutterella spp. and Ruminococcus torques in feces of children with autism spectrum disorder. Mol. Autism 4:42. 10.1186/2040-2392-4-42, PMID: 24188502 PMC3828002

[ref128] Wang Z. Klipfell E. Bennett B. J. Koeth R. Levison B. S. Dugar B.. (2011). Gut flora metabolism of phosphatidylcholine promotes cardiovascular disease. Nature 472, 57–65. 10.1038/nature09922, PMID: 21475195 PMC3086762

[ref129] Ward T. Larson J. Meulemans J. Hillman B. Lynch J. Sidiripoulos D. (2017). BugBase predicts organism-level microbiome phenotypes. bioRxiv [preprint]. 10.1101/133462

[ref130] Waterlander W. E. Ni Mhurchu C. Eyles H. Vandevijvere S. Cleghorn C. Scarborough P. (2018). Food futures: developing effective food systems interventions to improve public health nutrition. Agric. Syst. 160, 124–131. 10.1016/j.agsy.2017.01.006

[ref131] Weitkunat K. Schumann S. Nickel D. Kappo K. A. Petzke K. J. Kipp A. P.. (2016). Importance of propionate for the repression of hepatic lipogenesis and improvement of insulin sensitivity in high-fat diet-induced obesity. Mol. Nutr. Food Res. 60, 2611–2621. 10.1002/mnfr.201600305, PMID: 27467905 PMC5215627

[ref132] Wlodkovic D. Skommer J. Darzynkiewicz Z. (2009). Flow cytometry-based apoptosis detection Donald. Mol. Biol. 559, 313–332. 10.1007/978-1-60327-017-5 PMC3863590 19609746

[ref133] Wu Y. Wang T. (2003). Soybean lecithin fractionation and functionality. J. Am. Oil Chem. Soc. 80, 319–326. 10.1007/s11746-003-0697-x

[ref134] Yao C. K. Muir J. G. Gibson P. R. (2016). Review article: insights into colonic protein fermentation, its modulation and potential health implications. Aliment. Pharmacol. Ther. 43, 181–196. 10.1111/apt.13456, PMID: 26527169

[ref800] Zhang H. Dudley E. G. Harte F. (2017). Critical synergistic concentration of lecithin phospholipids improves the antimicrobial activity of eugenol against *Escherichia coli*. Appl. Environ. Microbiol. 83, 1–9. 10.1128/AEM.01583-17, PMID: 28842540 PMC5648912

