# Supplementary material for: Dietary Emulsifiers Alter Composition and Activity of the Human Gut Microbiota in vitro, Irrespective of Chemical or Natural Emulsifier Origin
Source: Front Microbiol. 2020 Nov 5;11:577474. doi: 10.3389/fmicb.2020.577474 (PMC7676226; doi:10.3389/fmicb.2020.577474)
Supplement: Supplementary file 1 [file Data_Sheet_1.pdf]

Supplementary Material

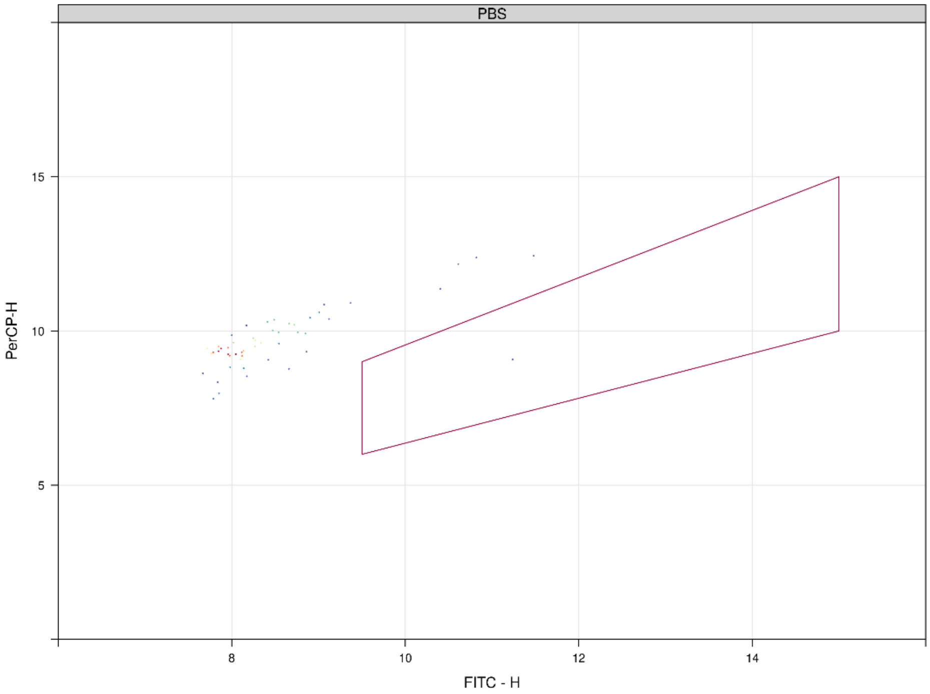

Figure S1: Density plot of cell counts for the PBS control sample measured in conjuction with samples from for *in vitro* batch incubations of fecal material from 10 donors with sugar depleted medium supplemented with 5 emulsifiers at 4 concentrations. The gate represents intact cell counts. The lack of counts in this gate indicates limited background from the PBS-matrix.

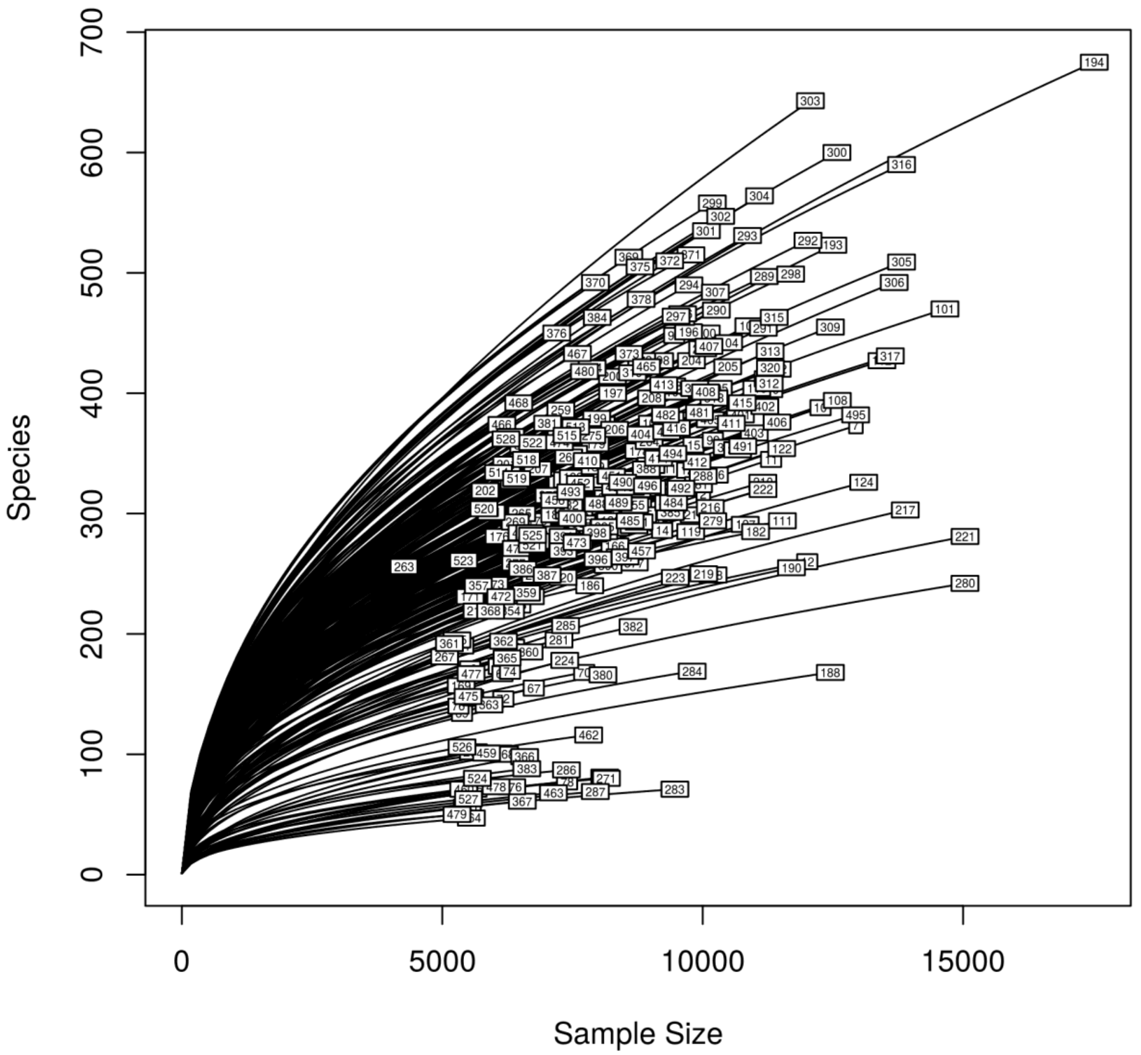

Figure S2: Rarefaction curve of 16S rRNA gene amplicon sequencing data after copy number correction on the data from *in vitro* batch incubations of fecal material from 10 donors with sugar depleted medium supplemented with 5 emulsifiers at 4 concentrations. The numbers on the curves represent sample names which can be looked up in the NCBI submission with the accession code PRJNA630547.

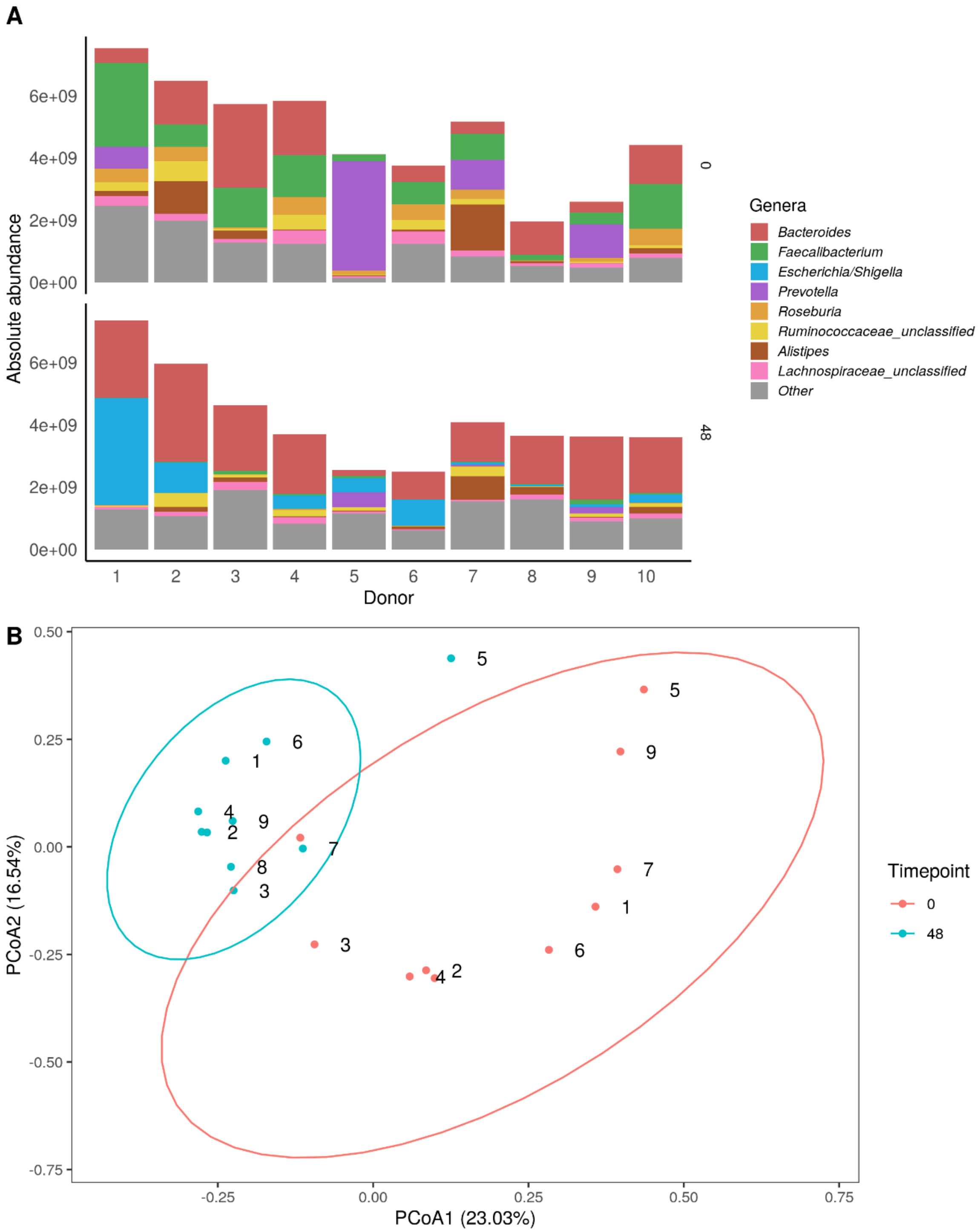

Figure S3: A: Quantitative microbial profiling (QMP) renerated from both 16S rRNA sequencing data and flow cytometric data for control samples from 10 donors at 0h (upper barplot) and after 48h (lower barplot) of the *in vitro* batch incubations of fecal material with sugar depleted medium supplemented with 5 emulsifiers at 4 concentrations. Samples for 16S sequencing were taken upon incubation (0h) as well as after 48h of incubation. B: Principle coordinate analysis of genus based QMP-data of control samples. Labels indicate donors.

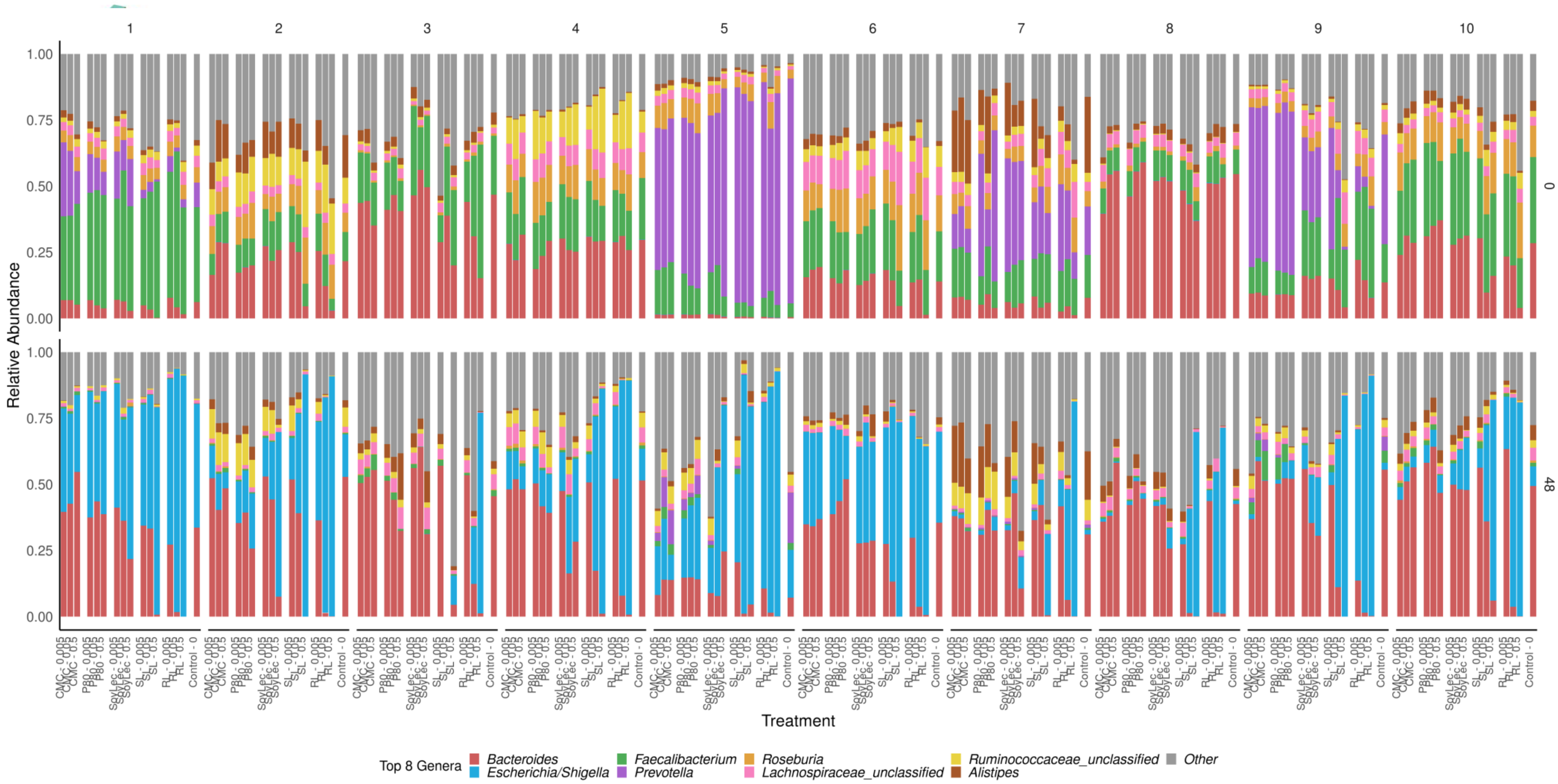

Figuur S4: Relative abundances of top 8 genera derived from 16S rRNA gene amplicon sequengcing measured at the start (0h) and at the end (48h) of the *in vitro* batch incubations of fecal material from 10 donors with sugar depleted medium supplemented with 5 emulsifiers at 4 concentrations. Samples for 16S sequencing were taken upon incubation (0h) as well as after 48h of incubation. CMC = sodium carboxymethylcellulose, P80 = polysorbate80, SoyLec = soy lecithin, SL = sophorolipids, RL = rhamnolipids.

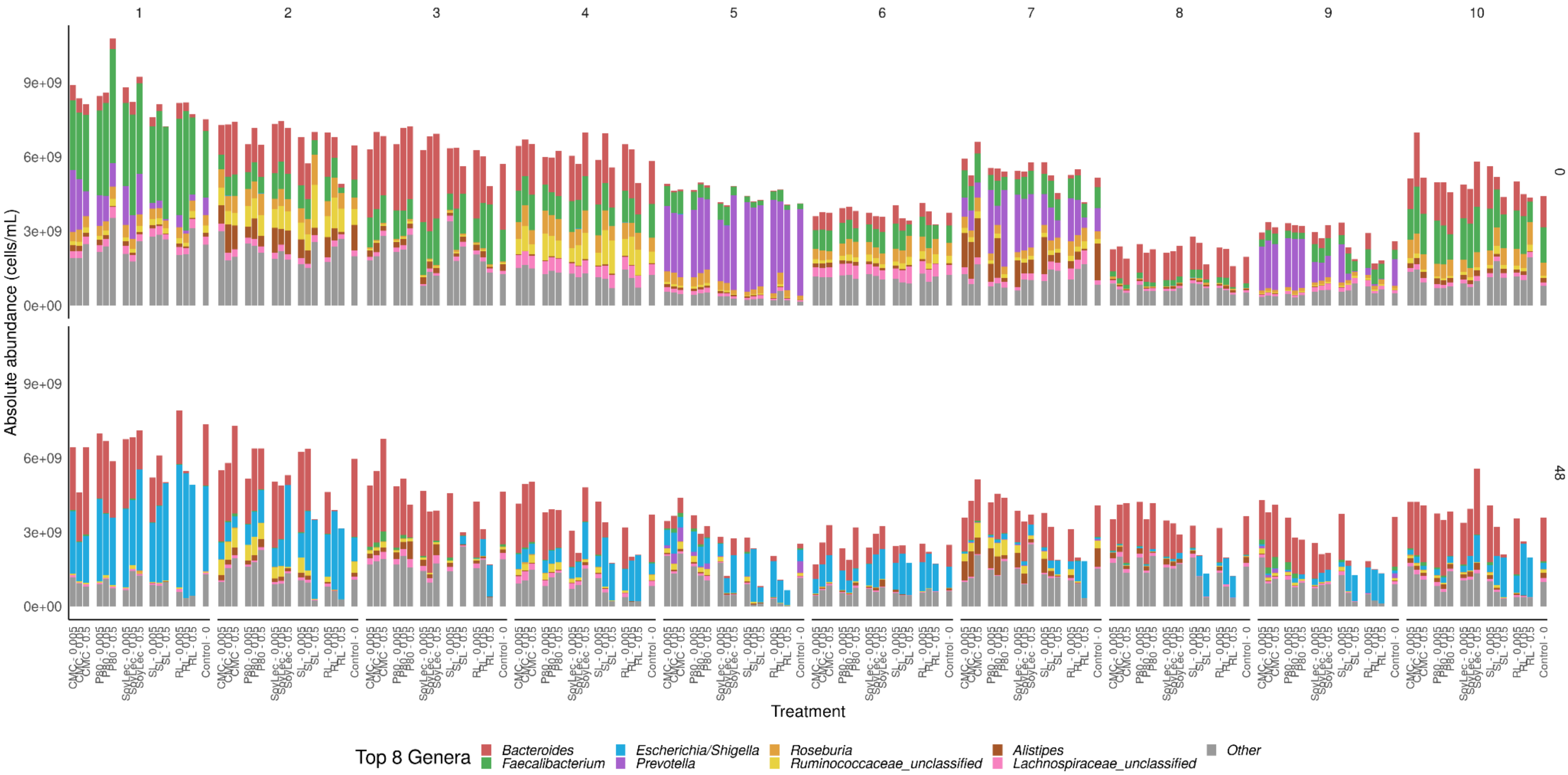

Figuur S5: Absolute abundances of top 8 genera derived from 16S rRNA gene amplicon sequengcing measured at the start (0h) and at the end (48h) of the *in vitro* batch incubations of fecal material from 10 donors with sugar depleted medium supplemented with 5 emulsifiers at 4 concentrations. Samples for 16S sequencing were taken upon incubation (0h) as well as after 48h of incubation. CMC = sodium carboxymethylcellulose, P80 = polysorbate80, SoyLec = soy lecithin, SL = sophorolipids, RL = rhamnolipids.

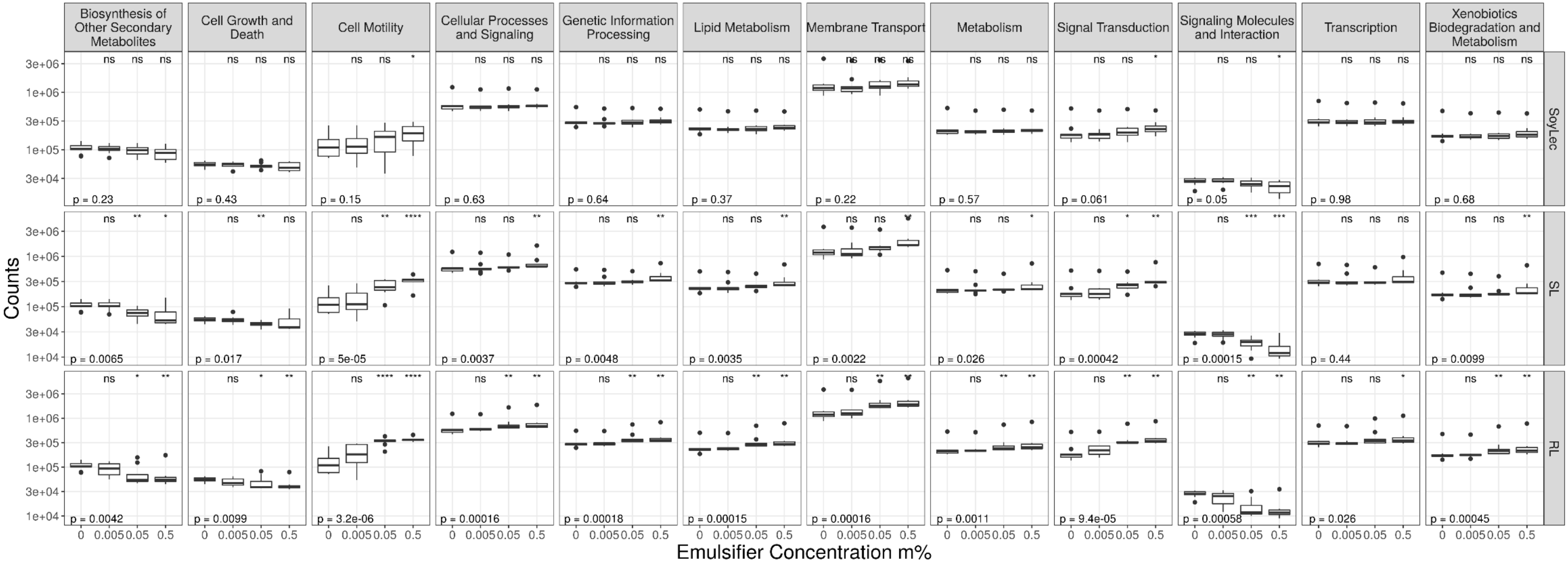

Figuur S6:Phenotypic prediction of gut microbial communities after 48h of *in vitro* batch incubations of fecal material from 10 donors with sugar depleted medium supplemented with 5 emulsifiers at 4 concentrations. Prediction of microbial functionalities was made using PICRUSt, based on the Kyoto Encyclopedia of Genes and Genomes (KEGG) database. Significantly different metagenomic pathways at KEGG level 2 are given for soy lecithin, sophorolipids (SL) and rhamnolipids (RL) (no significant differences were observed for carboxymehtyl cellulose of polysorbate 80). P-values represent results of Kruskal-Wallis test, asterisks represent results from Wilcoxon Rank Sum test of comparisons with control (α = 0.05).

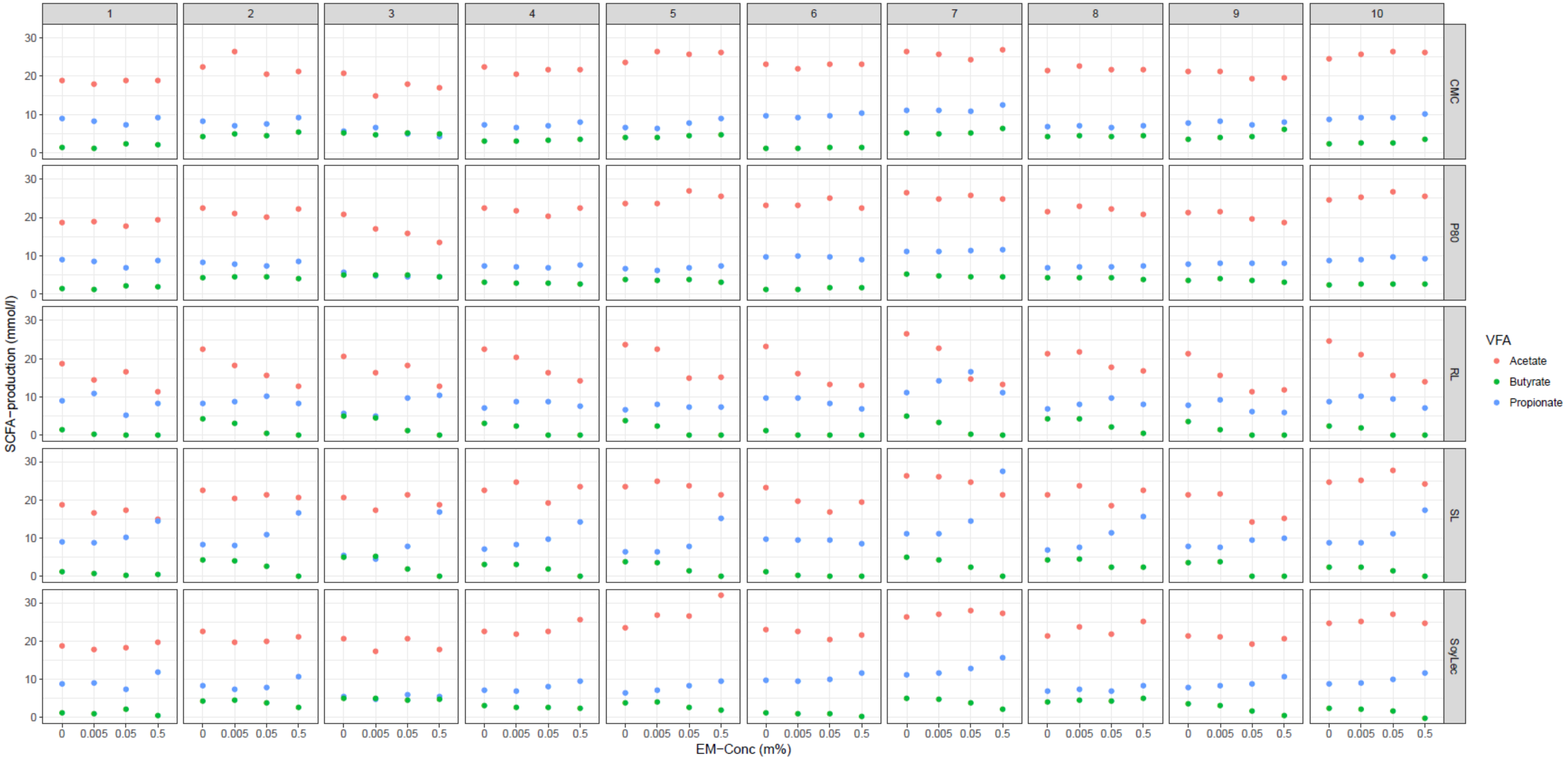

Figuur S7: Short chain fatty acid production over 48h *in vitro* batch incubations of fecal material from 10 donors with sugar depleted medium supplemented with 5 emulsifiers at 4 concentrations. CMC = sodium carboxymethylcellulose, P80 = polysorbate80, SoyLec = soy lecithin, SL = sophorolipids, RL = rhamnolipids.

Table S1: Intact cell counts (logarithmic) and percentage of surviving cells on the three measured timepoints for *in vitro* batch incubations of fecal material from 10 donors with sugar depleted medium supplemented with 5 emulsifiers at 4 concentrations. Samples were taken upon incubation (T0; 2-3h after inoculation) as well as after 24h (T1) and 48h (T2) of incubation. Concentrations of emulsifiers are given in % (m/v). CMC = sodium carboxymethylcellulose, P80 = polysorbate80, SoyLec = soy lecithin, SL = sophorolipids, RL = rhamnolipids.

	0h		24h		48h			
	Mean of Intact cells	Cell count / Cell count control-T0	Mean of Intact cells	Cell count T1/ Cell count control-T0	Mean of Intact cells	Cell count T2/ Cell count T0	Cell count T2 / Cell count control-T0	Cell count T2 / Cell count control-T2
**CMC**								
0	9.48 ± 0.23	100% ± 3%	9.52 ± 0.23	108% ± 4%	9.38 ± 0.20	79% ± 3%	79% ± 3%	100% ± 3%
0.005	9.56 ± 0.23	119% ± 4%	9.55 ± 0.21	98% ± 3%	9.40 ± 0.26	69% ± 3%	82% ± 3%	104% ± 4%
0.05	9.59 ± 0.23	128% ± 4%	9.59 ± 0.22	99% ± 3%	9.44 ± 0.23	71% ± 2%	91% ± 3%	115% ± 4%
0.5	9.56 ± 0.25	119% ± 4%	9.60 ± 0.21	111% ± 4%	9.49 ± 0.22	85% ± 3%	101% ± 3%	128% ± 4%
**P80**								
0	9.48 ± 0.23	100% ± 3%	9.52 ± 0.23	108% ± 4%	9.38 ± 0.20	79% ± 3%	79% ± 3%	100% ± 3%
0.005	9.54 ± 0.21	114% ± 4%	9.51 ± 0.19	93% ± 3%	9.39 ± 0.22	71% ± 2%	81% ± 3%	102% ± 3%
0.05	9.55 ± 0.20	117% ± 4%	9.50 ± 0.24	89% ± 3%	9.36 ± 0.21	64% ± 2%	75% ± 2%	95% ± 3%
0.5	9.57 ± 0.19	121% ± 4%	9.51 ± 0.14	88% ± 2%	9.42 ± 0.15	71% ± 2%	86% ± 2%	109% ± 3%
**SoyLec**								
0	9.48 ± 0.23	100% ± 3%	9.52 ± 0.23	108% ± 4%	9.38 ± 0.20	79% ± 3%	79% ± 3%	100% ± 3%
0.005	9.47 ± 0.27	96% ± 4%	9.41 ± 0.21	88% ± 3%	9.27 ± 0.19	64% ± 2%	61% ± 2%	78% ± 2%
0.05	9.40 ± 0.34	82% ± 4%	9.21 ± 0.21	65% ± 3%	9.04 ± 0.25	44% ± 2%	36% ± 1%	46% ± 2%
0.5	9.44 ± 0.29	92% ± 4%	9.08 ± 0.11	43% ± 1%	8.85 ± 0.13	25% ± 1%	23% ± 1%	30% ± 1%
**SL**								
0	9.48 ± 0.23	100% ± 3%	9.52 ± 0.23	108% ± 4%	9.38 ± 0.20	79% ± 3%	79% ± 3%	100% ± 3%
0.005	9.57 ± 0.18	122% ± 4%	9.52 ± 0.21	89% ± 3%	9.37 ± 0.23	64% ± 2%	78% ± 3%	98% ± 3%
0.05	9.19 ± 0.37	51% ± 2%	9.23 ± 0.15	110% ± 5%	9.07 ± 0.16	76% ± 3%	39% ± 1%	49% ± 1%
0.5	8.80 ± 0.46	21% ± 1%	8.88 ± 0.17	120% ± 7%	8.79 ± 0.19	99% ± 6%	21% ± 1%	26% ± 1%
**RL**								
0	9.48 ± 0.23	100% ± 3%	9.52 ± 0.23	108% ± 4%	9.38 ± 0.20	79% ± 3%	79% ± 3%	100% ± 3%
0.005	9.42 ± 0.33	86% ± 4%	9.32 ± 0.21	79% ± 3%	9.12 ± 0.20	50% ± 2%	43% ± 1%	55% ± 2%
0.05	9.00 ± 0.37	33% ± 2%	9.00 ± 0.12	100% ± 5%	8.73 ± 0.13	53% ± 2%	18% ± 0%	22% ± 1%
0.5	8.59 ± 0.43	13% ± 1%	8.80 ± 0.22	162% ± 9%	8.45 ± 0.20	72% ± 4%	9% ± 0%	12% ± 0%

Table S2: Total cell counts (logarithmic) and percentage of remaining cells on the three measured timepoints for *in vitro* batch incubations of fecal material from 10 donors with sugar depleted medium supplemented with 5 emulsifiers at 4 different concentrations. Samples were taken upon incubation (T0; 2-3h after inoculation) as well as after 24h (T1) and 48h (T2) of incubation. Concentrations of emulsifiers are given in % (m/v). CMC = sodium carboxymethylcellulose, P80 = polysorbate80, SoyLec = soy lecithin, SL = sophorolipids, RL = rhamnolipids.

	0h		24h		48h			
	Mean of Total Cells	Cell count / Cell count control-T0	Mean of Intact cells	Cell count T1 / Cell count control-T0	Mean of Intact cells	Cell count T2 / Cell count T0	Cell count T2 / Cell count control-T0	Cell count T2 / Cell count control-T2
**CMC**								
0	9.66 ± 0.16	100% + 2%	9.70 ± 0.10	110% + 2%	9.59 ± 0.10	86% + 2%	86% + 2%	100% + 1%
0.005	9.71 ± 0.18	112% + 3%	9.71 ± 0.12	101% + 2%	9.62 ± 0.15	81% + 2%	91% + 2%	105% + 2%
0.05	9.73 ± 0.16	118% + 3%	9.75 ± 0.11	103% + 2%	9.65 ± 0.12	83% + 2%	98% + 2%	114% + 2%
0.5	9.71 ± 0.19	113% + 3%	9.77 ± 0.10	115% + 3%	9.71 ± 0.11	99% + 2%	112% + 2%	130% + 2%
**P80**								
0	9.66 ± 0.16	100% + 2%	9.70 ± 0.10	110% + 2%	9.59 ± 0.10	86% + 2%	86% + 2%	100% + 1%
0.005	9.71 ± 0.14	112% + 3%	9.69 ± 0.09	96% + 2%	9.62 ± 0.10	82% + 1%	92% + 2%	107% + 2%
0.05	9.71 ± 0.16	112% + 3%	9.70 ± 0.15	98% + 2%	9.58 ± 0.14	74% + 2%	83% + 2%	96% + 2%
0,5	9.71 ± 0.17	113% + 3%	9.70 ± 0.09	96% + 3%	9.62 ± 0.09	80% + 2%	90% + 2%	105% + 1%
**SoyLec**								
0	9.66 ± 0.16	100% + 2%	9.70 ± 0.10	110% + 2%	9.59 ± 0.10	86% + 2%	86% + 2%	100% + 1%
0.005	9.69 ± 0.17	106% + 3%	9.67 ± 0.12	96% + 2%	9.57 ± 0.11	76% + 2%	81% + 2%	94% + 1%
0.05	9.68 ± 0.17	106% + 3%	9.60 ± 0.12	82% + 2%	9.48 ± 0.18	62% + 2%	66% + 2%	76% + 2%
0.5	9.73 ± 0.17	118% + 3%	9.63 ± 0.08	78% + 2%	9.55 ± 0.12	65% + 1%	77% + 2%	89% + 1%
**SL**								
0	9.66 ± 0.16	100% + 2%	9.70 ± 0.10	110% + 2%	9.59 ± 0.10	86% + 2%	86% + 2%	100% + 1%
0.005	9.72 ± 0.14	115% + 3%	9.71 ± 0.12	97% + 2%	9.60 ± 0.13	76% + 1%	87% + 2%	101% + 2%
0.05	9.68 ± 0.16	105% + 2%	9.59 ± 0.13	82% + 2%	9.49 ± 0.15	64% + 1%	67% + 2%	78% + 1%
0.5	9.60 ± 0.19	87% + 2%	9.37 ± 0.16	59% + 2%	9.26 ± 0.20	45% + 1%	40% + 1%	46% + 1%
**RL**								
0	9.66 ± 0.16	100% + 2%	9.70 ± 0.10	110% + 2%	9.59 ± 0.10	86% + 2%	86% + 2%	100% + 1%
0.005	9.70 ± 0.16	110% + 2%	9.64 ± 0.13	87% + 2%	9.51 ± 0.13	64% + 1%	71% + 2%	82% + 1%
0.05	9.65 ± 0.19	98% + 3%	9.47 ± 0.14	67% + 2%	9.32 ± 0.15	47% + 1%	46% + 1%	54% + 1%
0.5	9.56 ± 0.17	80% + 2%	9.33 ± 0.13	58% + 1%	9.22 ± 0.20	45% + 1%	36% + 1%	42% + 1%

Table S3: P-values resulting from Wilcoxon Rank Sum tests (α = 0.05) comparing the effect of dietary emulsifiers on total and intac cell counts for *in vitro* batch incubations of fecal material from 10 donors with sugar depleted medium supplemented with 5 emulsifiers at 4 concentrations. Samples were taken upon incubation (T0; 2-3h after inoculation) as well as after 24h (T1) and 48h (T2) of incubation. P-values refer to comparisons of the treatments with the control (0 % (m/v) emulsifier). Significant p-values are indicated in bold.

**Total cell concentration**				**Intact cell concentration**			
	**T0**	**T1**	**T2**		**T0**	**T1**	**T2**
**CMC - 0.005**	0.290	0.680	0.410	**CMC - 0.005**	0.200	0.400	0.300
**CMC - 0.05**	0.130	0.091	0.056	**CMC - 0.05**	0.110	0.081	0.030
**CMC - 0.5**	0.140	**0.020**	**0.004**	**CMC - 0.5**	0.150	0.056	**0.018**
**P80 - 0.005**	0.290	0.970	0.290	**P80 - 0.005**	0.340	0.600	0.860
**P80 - 0.05**	0.300	0.860	0.780	**P80 - 0.05**	0.300	0.530	0.530
**P80 - 0.5**	0.350	0.620	0.560	**P80 - 0.5**	0.250	0.350	0.700
**SoyLec - 0.005**	0.490	0.150	0.370	**SoyLec - 0.005**	0.900	**0.020**	**0.028**
**SoyLec - 0.05**	0.530	**0.002**	**0.020**	**SoyLec - 0.05**	0.440	**<0.001**	**<0.001**
**SoyLec - 0.5**	0.096	**0.010**	0.260	**SoyLec - 0.5**	0.600	**<0.001**	**<0.001**
**SL - 0.005**	0.200	0.860	0.880	**SL - 0.005**	0.170	0.840	0.640
**SL - 0.05**	0.700	**0.003**	**0.006**	**SL - 0.05**	**0.004**	**<0.001**	**<0.001**
**SL - 0.5**	0.260	**<0.001**	**<0.001**	**SL - 0.5**	**<0.001**	**<0.001**	**<0.001**
**RL - 0.005**	0.380	0.150	**0.024**	**RL - 0.005**	0.760	**<0.001**	**<0.001**
**RL - 0.05**	0.930	**<0.001**	**<0.001**	**RL - 0.05**	**<0.001**	**<0.001**	**<0.001**
**RL - 0.5**	**0.040**	**<0.001**	**<0.001**	**RL - 0.5**	**<0.001**	**<0.001**	**<0.001**

Table S4: P-values resulting from Wilcoxon Rank Sum tests (α = 0.05) comparing the effect of equivalent emulsifier concentrations on short chain fatty acid concentrations, cell counts and the principal OTU detected using amplicon sequencing detected for *in vitro* batch incubations of fecal material from 10 donors with sugar depleted medium supplemented with 5 emulsifiers at 4 concentrations. Significant p-values are indicated in bold.

**Acetate**				
Vs.	**RL - 0.005**	**SL - 0.005**	**RL - 0.05**	**SL - 0.05**
**CMC - 0.05**	0,079	0,720		
**CMC - 0.5**			**0,001**	0,393
**P80 - 0.05**	0,105	0,971		
**P80 - 0.5**			**0,002**	0,481

**Propionate**				
Vs.	**RL - 0.005**	**SL - 0.005**	**RL - 0.05**	**SL - 0.05**
**CMC - 0.05**	0,211	0,968		
**CMC - 0.5**			0,912	0,218
**P80 - 0.05**	0,190	0,853		
**P80 - 0.5**			0,436	0,089

**Butyrate**				
Vs.	**RL - 0.005**	**SL - 0.005**	**RL - 0.05**	**SL - 0.05**
**CMC - 0.05**	**0,013**	0,720		
**CMC - 0.5**			**0,000**	**0,000**
**P80 - 0.05**	**0,011**	0,853		
**P80 - 0.5**			**0,000**	**0,000**

**Intact cell concentration**				
Vs.	**RL - 0.005**	**SL - 0.005**	**RL - 0.05**	**SL - 0.05**
**CMC - 0.05**	**0,000**	0,102		
**CMC - 0.5**			**0,000**	**0,000**
**P80 - 0.05**	**0,000**	0,620		
**P80 - 0.5**			**0,000**	**0,000**

**Total cell concentration**				
Vs.	**RL - 0.005**	**SL - 0.005**	**RL - 0.05**	**SL - 0.05**
**CMC - 0.05**	**0,000**	0,201		
**CMC - 0.5**			**0,000**	**0,000**
**P80 - 0.05**	0,221	0,718		
**P80 - 0.5**			**0,005**	0,678

**Absolute abundance *Escherichia/Shigella***				
Vs.	**RL - 0.005**	**SL - 0.005**	**RL - 0.05**	**SL - 0.05**
**CMC - 0.05**	0,123	0,481		
**CMC - 0.5**			**0,002**	**0,004**
**P80 - 0.05**	0,143	0,912		
**P80 - 0.5**			**0,009**	**0,017**

**Relative abundance *Escherichia/Shigella***				
Vs.	**RL - 0.005**	**SL - 0.005**	**RL - 0.05**	**SL - 0.05**
**CMC - 0.05**	0,063	0,579		
**CMC - 0.5**			**0,000**	**0,000**
**P80 - 0.05**	0,063	0,853		
**P80 - 0.5**			**0,000**	**0,001**
