# Supplementary figures and images for: Dietary Emulsifiers Alter Composition and Activity of the Human Gut Microbiota in vitro, Irrespective of Chemical or Natural Emulsifier Origin
Source: Front Microbiol. 2020 Nov 5;11:577474. doi: 10.3389/fmicb.2020.577474 (PMC7676226; doi:10.3389/fmicb.2020.577474)

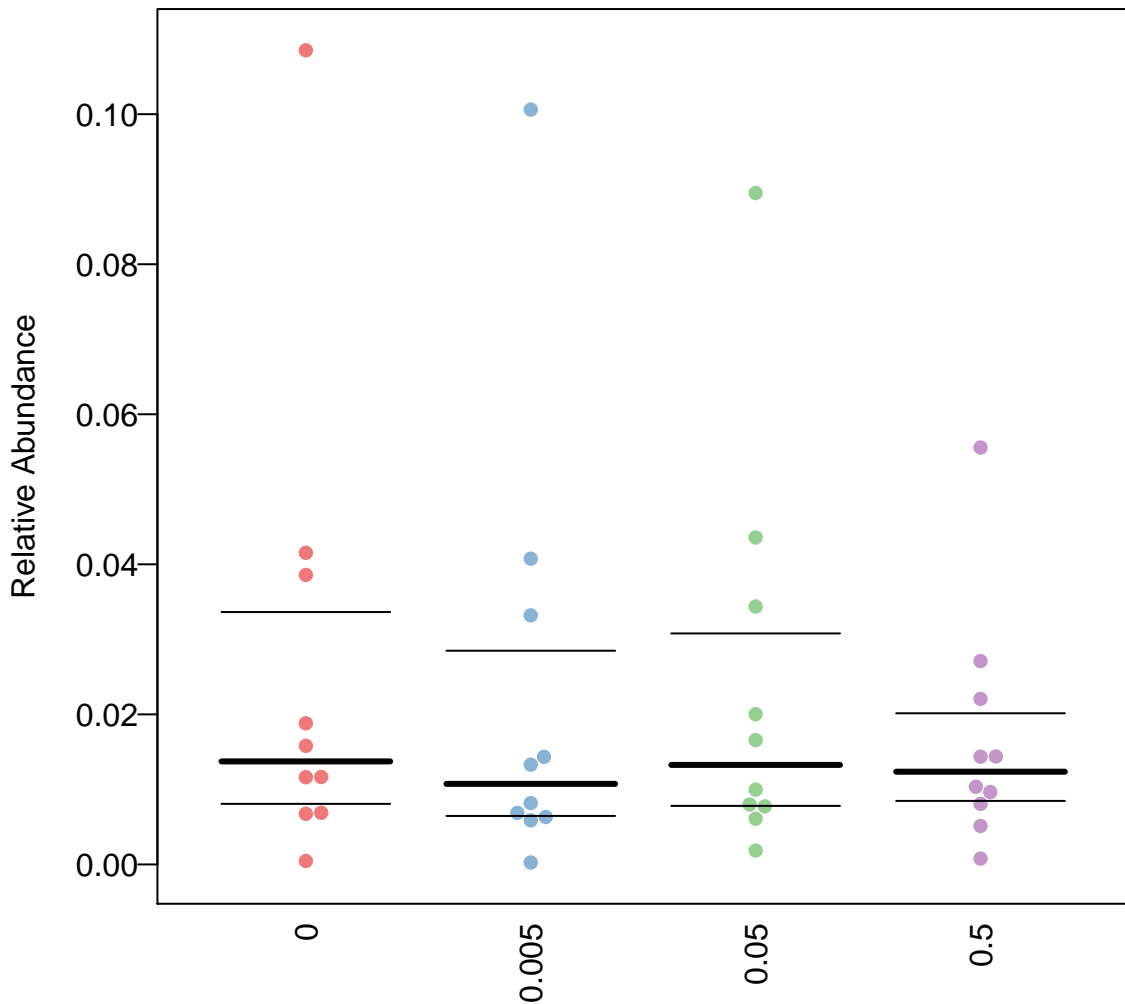

Supplement: Supplementary file 2 [file Data_Sheet_2.zip › predicted_phenotypes_CMC/Aerobic.pdf]

Relative Abundance

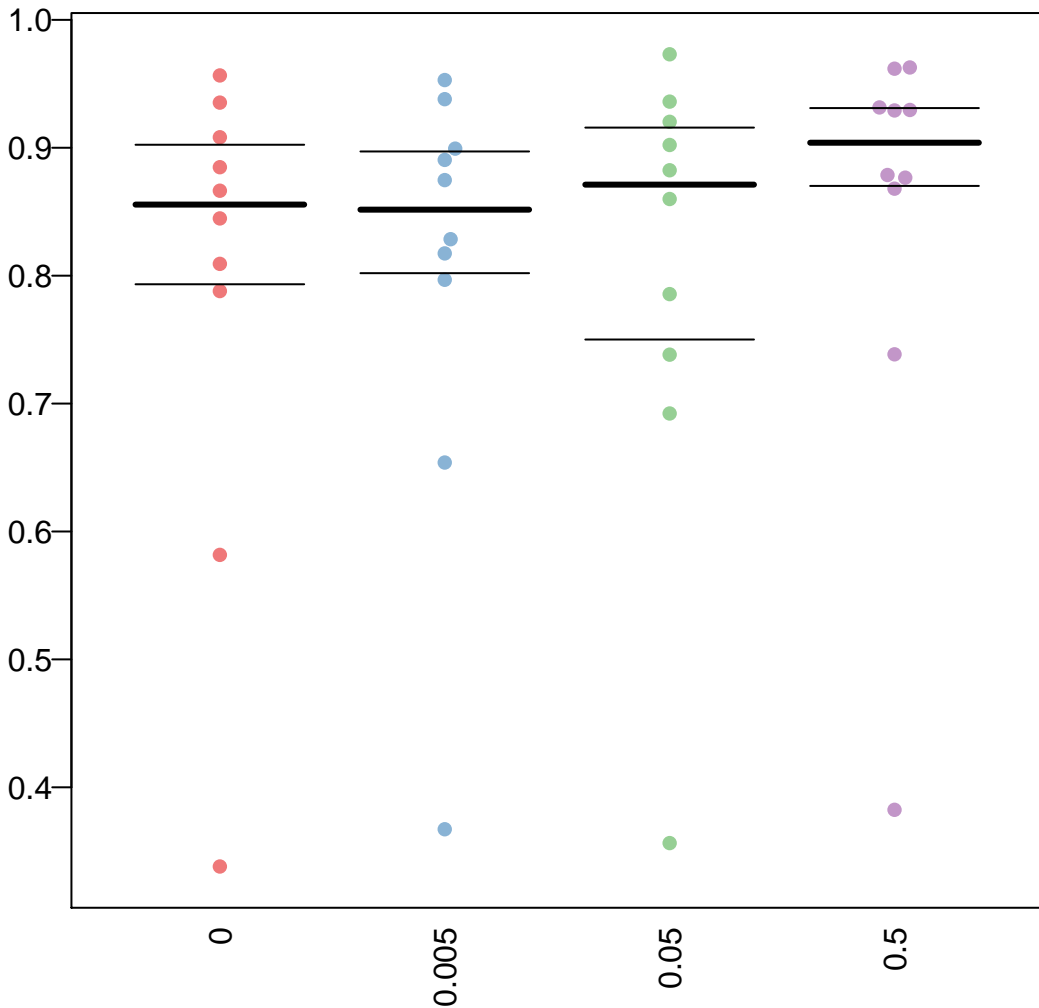

Supplement: Supplementary file 2 [file Data_Sheet_2.zip › predicted_phenotypes_CMC/Anaerobic.pdf]

Relative Abundance

0.6  
0.5  
0.4  
0.3  
0.2  
0.1  
0.0

0

0.005

0.05

0.5

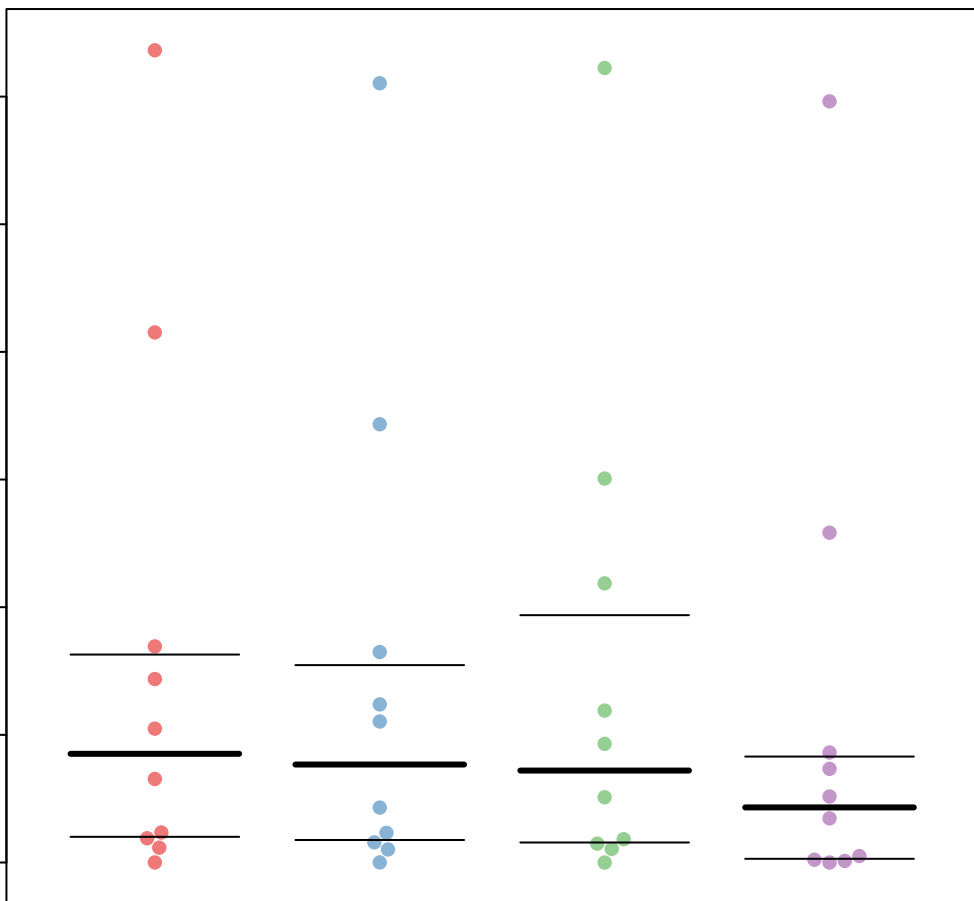

Supplement: Supplementary file 2 [file Data_Sheet_2.zip › predicted_phenotypes_CMC/Contains_Mobile_Elements.pdf]

Relative Abundance

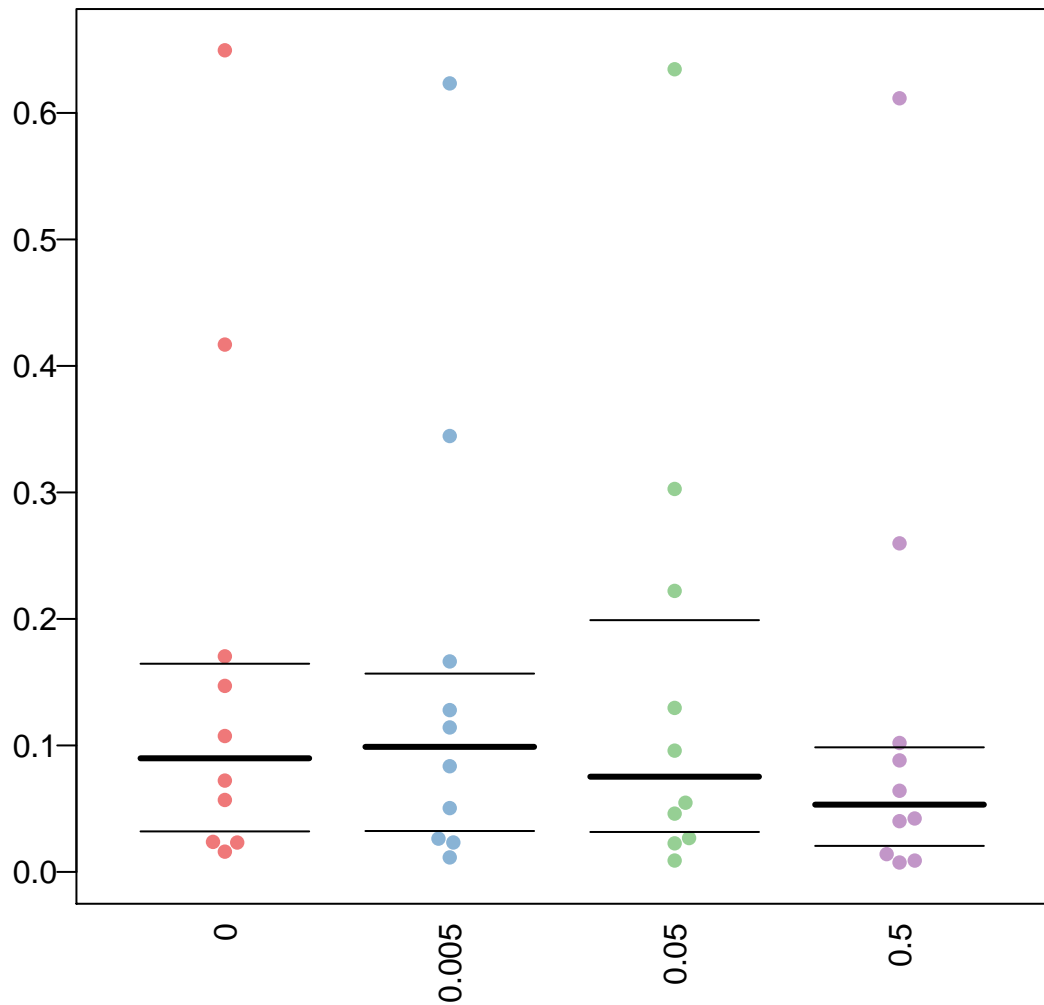

Supplement: Supplementary file 2 [file Data_Sheet_2.zip › predicted_phenotypes_CMC/Facultatively_Anaerobic.pdf]

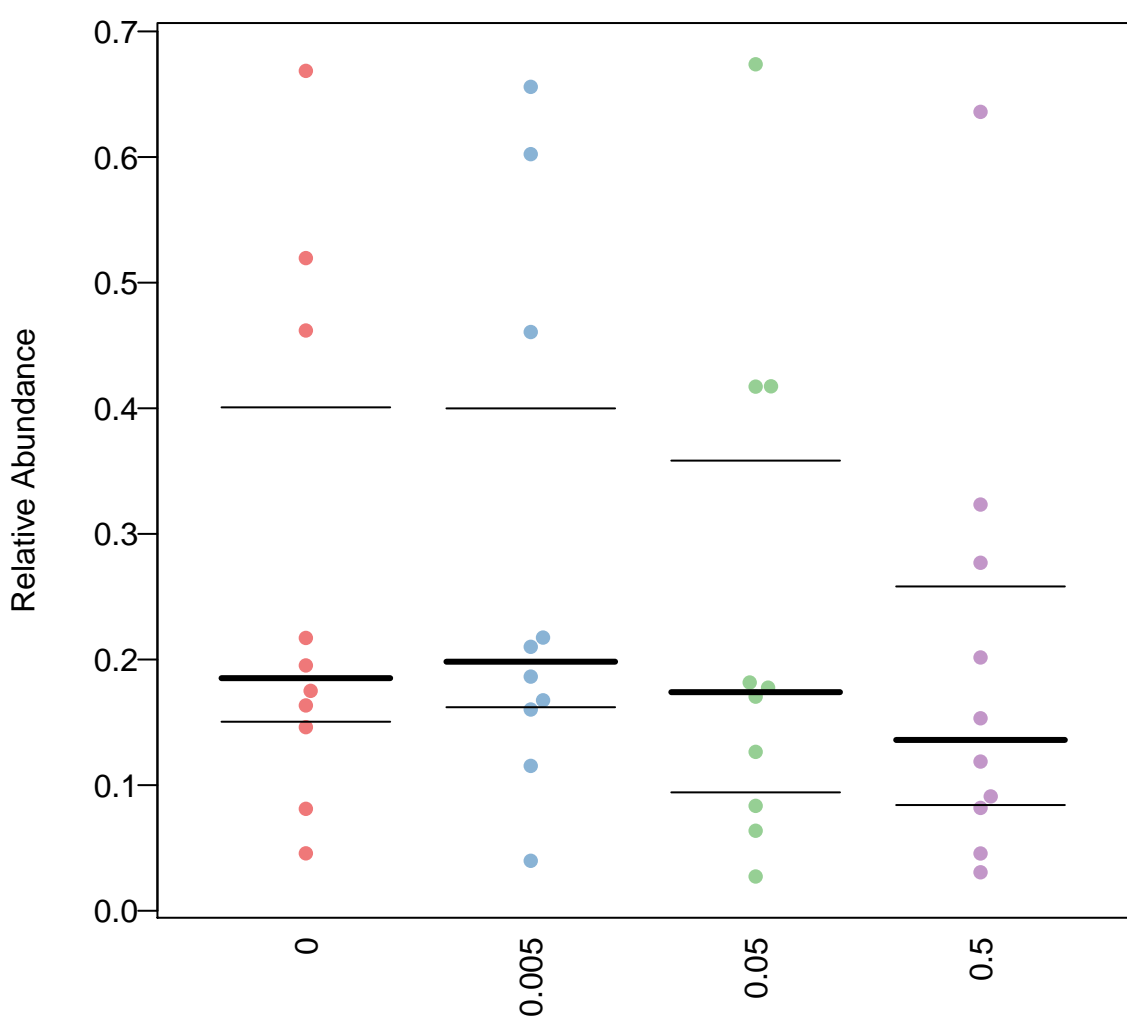

Supplement: Supplementary file 2 [file Data_Sheet_2.zip › predicted_phenotypes_CMC/Forms_Biofilms.pdf]

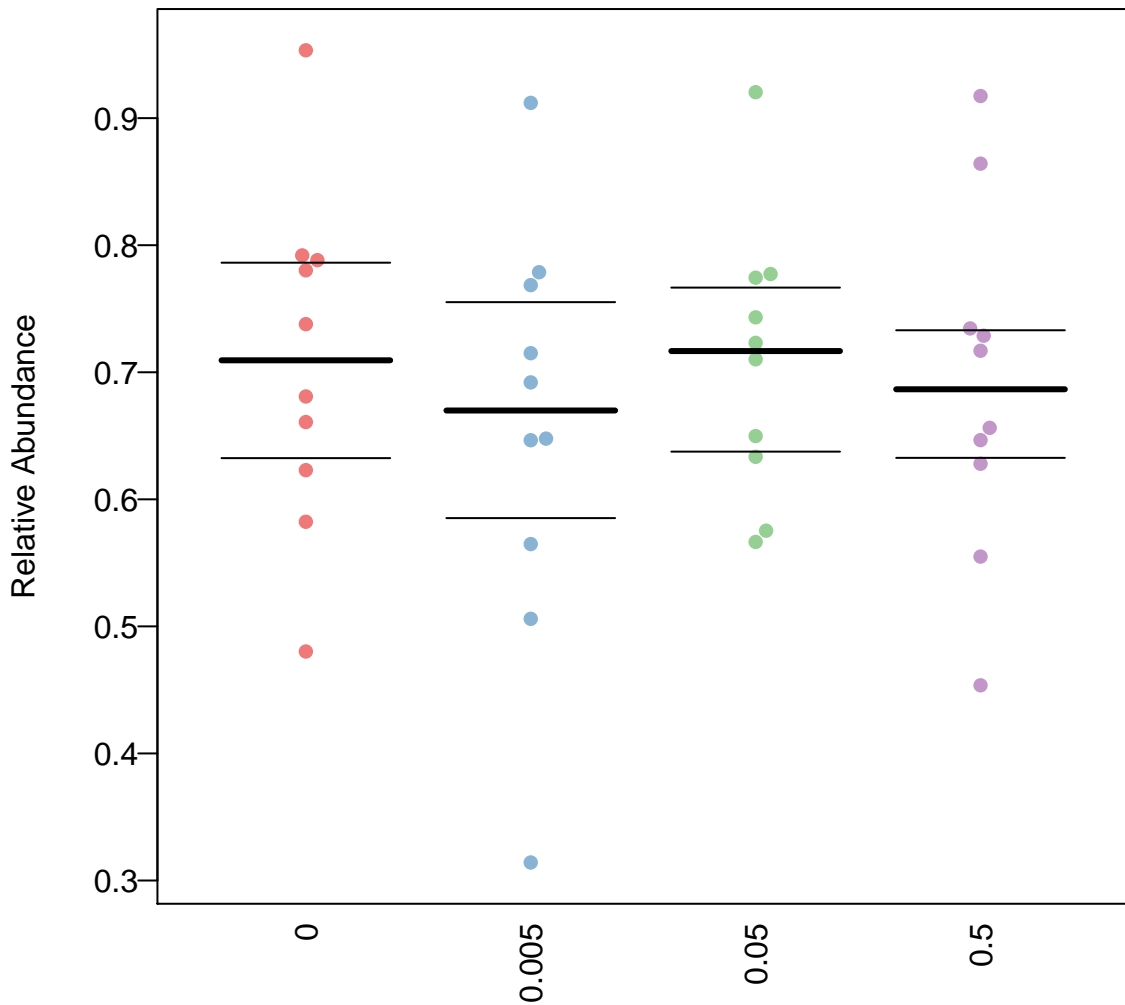

Supplement: Supplementary file 2 [file Data_Sheet_2.zip › predicted_phenotypes_CMC/Gram_Negative.pdf]

Relative Abundance

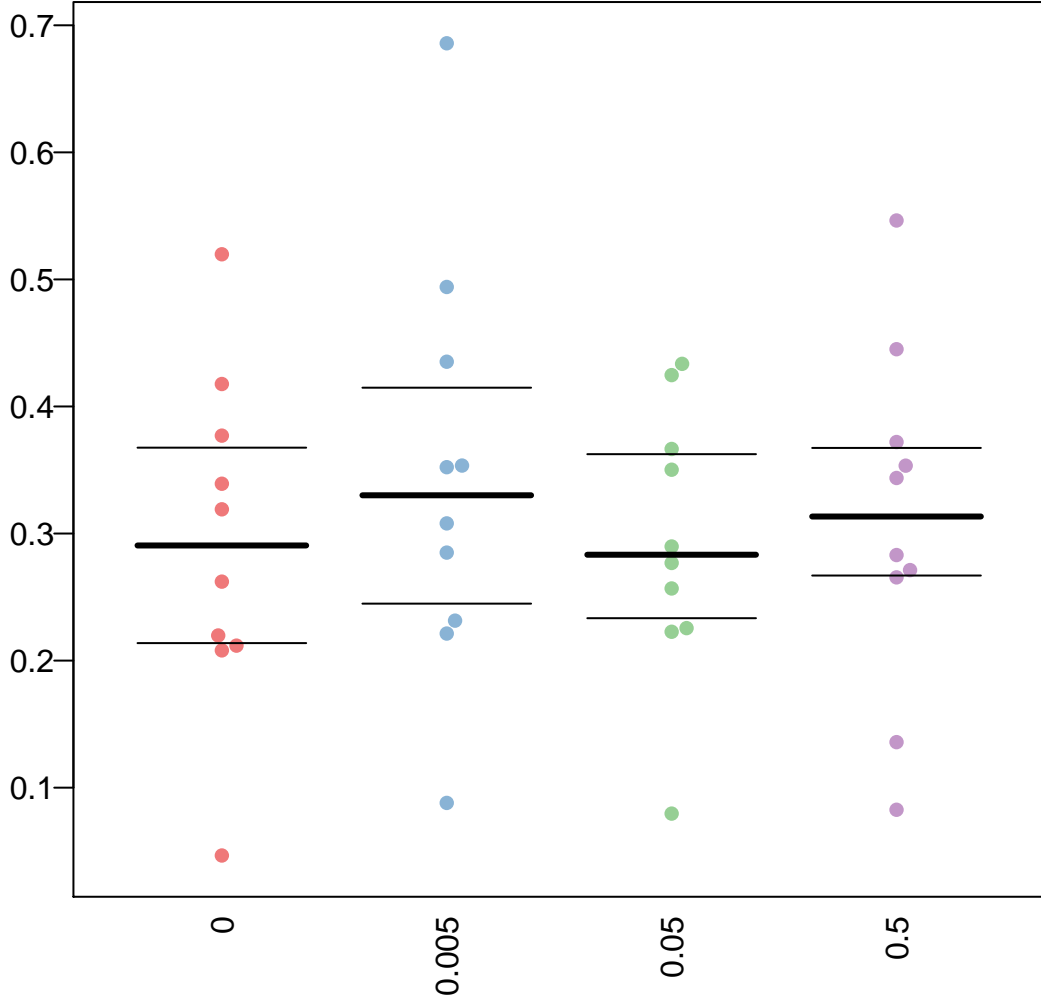

Supplement: Supplementary file 2 [file Data_Sheet_2.zip › predicted_phenotypes_CMC/Gram_Positive.pdf]

Relative Abundance

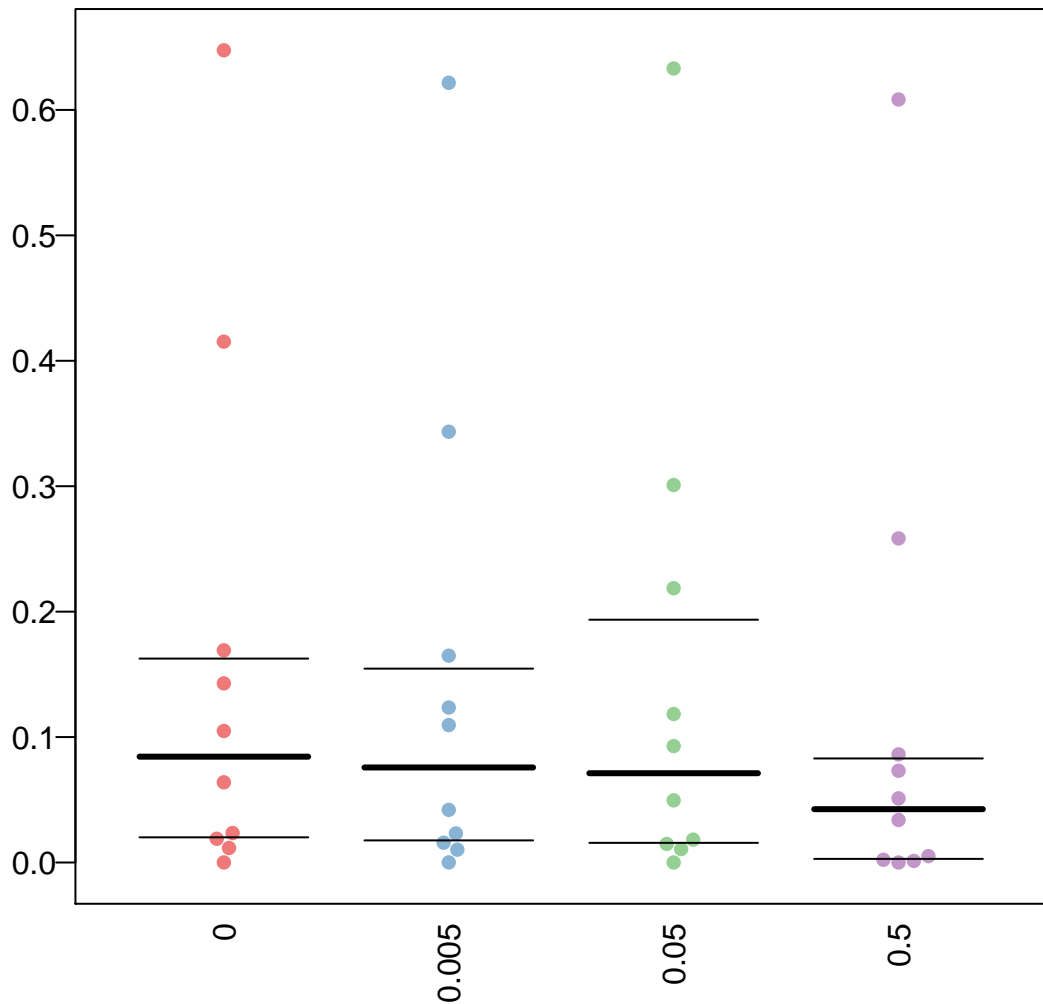

Supplement: Supplementary file 2 [file Data_Sheet_2.zip › predicted_phenotypes_CMC/Potentially_Pathogenic.pdf]

Relative Abundance

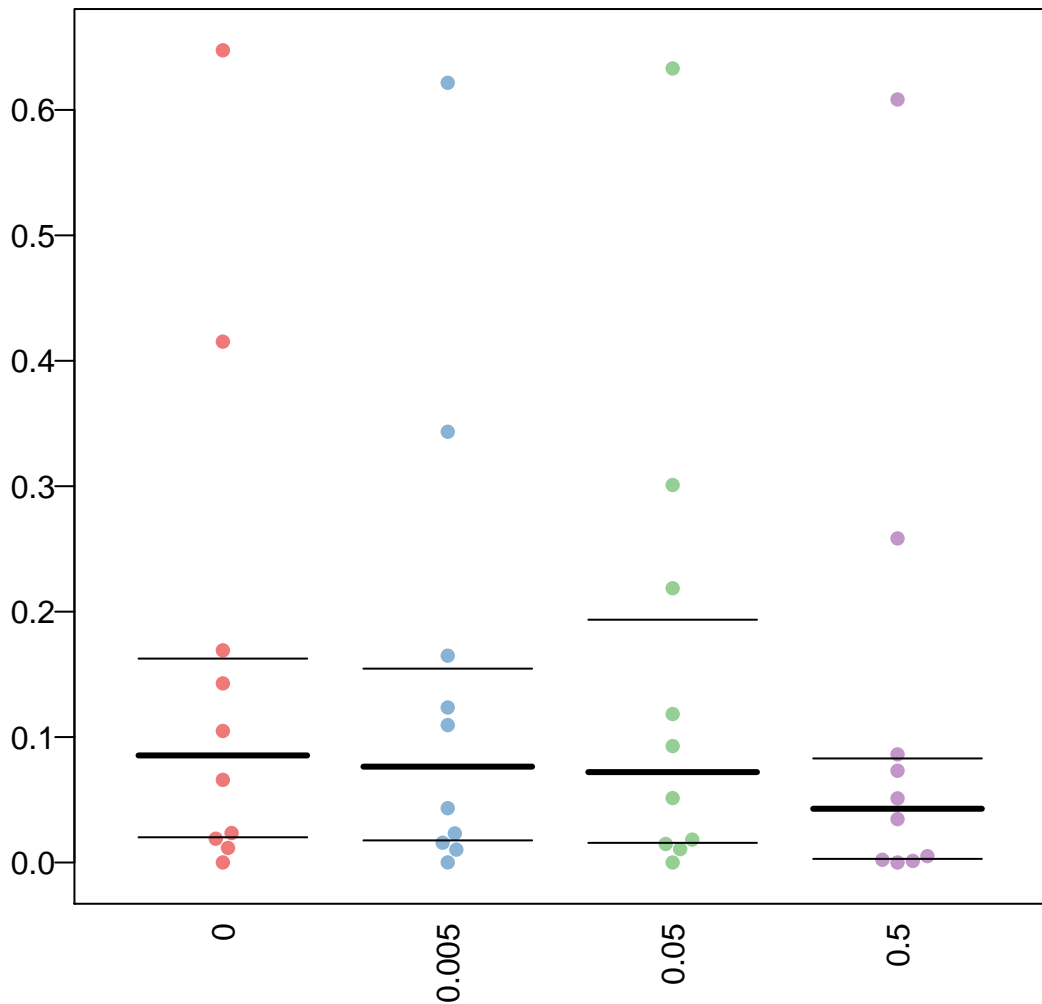

Supplement: Supplementary file 2 [file Data_Sheet_2.zip › predicted_phenotypes_CMC/Stress_Tolerant.pdf]

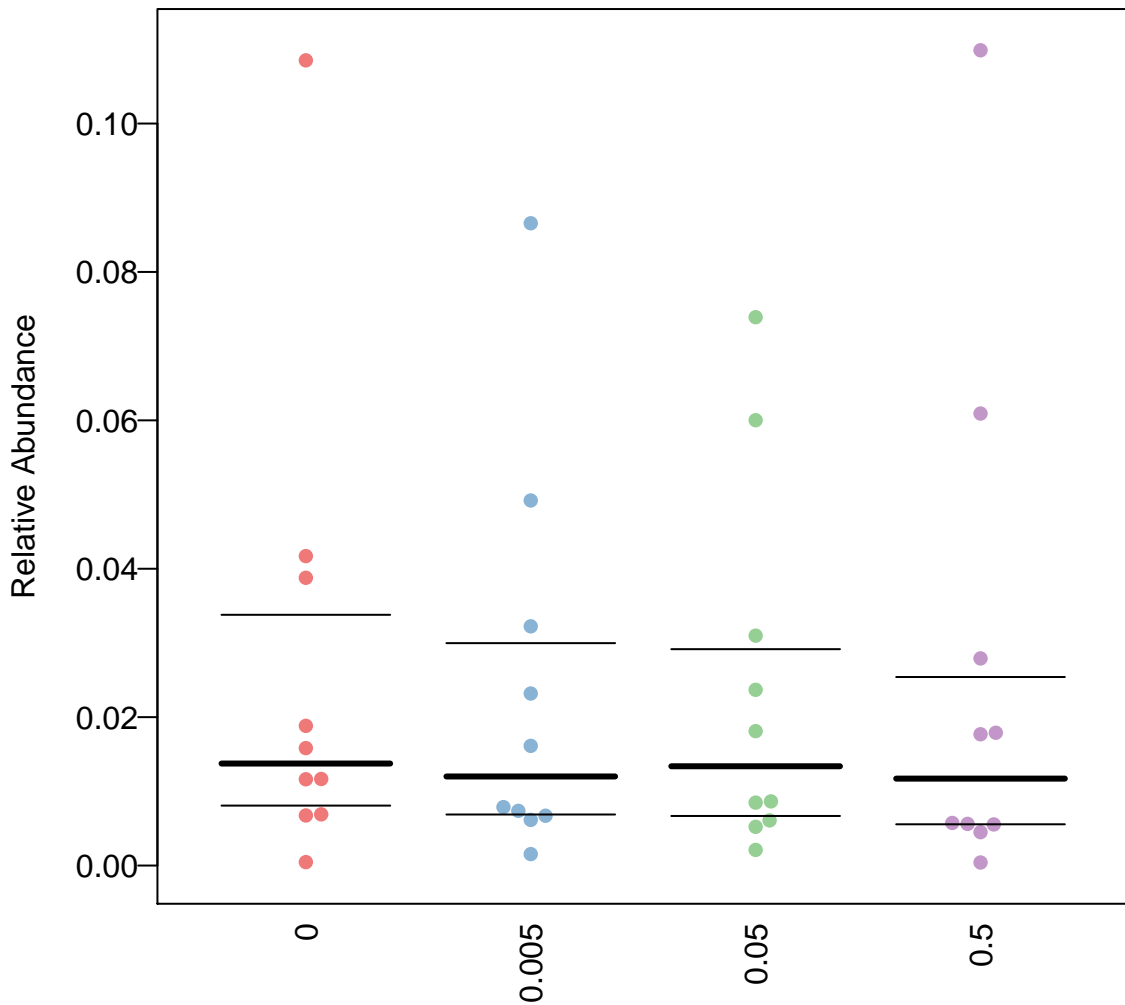

Supplement: Supplementary file 2 [file Data_Sheet_2.zip › predicted_phenotypes_P80/Aerobic.pdf]

Relative Abundance

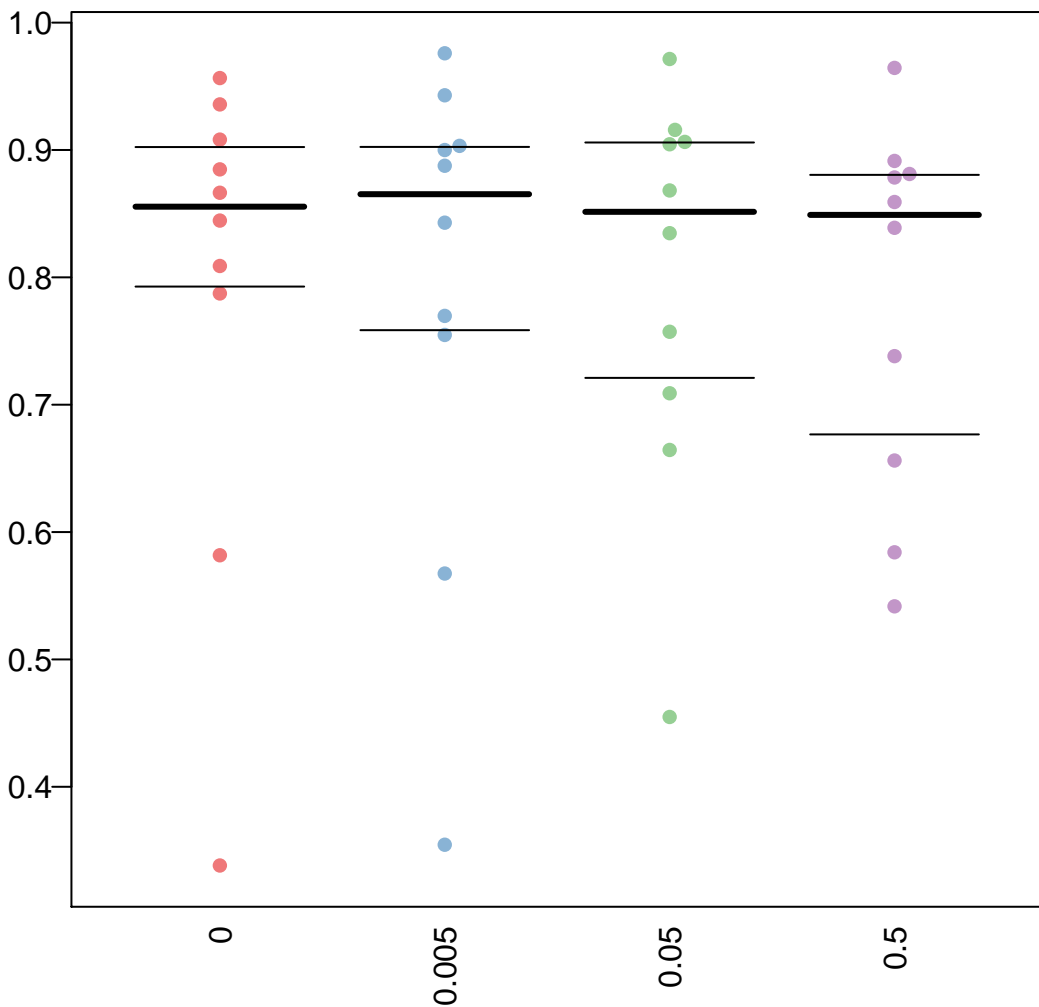

Supplement: Supplementary file 2 [file Data_Sheet_2.zip › predicted_phenotypes_P80/Anaerobic.pdf]

Relative Abundance

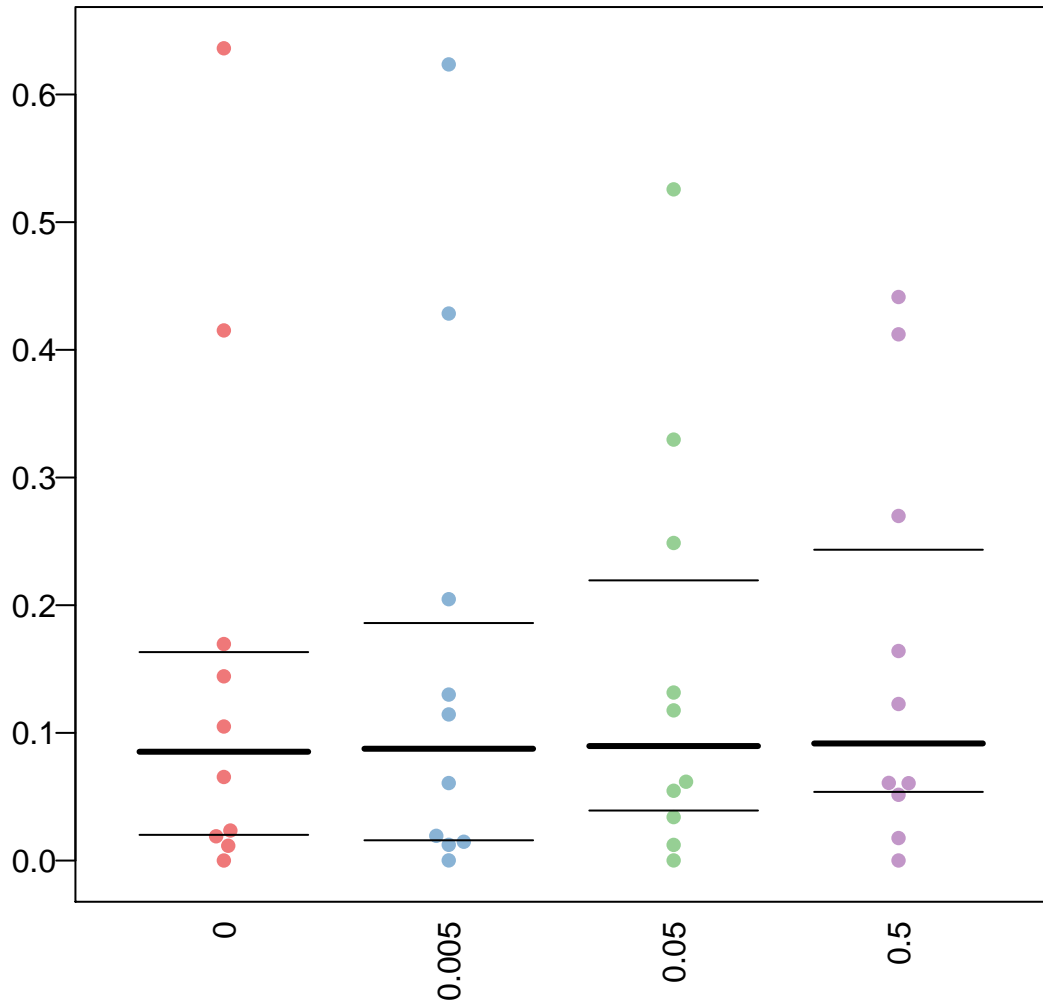

Supplement: Supplementary file 2 [file Data_Sheet_2.zip › predicted_phenotypes_P80/Contains_Mobile_Elements.pdf]

Relative Abundance

0.6  
0.5  
0.4  
0.3  
0.2  
0.1  
0.0

0

0.005

0.05

0.5

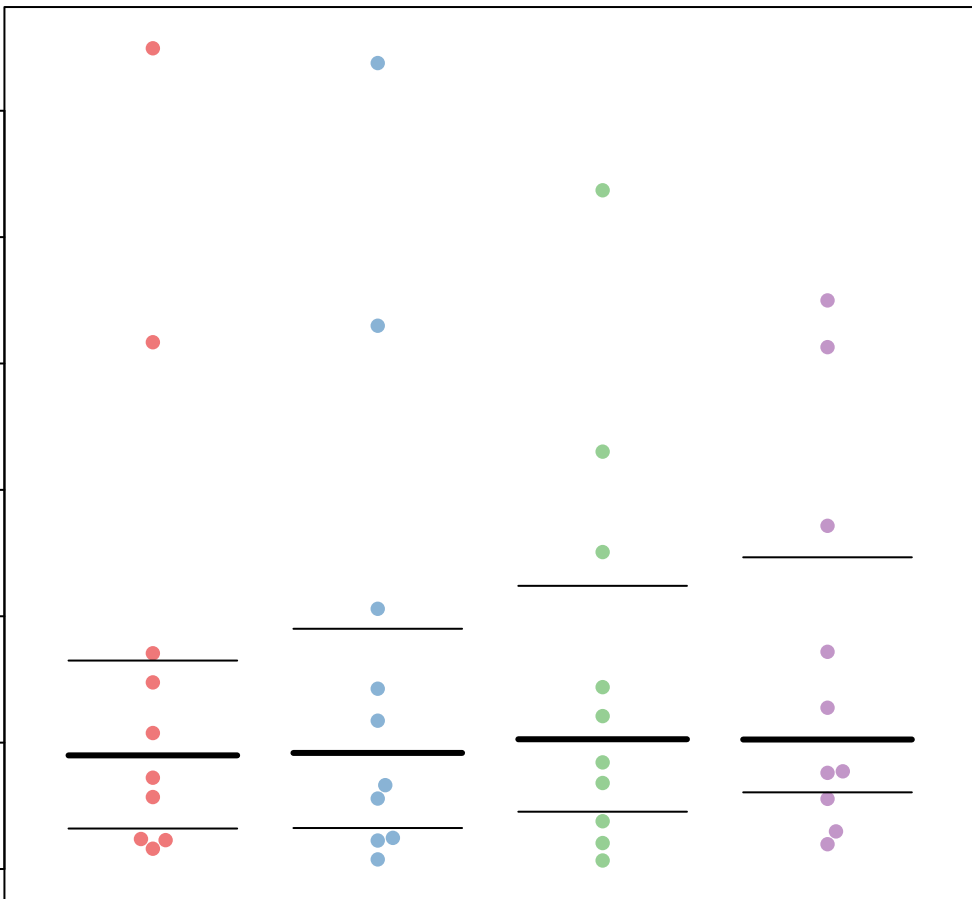

Supplement: Supplementary file 2 [file Data_Sheet_2.zip › predicted_phenotypes_P80/Facultatively_Anaerobic.pdf]

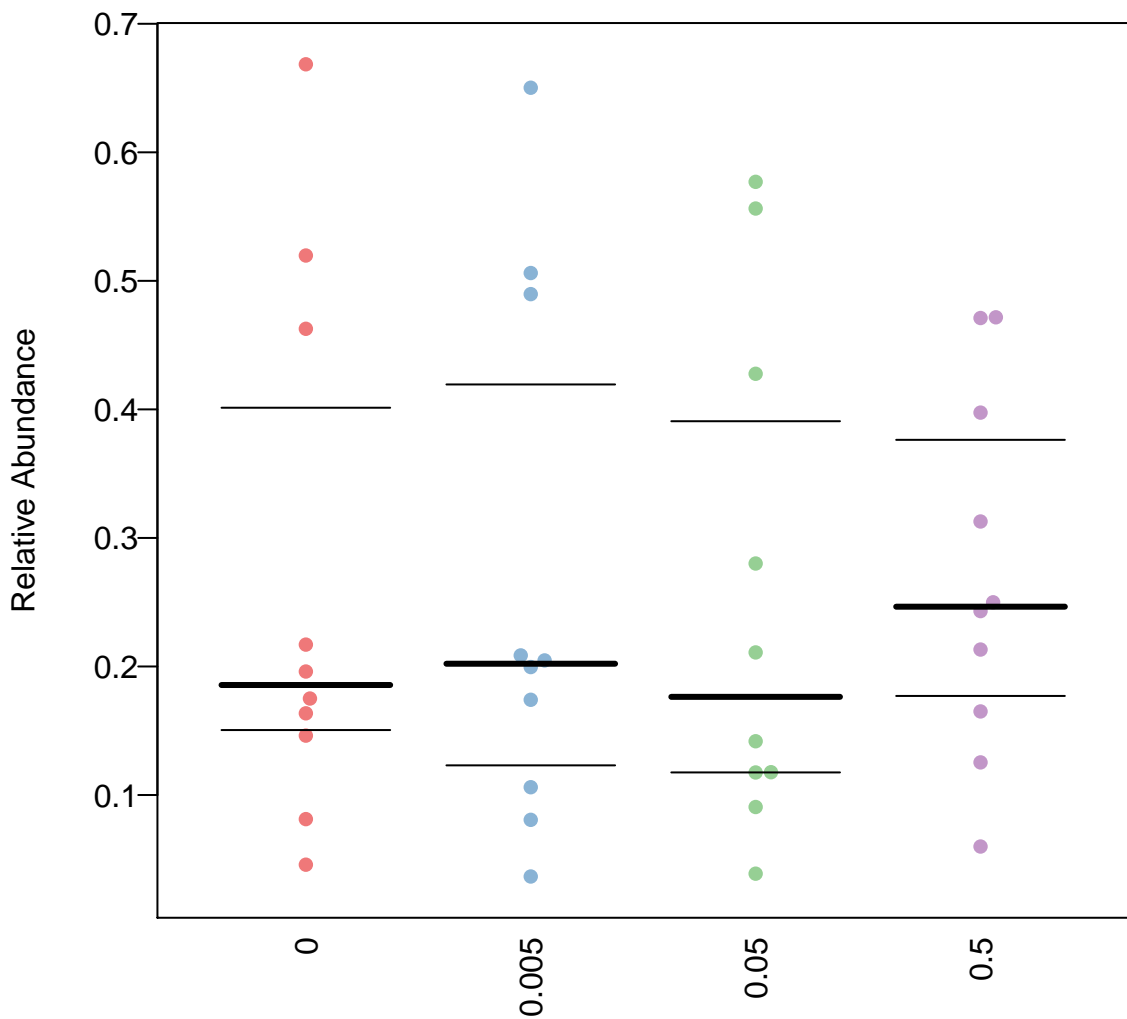

Supplement: Supplementary file 2 [file Data_Sheet_2.zip › predicted_phenotypes_P80/Forms_Biofilms.pdf]

Relative Abundance

0.9  
0.8  
0.7  
0.6  
0.5

0

0.005

0.05

0.5

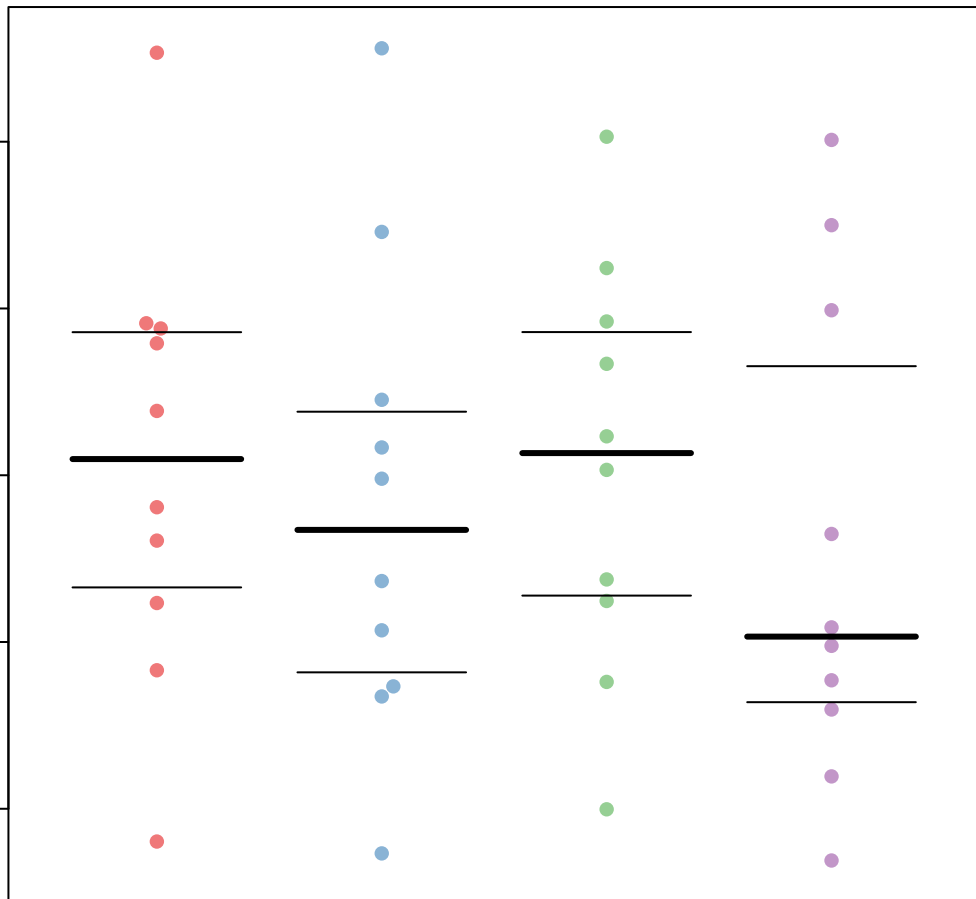

Supplement: Supplementary file 2 [file Data_Sheet_2.zip › predicted_phenotypes_P80/Gram_Negative.pdf]

Relative Abundance

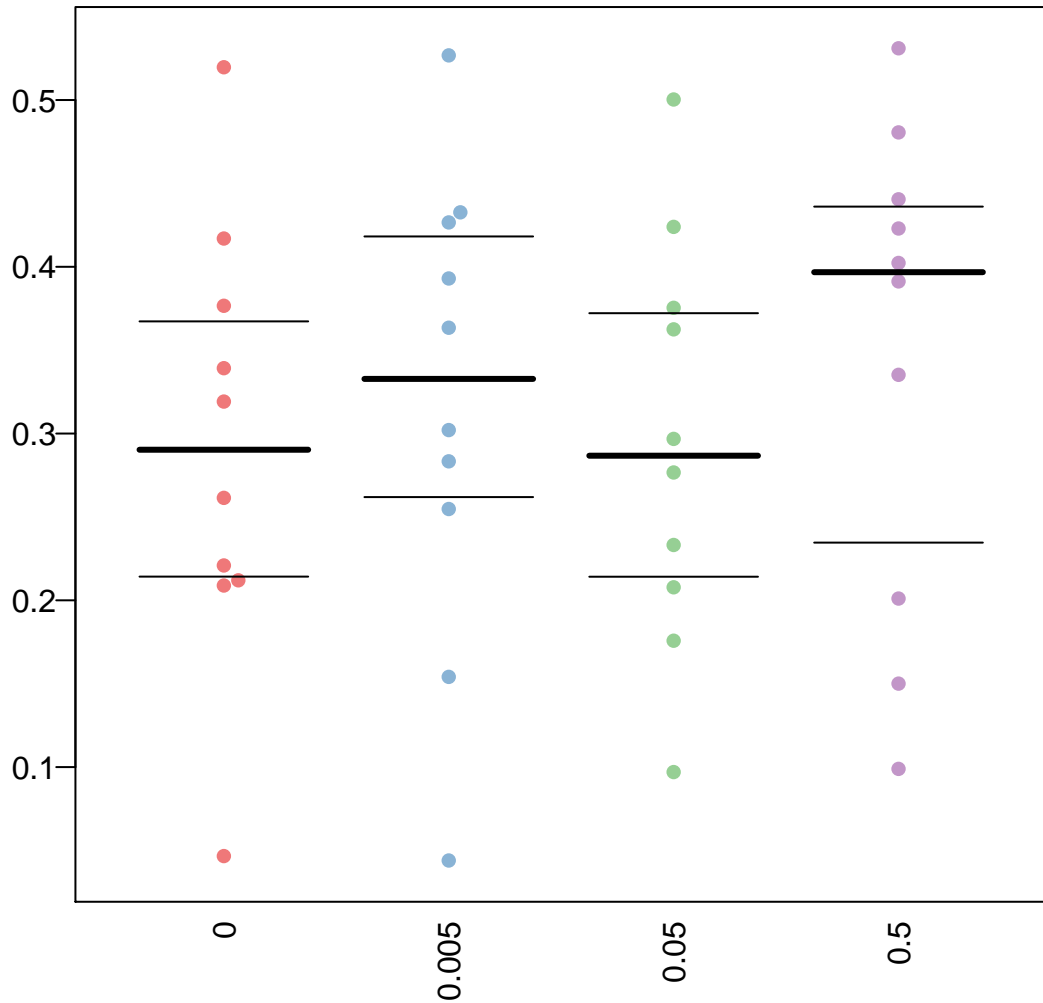

Supplement: Supplementary file 2 [file Data_Sheet_2.zip › predicted_phenotypes_P80/Gram_Positive.pdf]

Relative Abundance

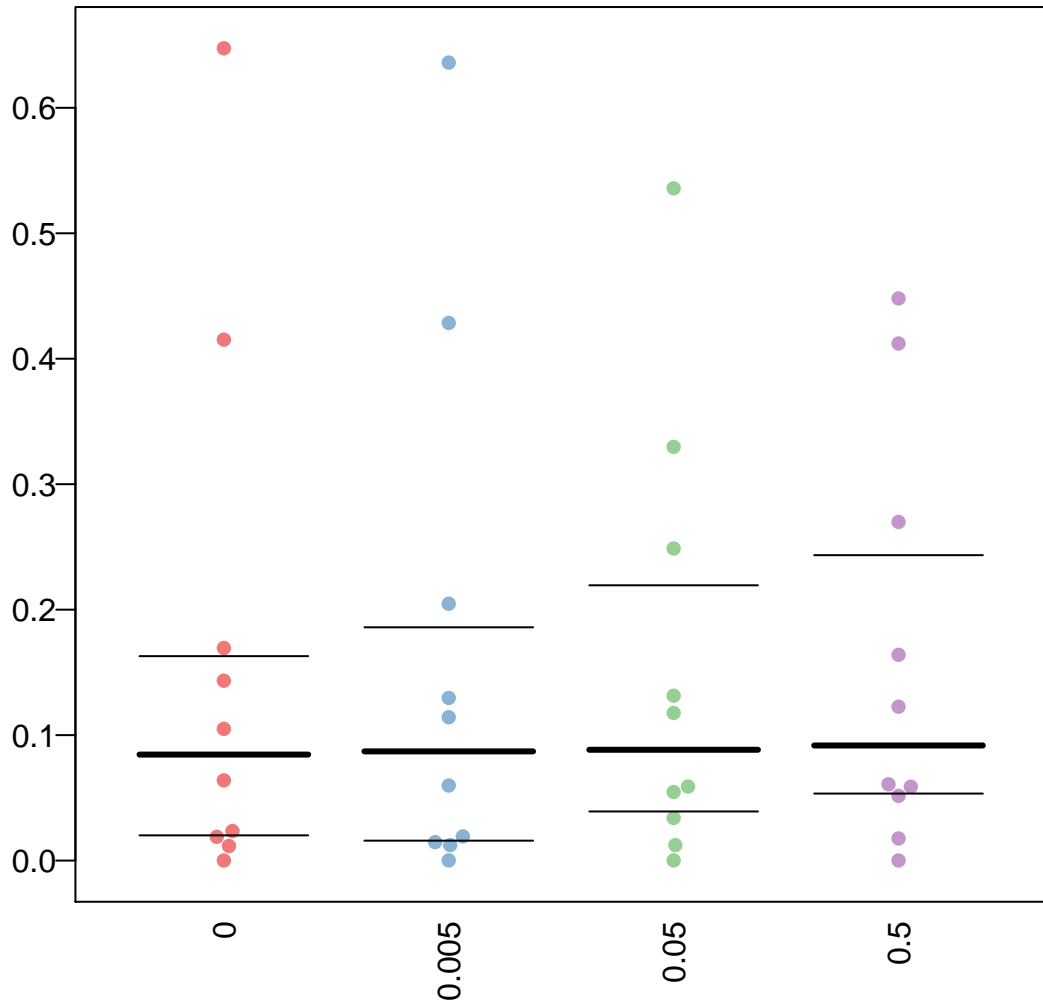

Supplement: Supplementary file 2 [file Data_Sheet_2.zip › predicted_phenotypes_P80/Potentially_Pathogenic.pdf]

Relative Abundance

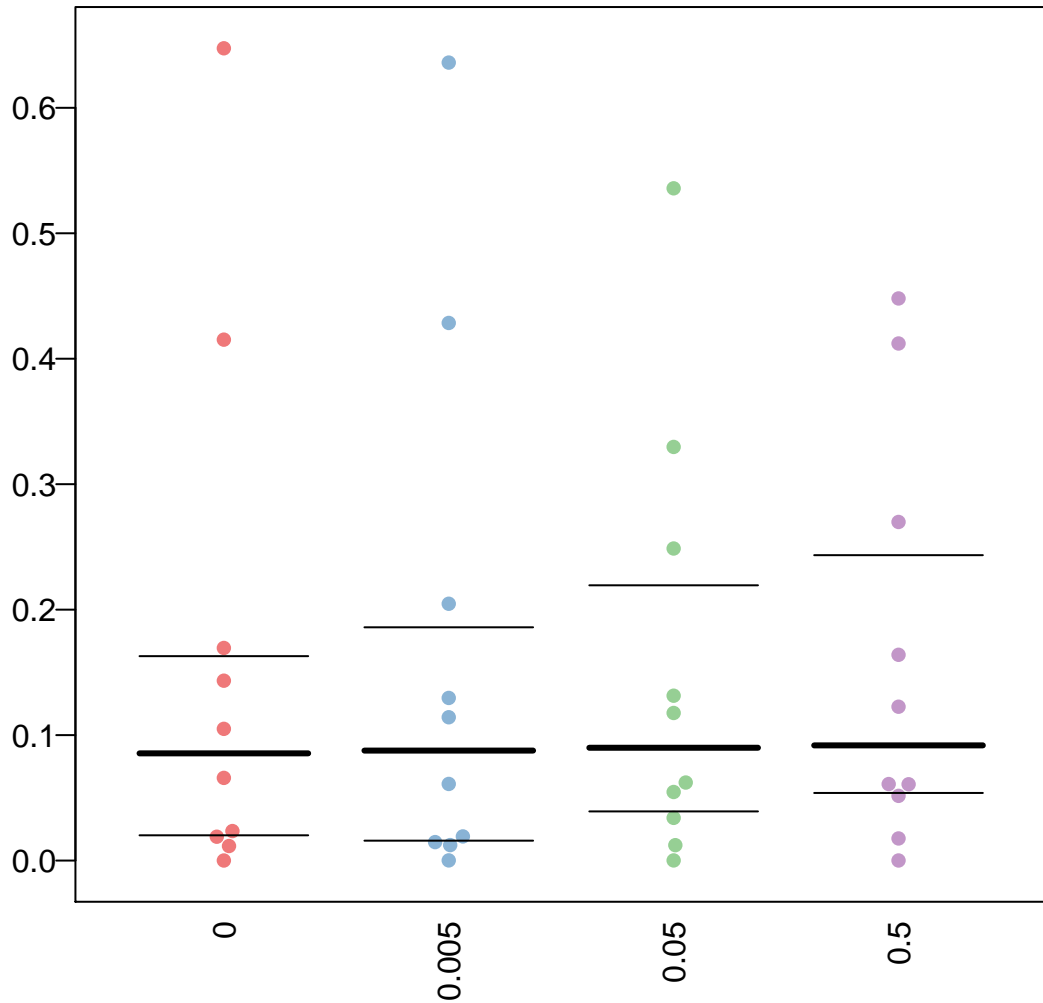

Supplement: Supplementary file 2 [file Data_Sheet_2.zip › predicted_phenotypes_P80/Stress_Tolerant.pdf]

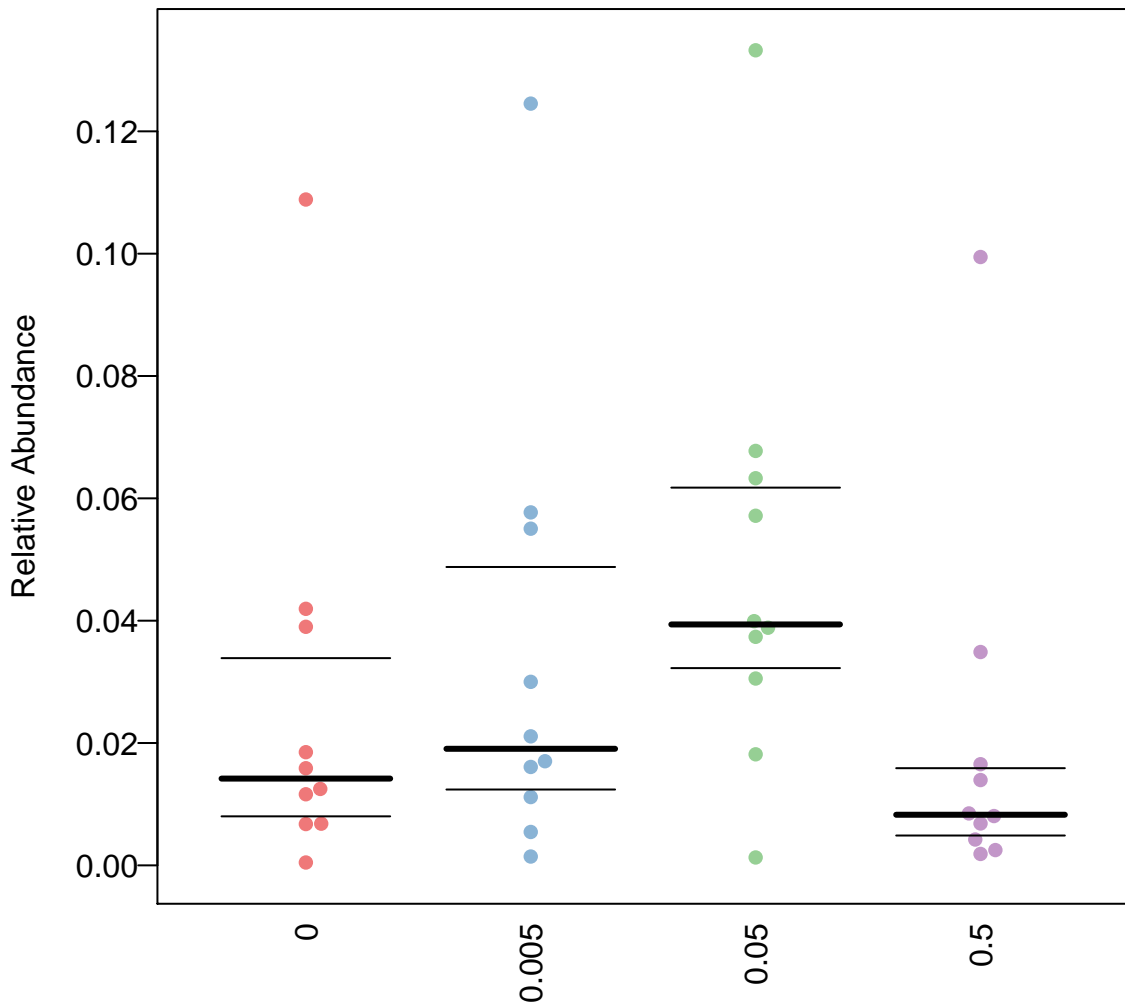

Supplement: Supplementary file 2 [file Data_Sheet_2.zip › predicted_phenotypes_RL/Aerobic.pdf]

Relative Abundance

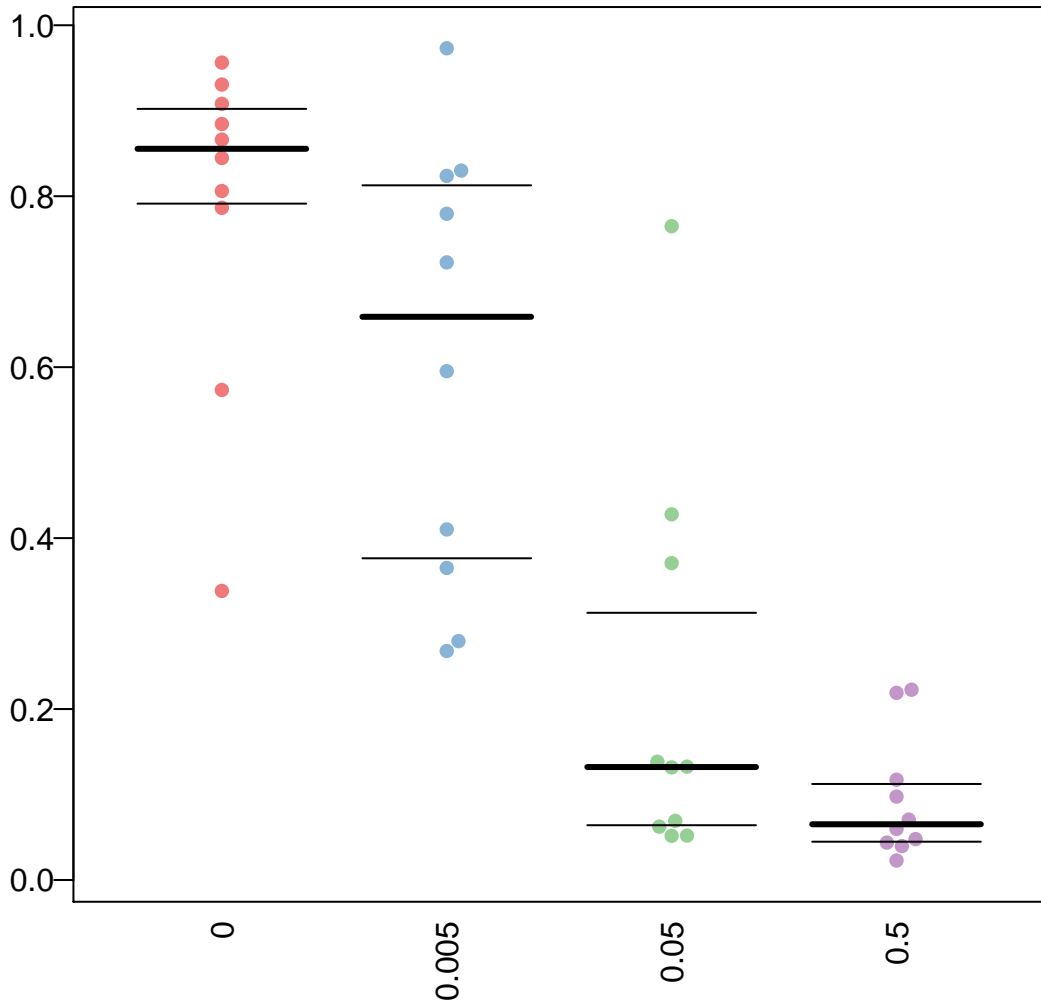

Supplement: Supplementary file 2 [file Data_Sheet_2.zip › predicted_phenotypes_RL/Anaerobic.pdf]

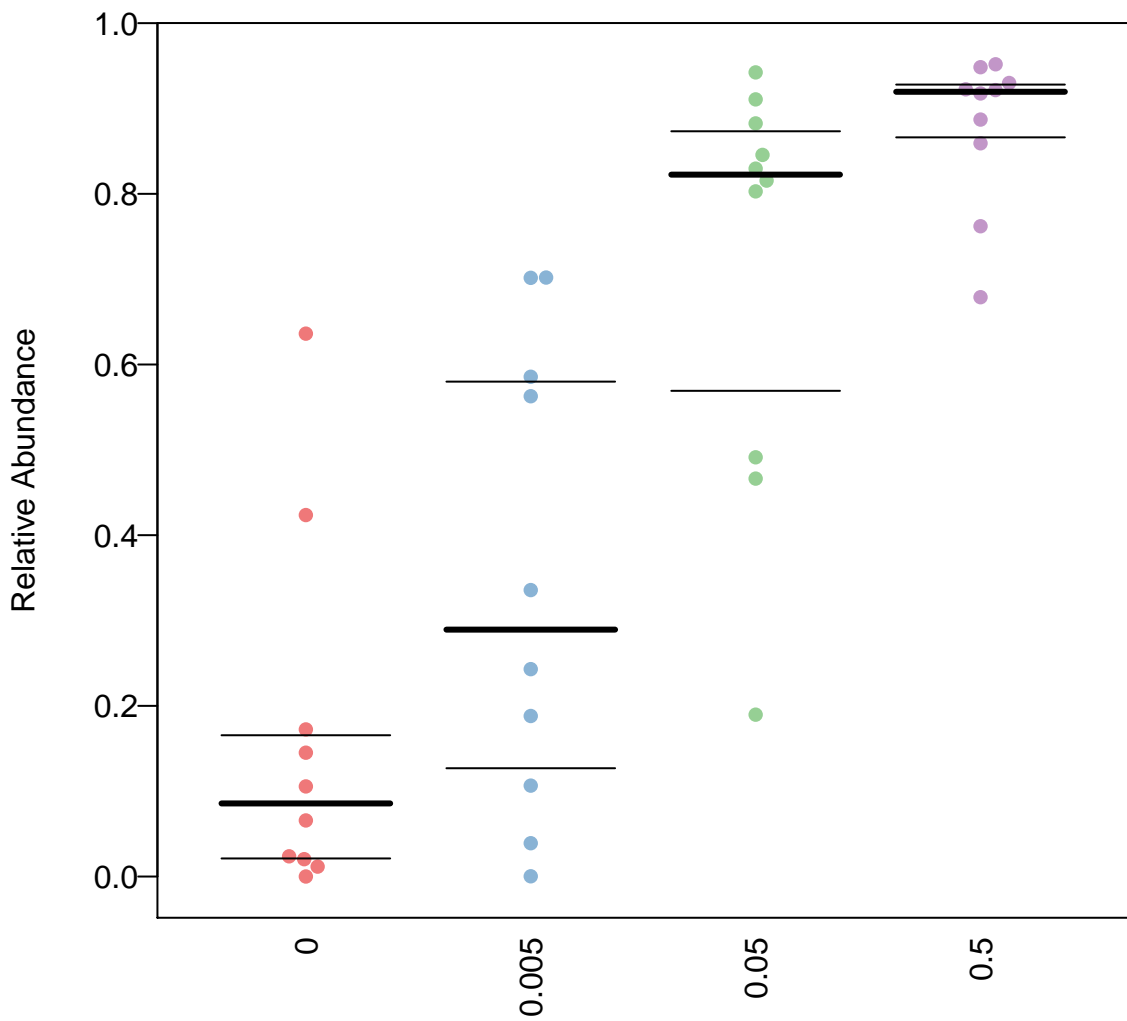

Supplement: Supplementary file 2 [file Data_Sheet_2.zip › predicted_phenotypes_RL/Contains_Mobile_Elements.pdf]

Relative Abundance

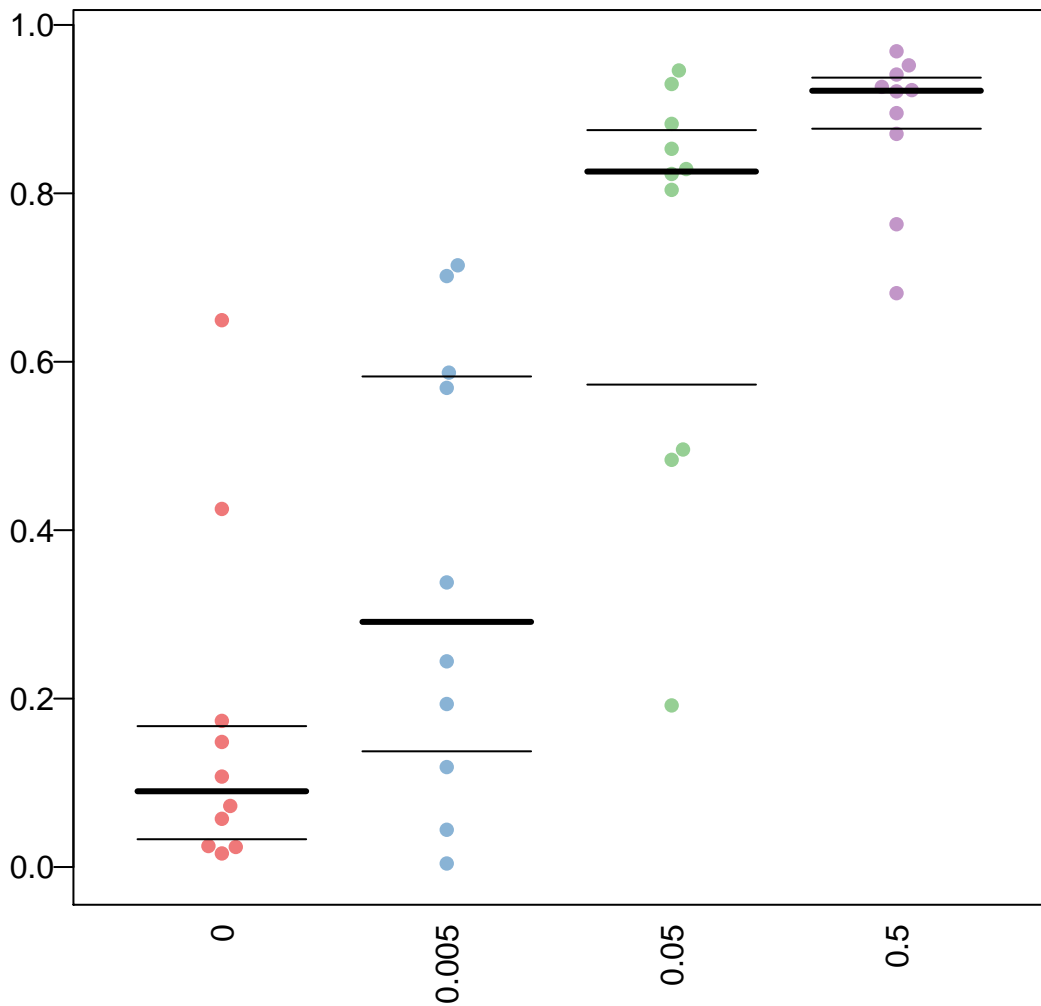

Supplement: Supplementary file 2 [file Data_Sheet_2.zip › predicted_phenotypes_RL/Facultatively_Anaerobic.pdf]

Relative Abundance

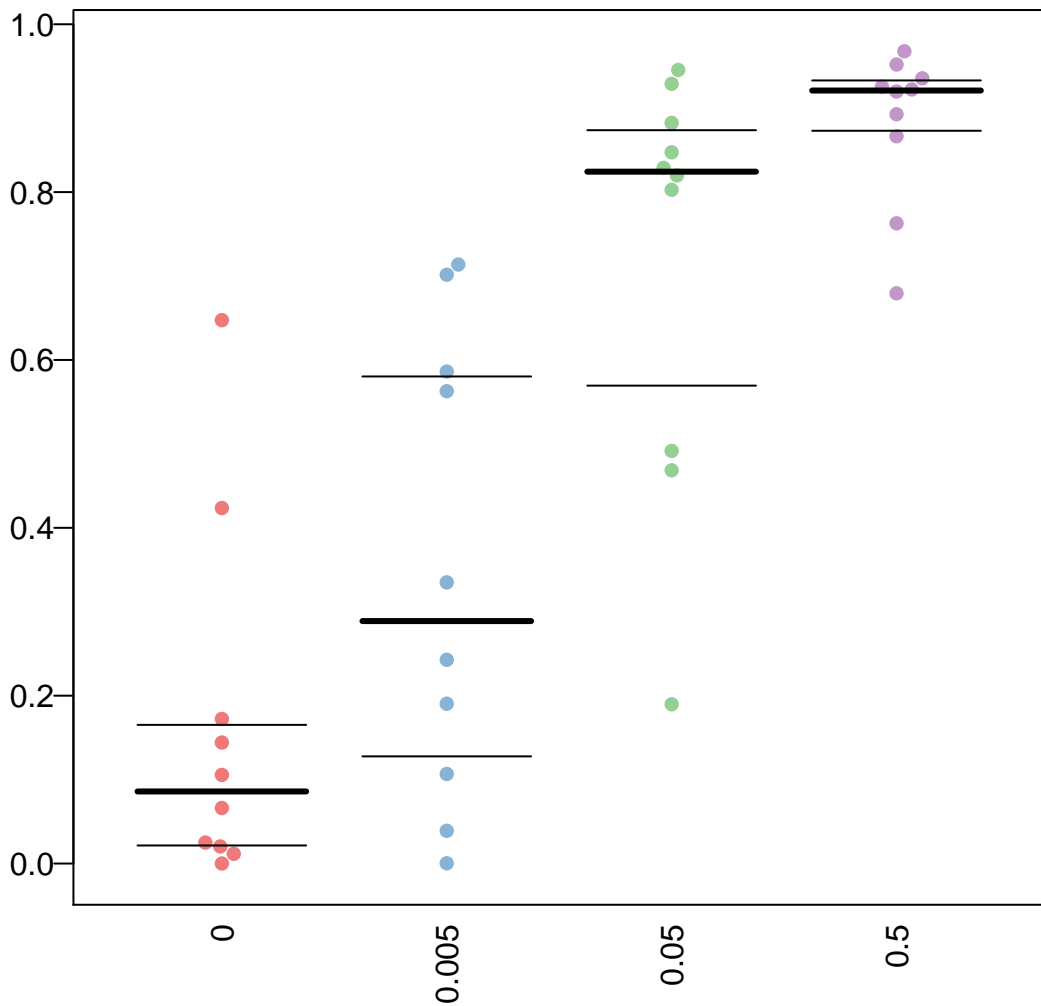

Supplement: Supplementary file 2 [file Data_Sheet_2.zip › predicted_phenotypes_RL/Forms_Biofilms.pdf]

Relative Abundance

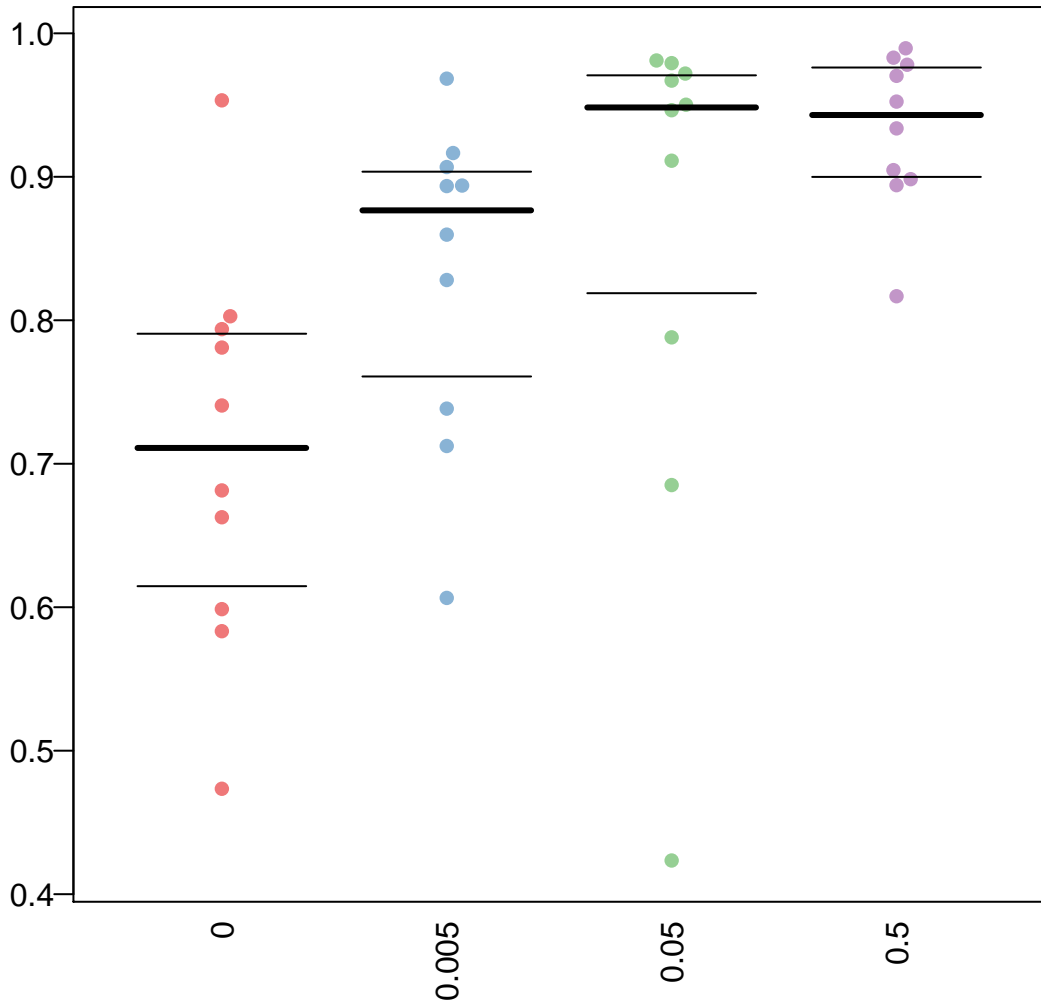

Supplement: Supplementary file 2 [file Data_Sheet_2.zip › predicted_phenotypes_RL/Gram_Negative.pdf]

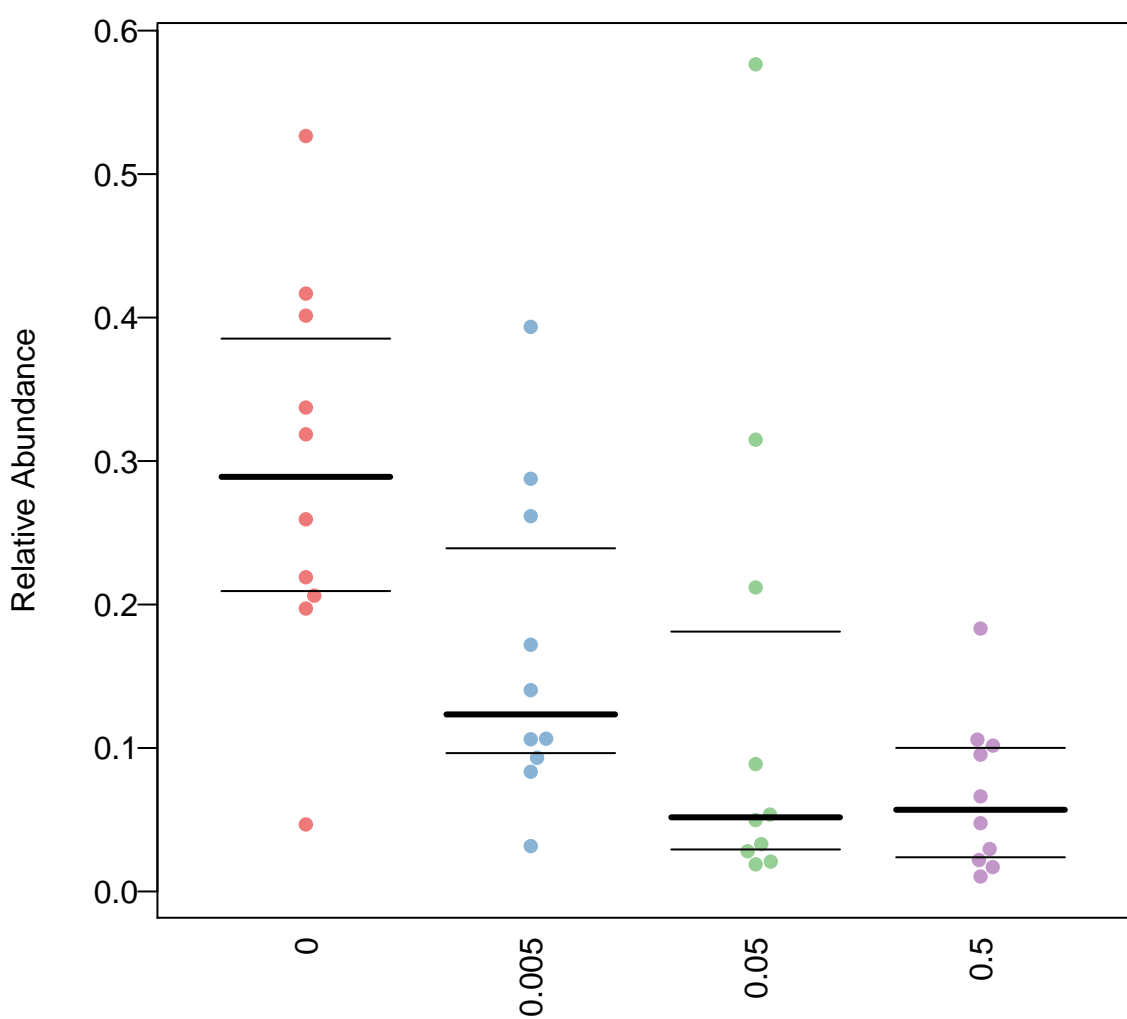

Supplement: Supplementary file 2 [file Data_Sheet_2.zip › predicted_phenotypes_RL/Gram_Positive.pdf]

Relative Abundance

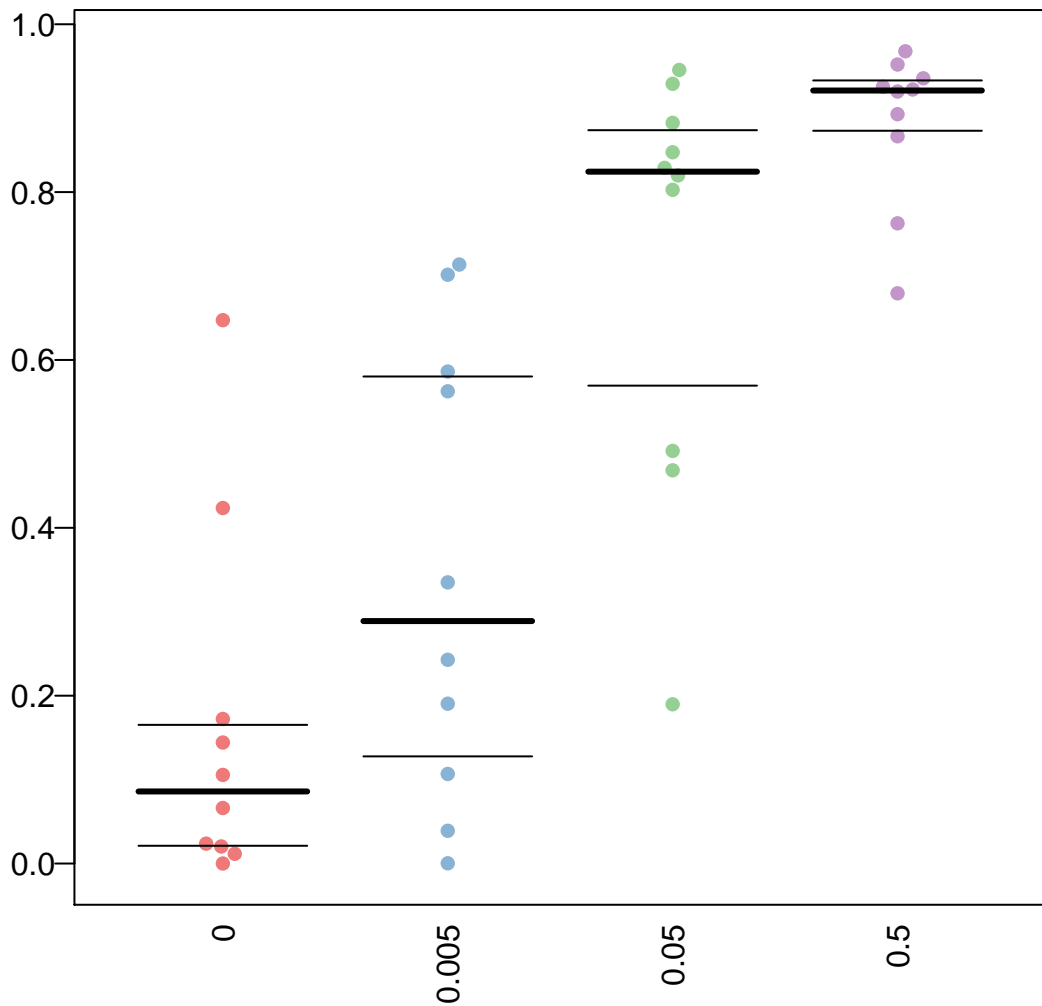

Supplement: Supplementary file 2 [file Data_Sheet_2.zip › predicted_phenotypes_RL/Potentially_Pathogenic.pdf]

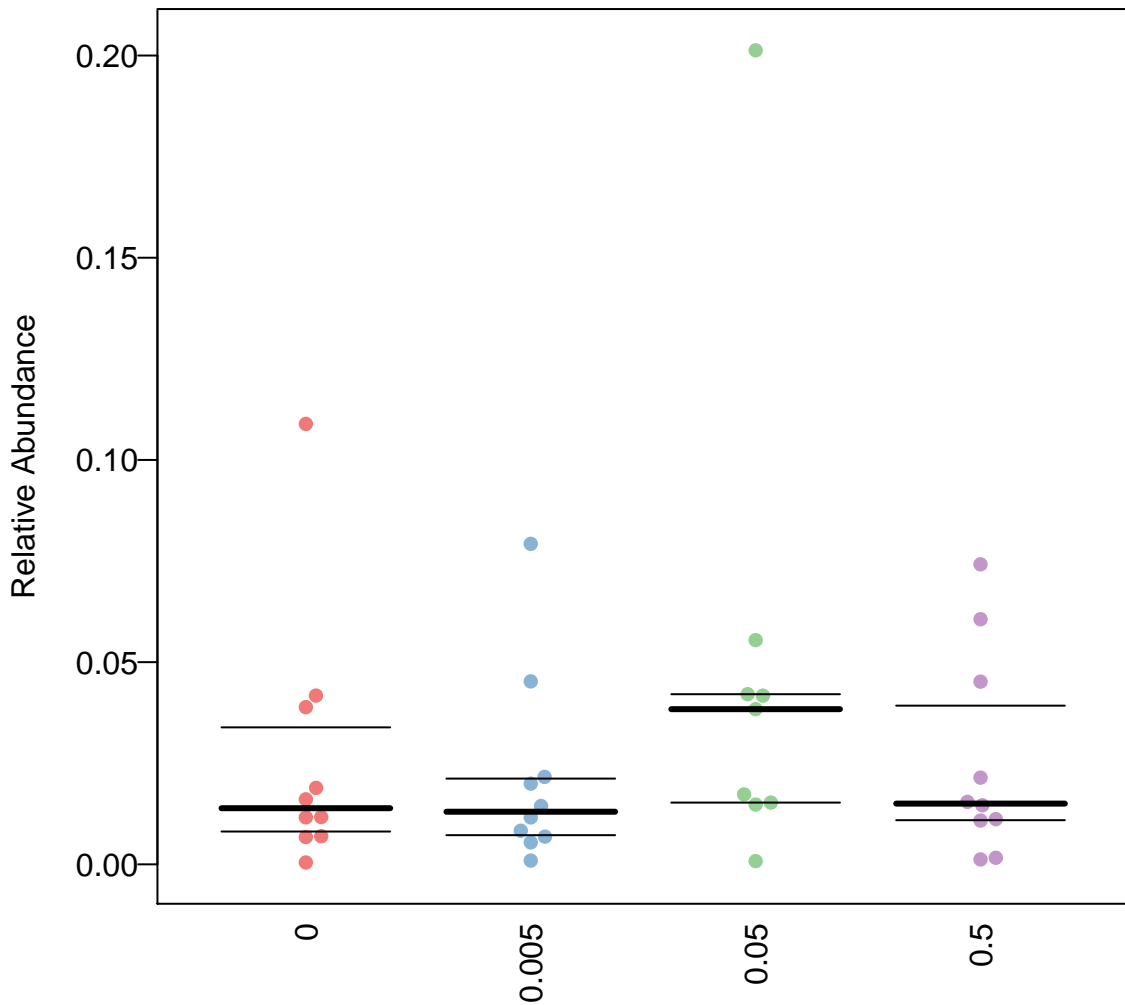

Supplement: Supplementary file 2 [file Data_Sheet_2.zip › predicted_phenotypes_SL/Aerobic.pdf]

Relative Abundance

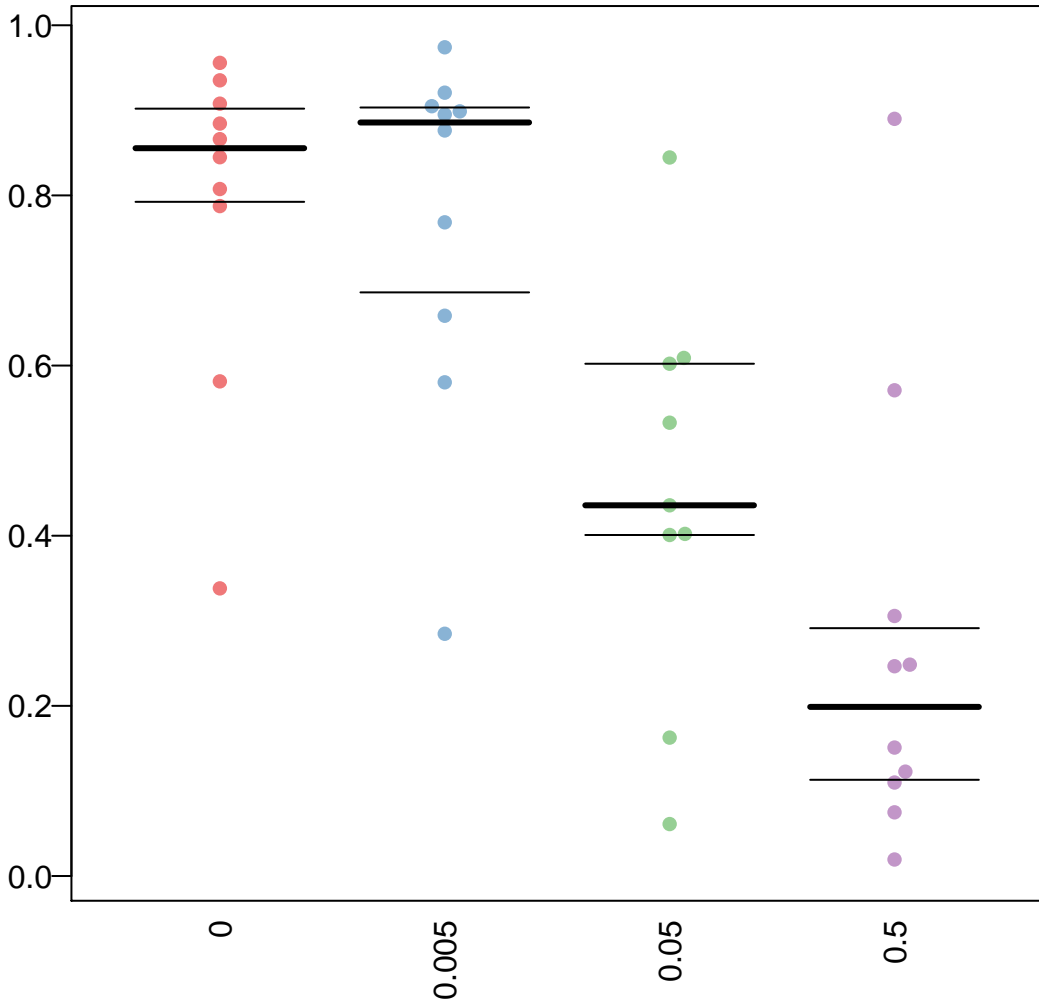

Supplement: Supplementary file 2 [file Data_Sheet_2.zip › predicted_phenotypes_SL/Anaerobic.pdf]

Relative Abundance

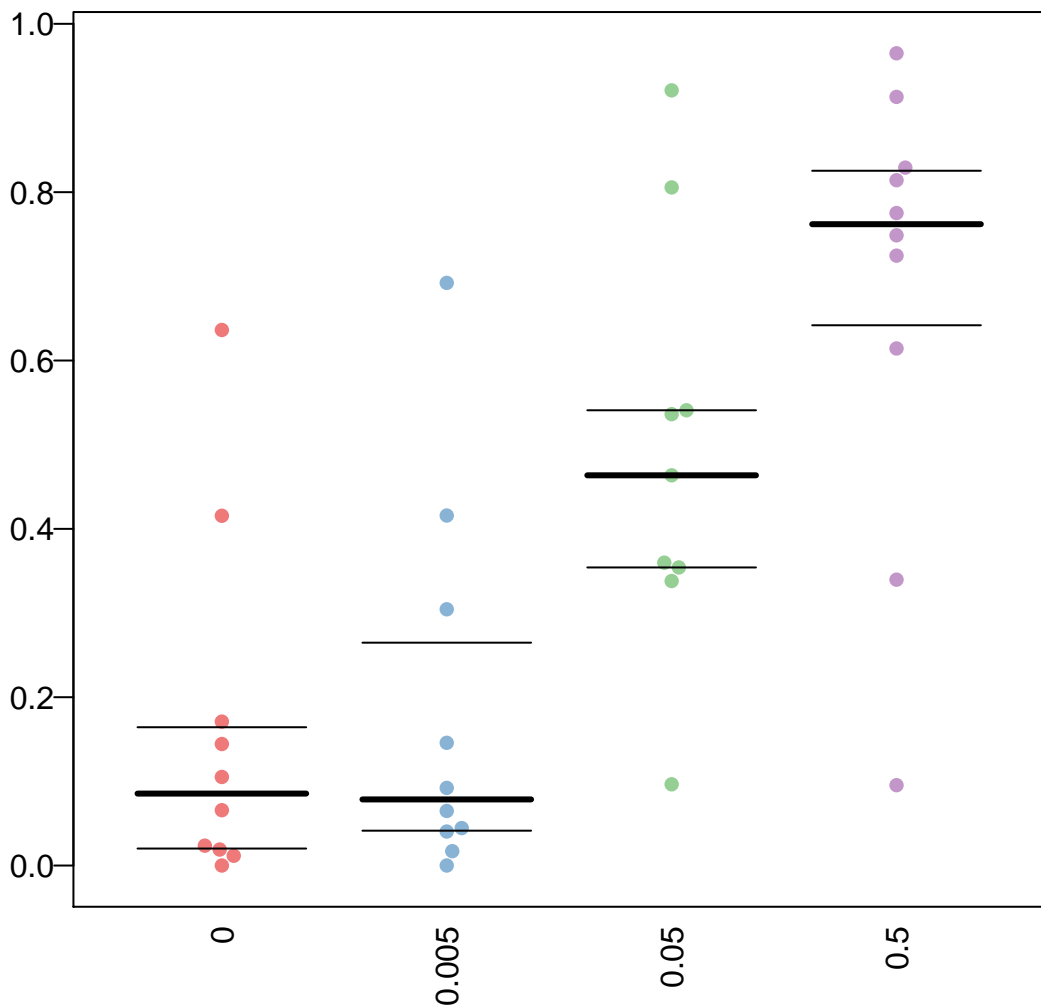

Supplement: Supplementary file 2 [file Data_Sheet_2.zip › predicted_phenotypes_SL/Contains_Mobile_Elements.pdf]

Relative Abundance

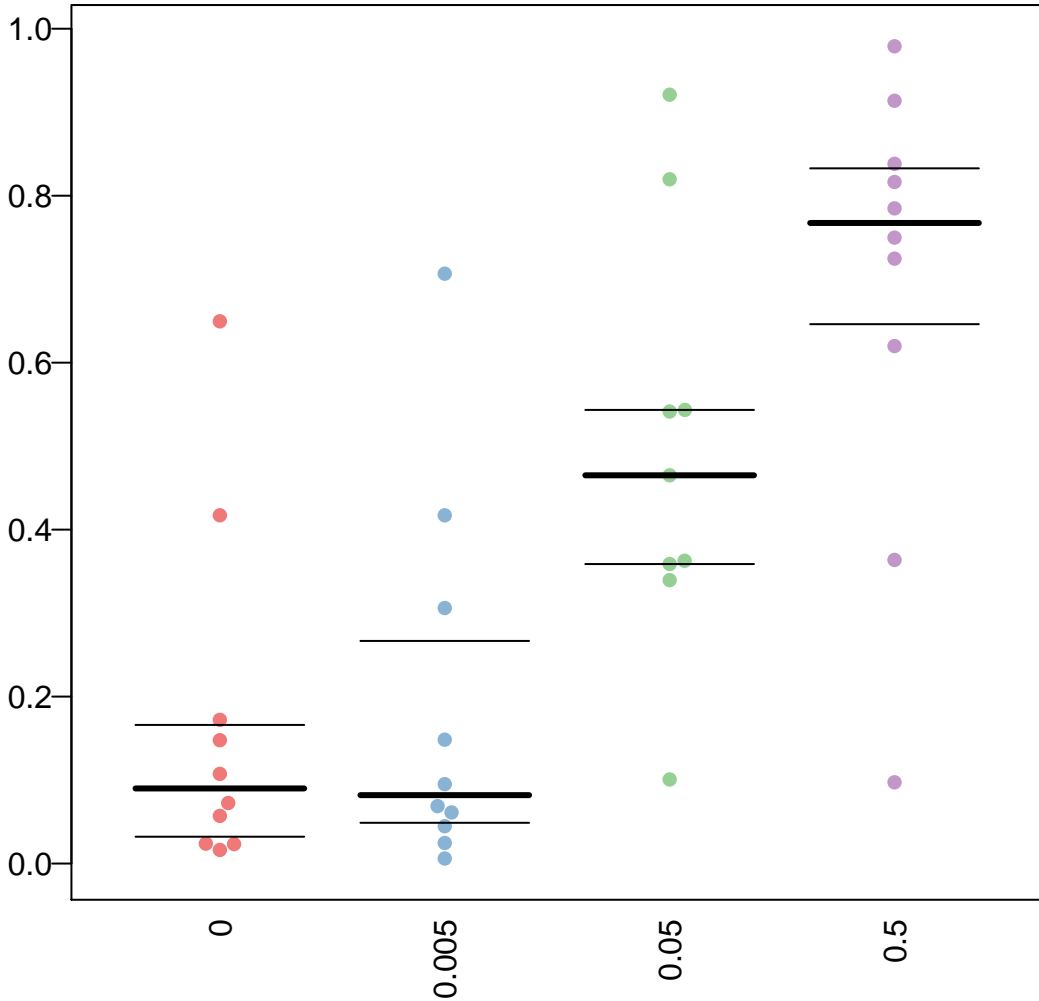

Supplement: Supplementary file 2 [file Data_Sheet_2.zip › predicted_phenotypes_SL/Facultatively_Anaerobic.pdf]

Relative Abundance

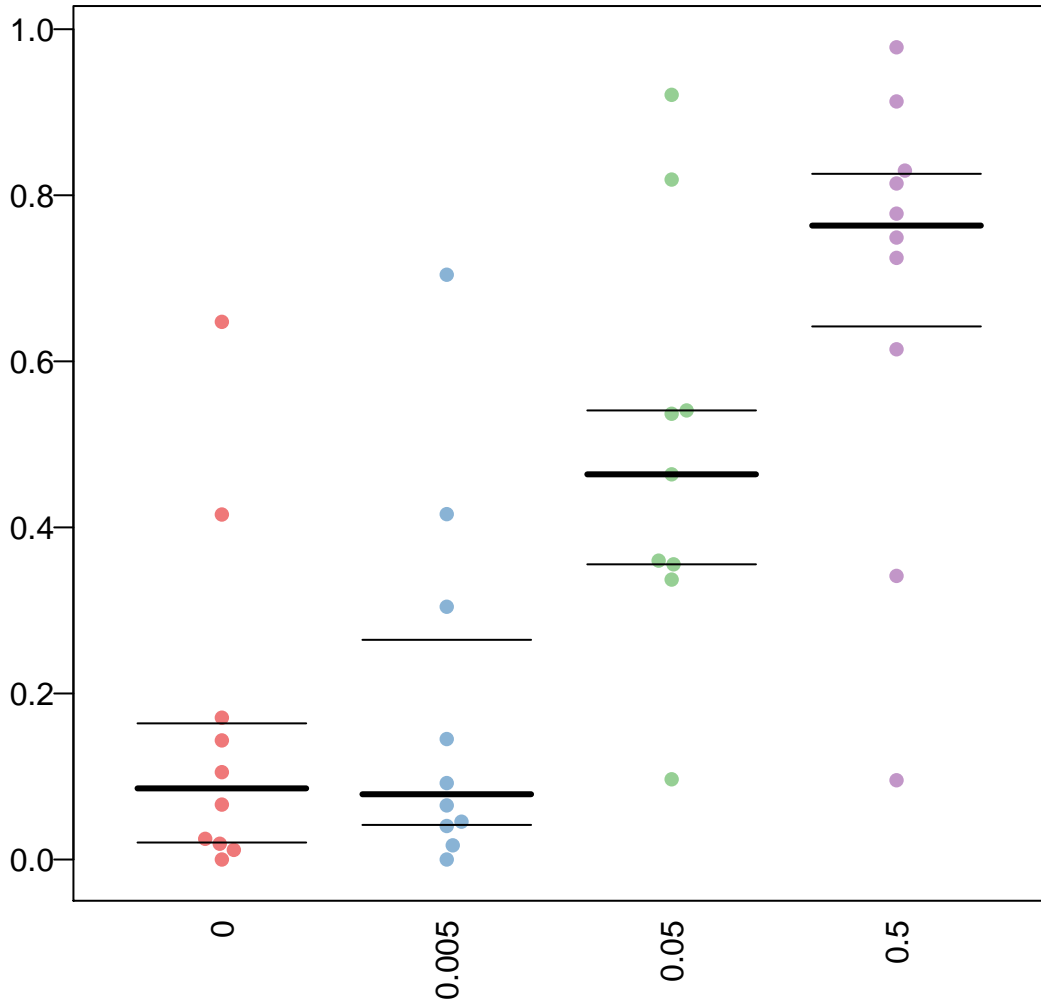

Supplement: Supplementary file 2 [file Data_Sheet_2.zip › predicted_phenotypes_SL/Forms_Biofilms.pdf]

Relative Abundance

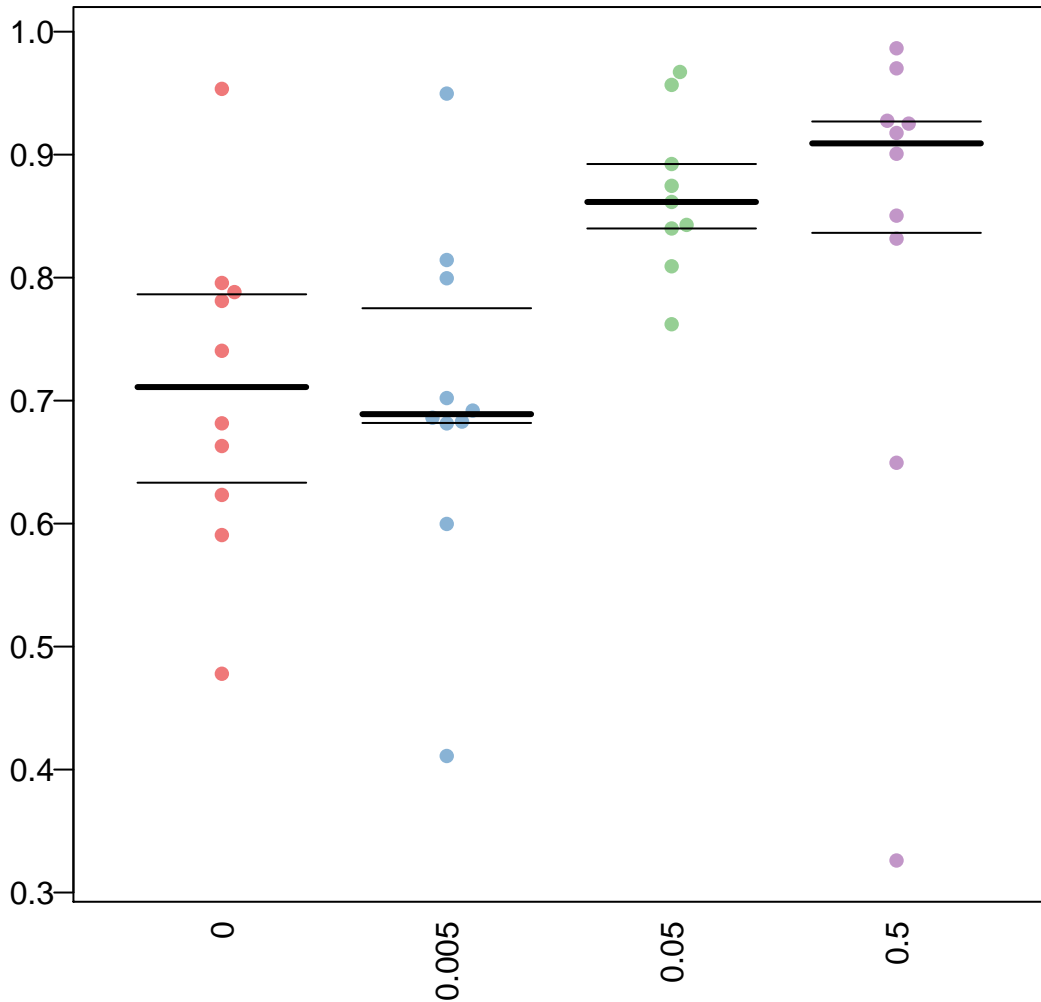

Supplement: Supplementary file 2 [file Data_Sheet_2.zip › predicted_phenotypes_SL/Gram_Negative.pdf]

Relative Abundance

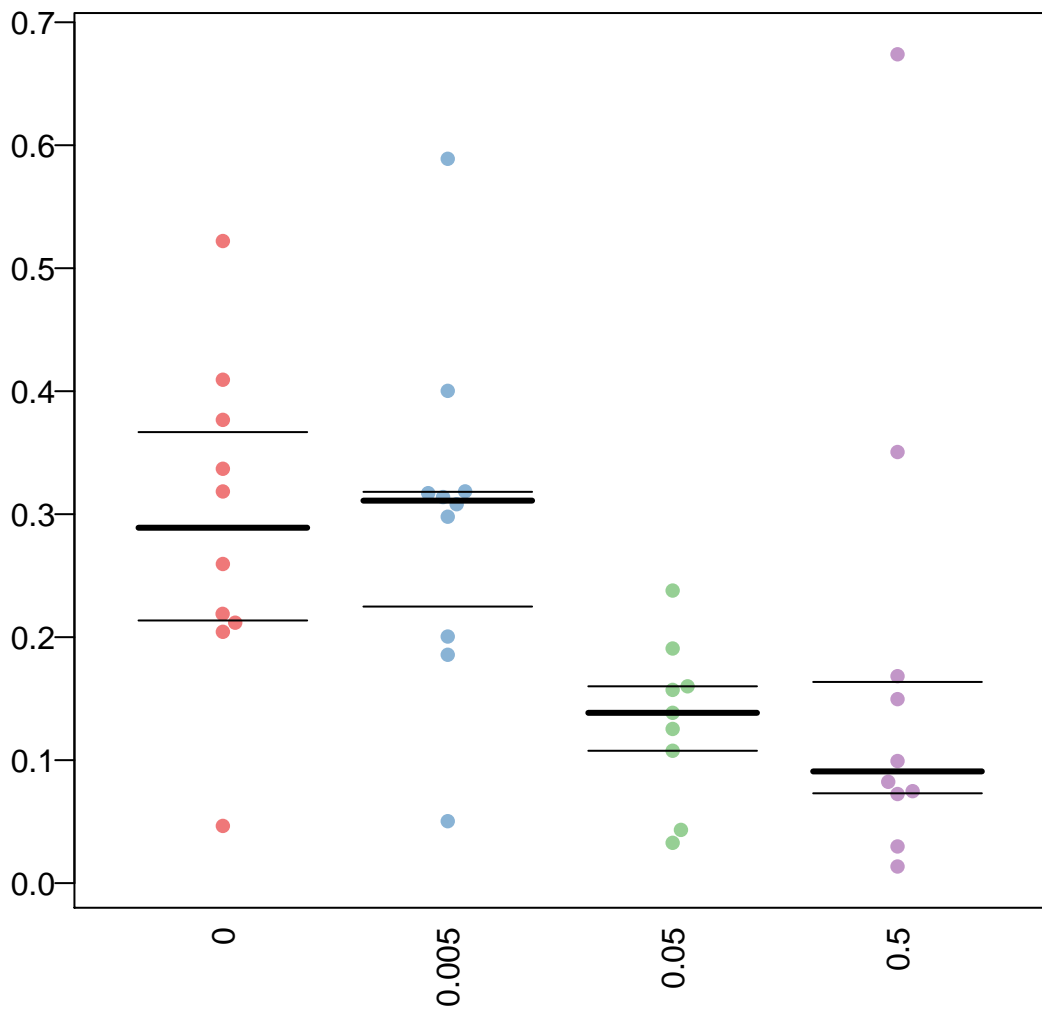

Supplement: Supplementary file 2 [file Data_Sheet_2.zip › predicted_phenotypes_SL/Gram_Positive.pdf]

Relative Abundance

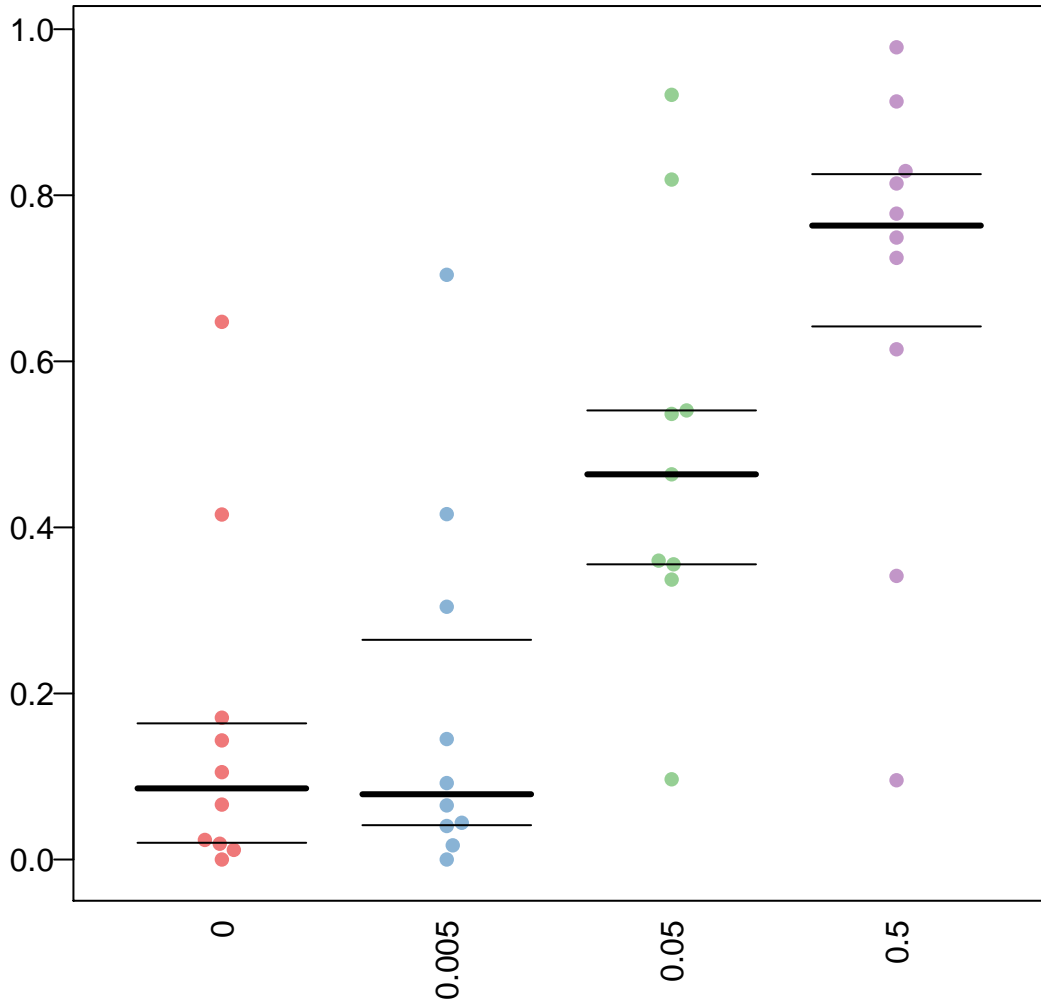

Supplement: Supplementary file 2 [file Data_Sheet_2.zip › predicted_phenotypes_SL/Potentially_Pathogenic.pdf]

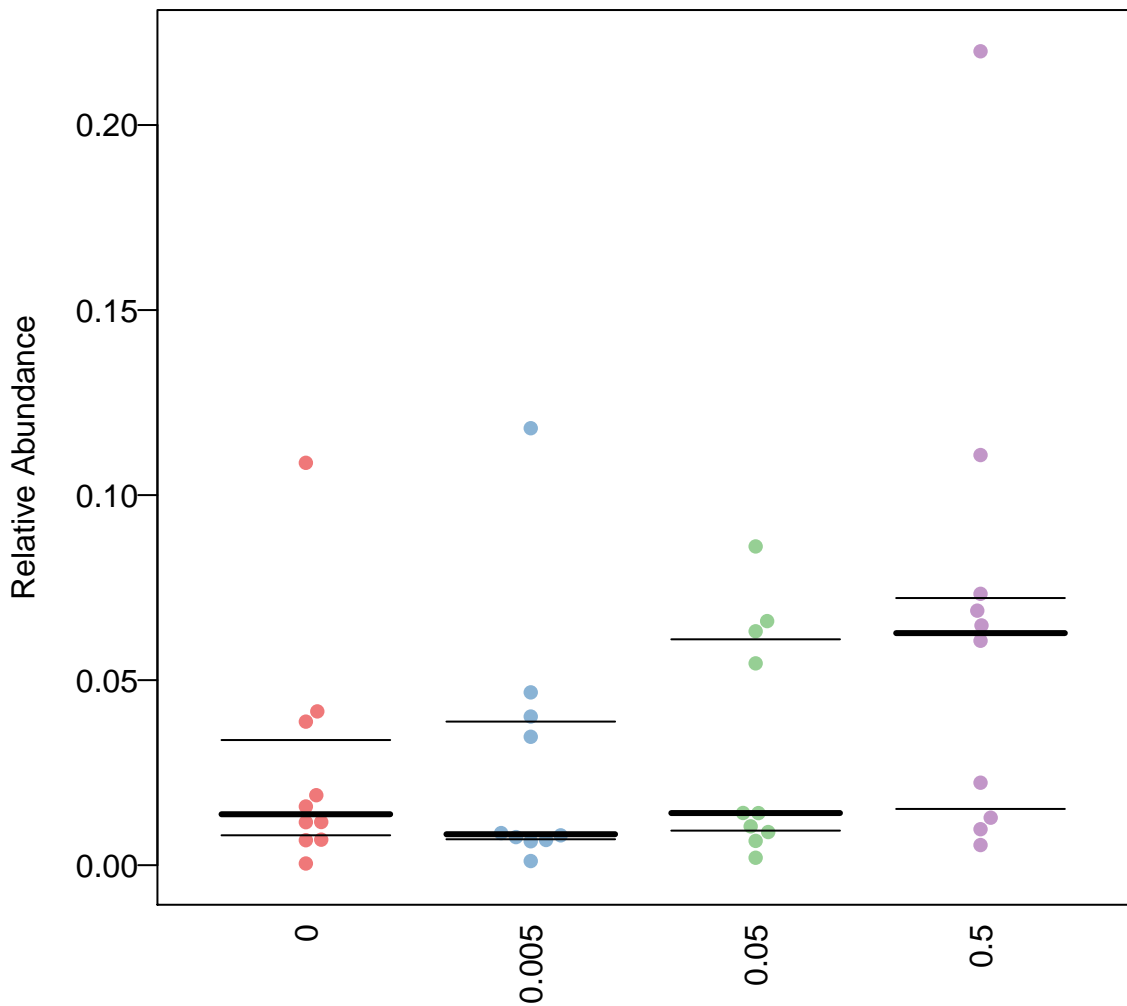

Supplement: Supplementary file 2 [file Data_Sheet_2.zip › predicted_phenotypes_SoyL/Aerobic.pdf]

Relative Abundance

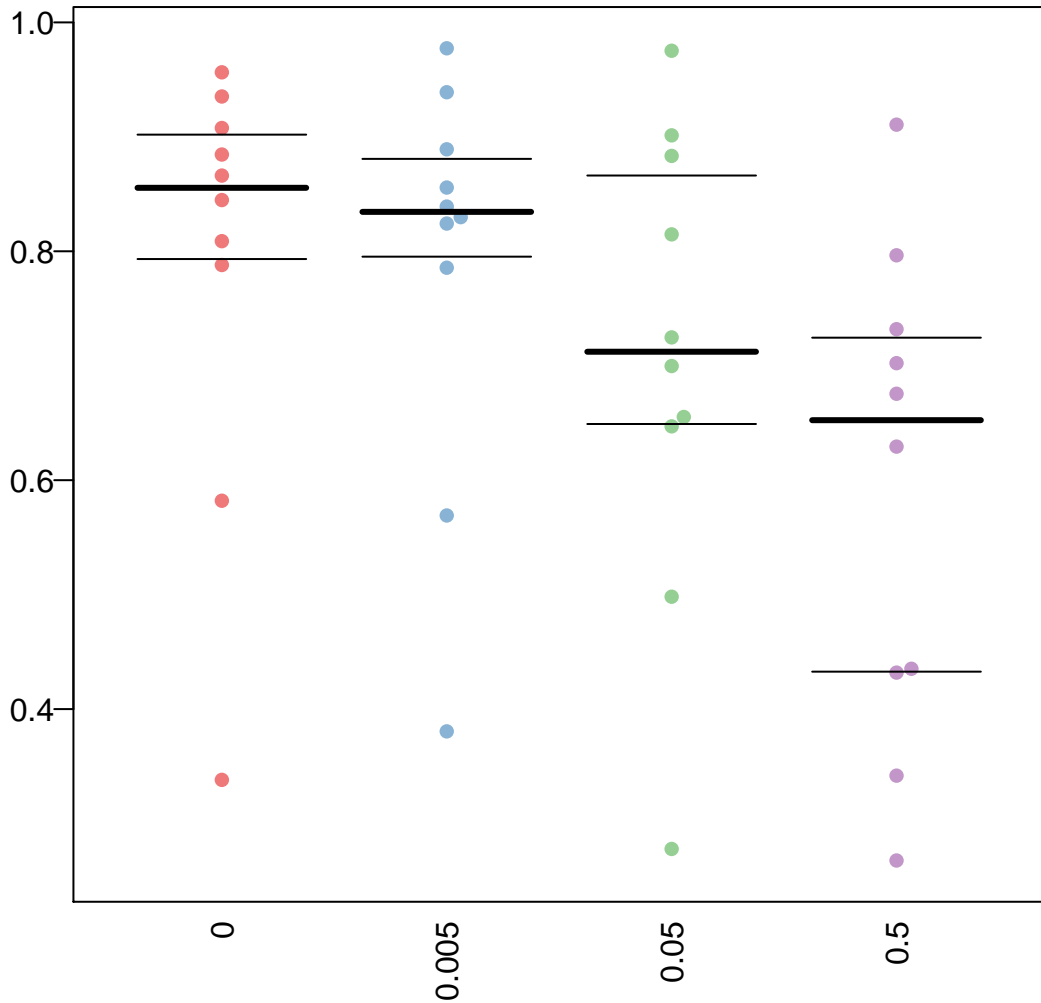

Supplement: Supplementary file 2 [file Data_Sheet_2.zip › predicted_phenotypes_SoyL/Anaerobic.pdf]

Relative Abundance

0.7  
0.6  
0.5  
0.4  
0.3  
0.2  
0.1  
0.0

0

0.005

0.05

0.5

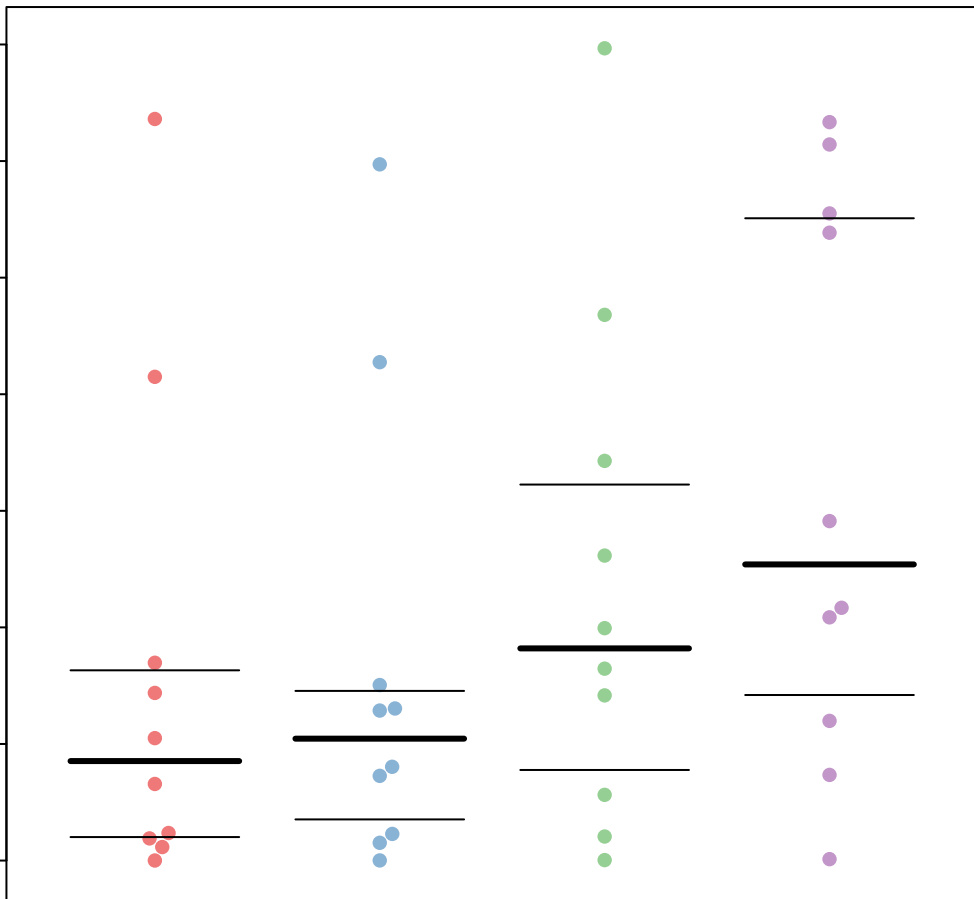

Supplement: Supplementary file 2 [file Data_Sheet_2.zip › predicted_phenotypes_SoyL/Contains_Mobile_Elements.pdf]

Relative Abundance

0.6  
0.4  
0.2  
0.0

0

0.005

0.05

0.5

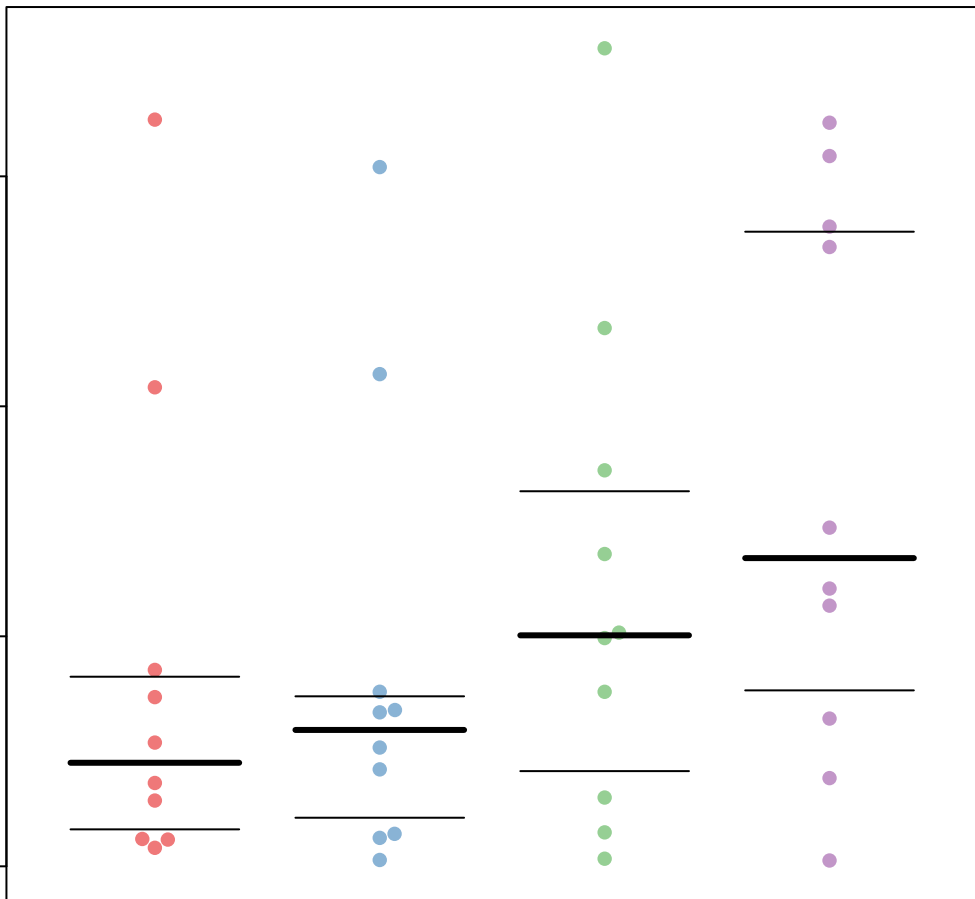

Supplement: Supplementary file 2 [file Data_Sheet_2.zip › predicted_phenotypes_SoyL/Facultatively_Anaerobic.pdf]

Relative Abundance

0.6

0.4

0.2

0

0.005

0.05

0.5

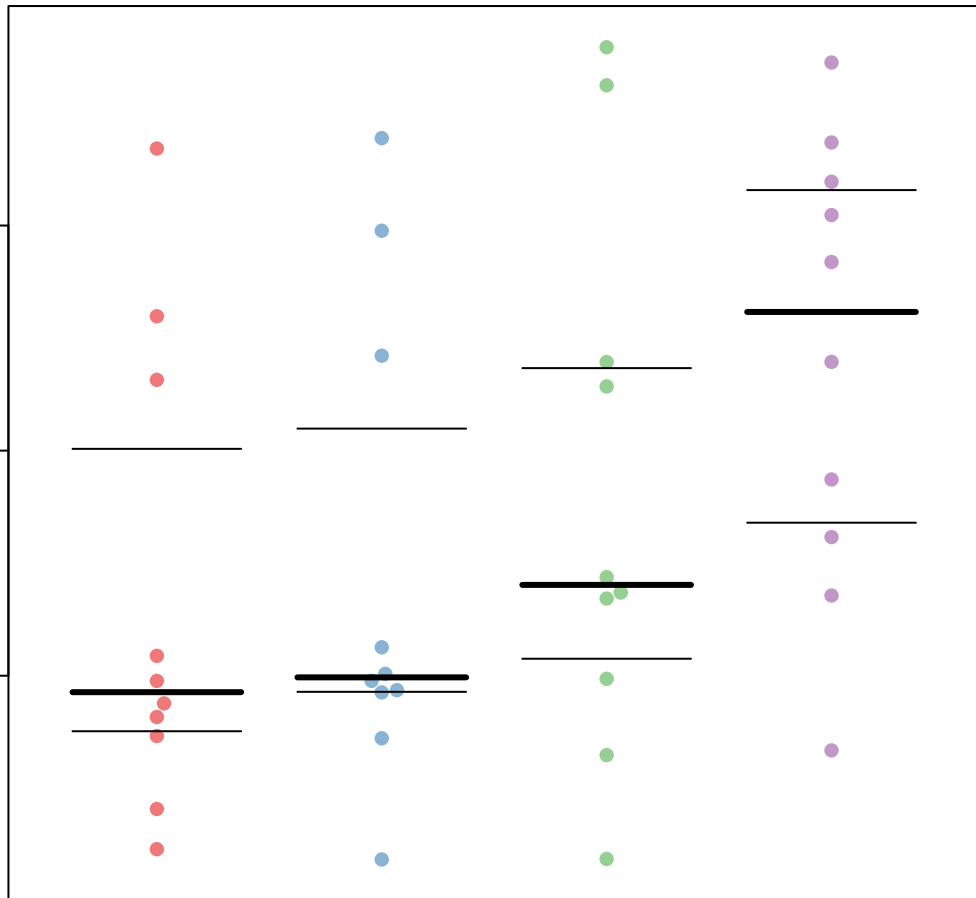

Supplement: Supplementary file 2 [file Data_Sheet_2.zip › predicted_phenotypes_SoyL/Forms_Biofilms.pdf]

Relative Abundance

0.9  
0.8  
0.7  
0.6  
0.5  
0.4  
0.3

0

0.005

0.05

0.5

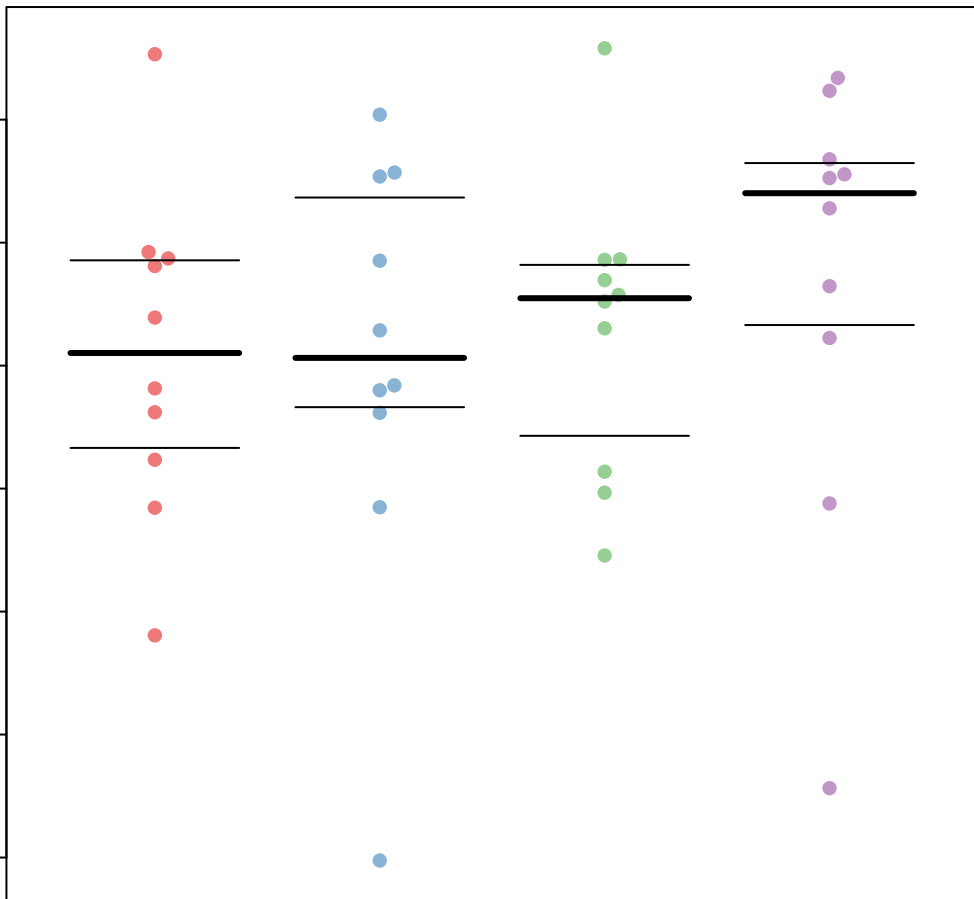

Supplement: Supplementary file 2 [file Data_Sheet_2.zip › predicted_phenotypes_SoyL/Gram_Negative.pdf]

Relative Abundance

0.7  
0.6  
0.5  
0.4  
0.3  
0.2  
0.1

0

0.005

0.05

0.5

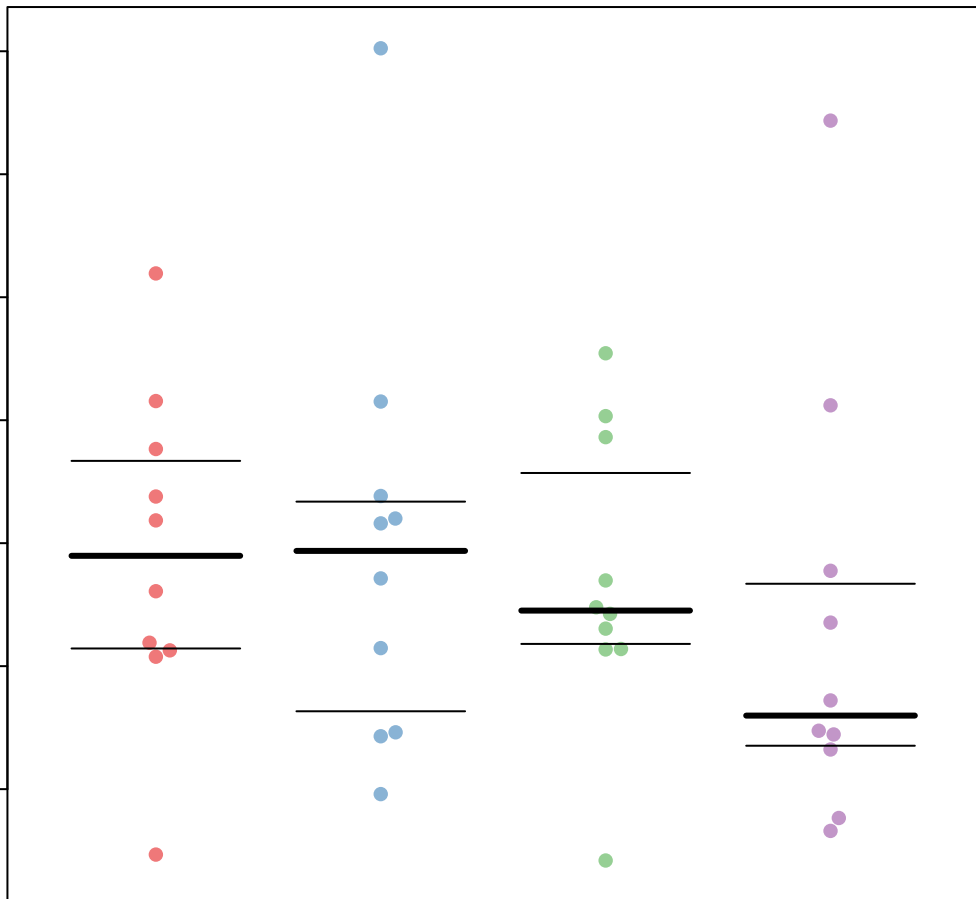

Supplement: Supplementary file 2 [file Data_Sheet_2.zip › predicted_phenotypes_SoyL/Gram_Positive.pdf]

Relative Abundance

0.6  
0.4  
0.2  
0.0

0

0.005

0.05

0.5

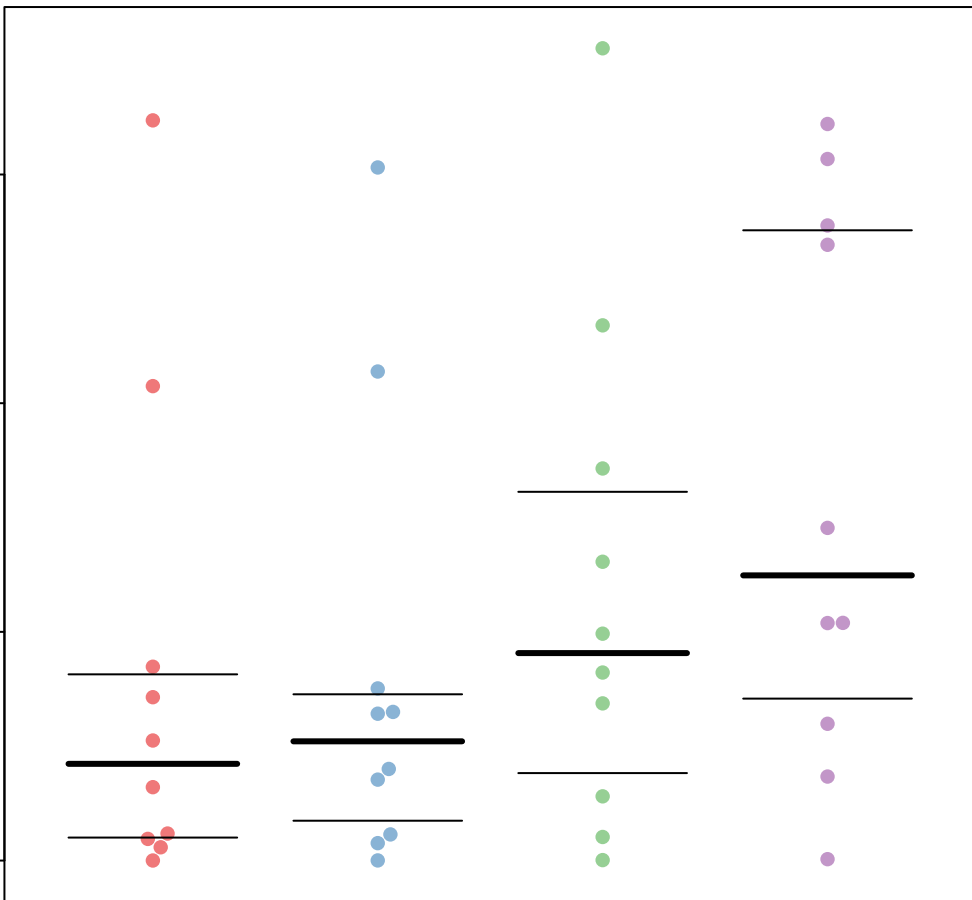

Supplement: Supplementary file 2 [file Data_Sheet_2.zip › predicted_phenotypes_SoyL/Potentially_Pathogenic.pdf]

Relative Abundance

0.6  
0.4  
0.2  
0.0

0

0.005

0.05

0.5

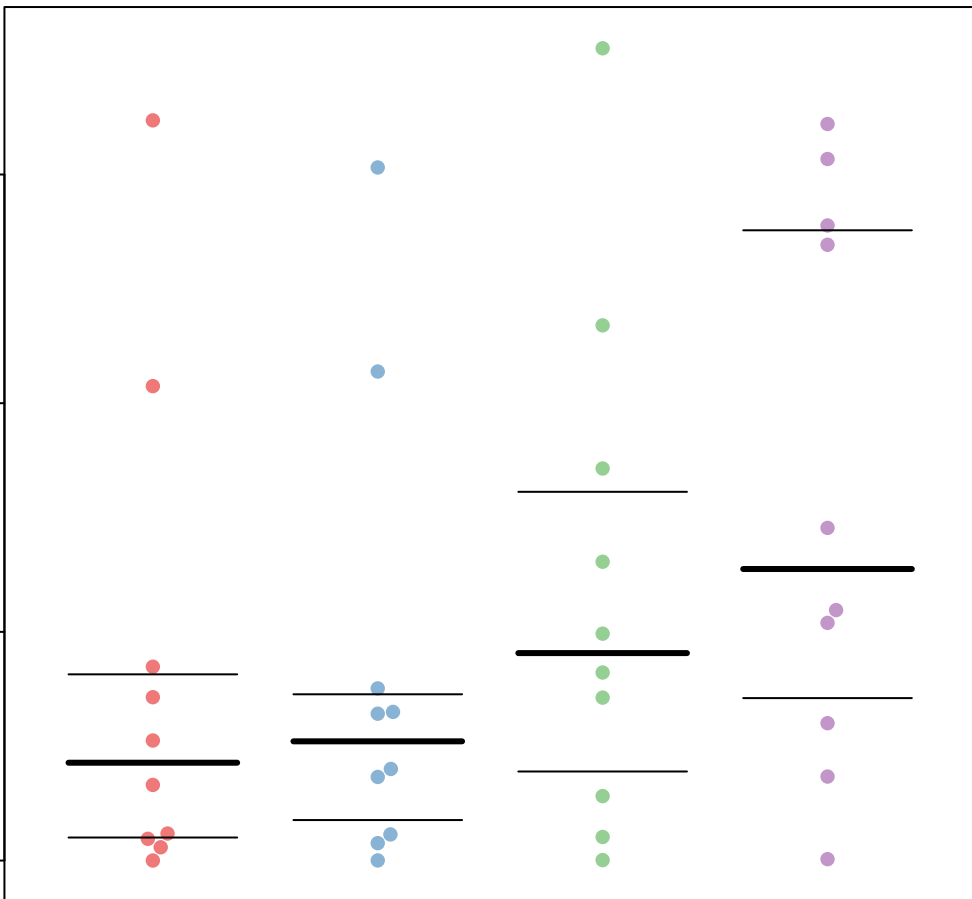

Supplement: Supplementary file 2 [file Data_Sheet_2.zip › predicted_phenotypes_SoyL/Stress_Tolerant.pdf]
